# Association of Oncologist-Patient Communication With Functional Status and Physical Performance in Older Adults: A Secondary Analysis of a Cluster Randomized Clinical Trial

**DOI:** 10.1001/jamanetworkopen.2022.3039

**Published:** 2022-03-18

**Authors:** Marielle Jensen-Battaglia, Lianlian Lei, Huiwen Xu, Lee Kehoe, Amita Patil, Kah Poh Loh, Erika Ramsdale, Allison Magnuson, Amber S. Kleckner, Tanya M. Wildes, Po-Ju Lin, Karen M. Mustian, Gilbert Giri, Mary Whitehead, James Bearden, Brian L. Burnette, Jodi Geer, Supriya G. Mohile, Richard F. Dunne

**Affiliations:** 1 James P. Wilmot Cancer Institute, University of Rochester Medical Center, Rochester, New York; 2 Department of Public Health Sciences, University of Rochester School of Medicine and Dentistry, Rochester, New York; 3 Department of Psychiatry, University of Michigan, Ann Arbor; 4 Sealy Center on Aging, Department of Preventive Medicine and Population Health, University of Texas Medical Branch, Galveston; 5 Johns Hopkins University School of Nursing, Baltimore, Maryland; 6 School of Nursing, Department of Pain and Translational Symptom Science, University of Maryland, Baltimore; 7 Cancer and Aging Research Group, St Louis, Missouri; 8 Division of Supportive Care in Cancer, Department of Surgery, University of Rochester Medical Center, Rochester, New York; 9 SCOREboard Advisory Group, University of Rochester Medical Center, Rochester, New York; 10 Upstate Carolina National Cancer Institute Community Oncology Research Program, Spartanburg, South Carolina; 11 Cancer Research of Wisconsin and Northern Michigan National Cancer Institute Community Oncology Research Program, Green Bay; 12 Metro Minnesota Community Oncology Research Program National Cancer Institute Community Oncology Research Program, St Louis Park

## Abstract

**Importance:**

The functional status and physical performance of older adults with cancer are underassessed and undertreated despite the high prevalence of impaired functional status and physical performance in this population and their associations with chemotherapy-induced toxic effects and mortality.

**Objective:**

To examine the association between providing oncologists with a geriatric assessment (GA) summary with recommendations and having oncologist-patient conversations about functional and physical performance.

**Design, Setting, and Participants:**

Data for this secondary analysis were collected from October 29, 2014, to April 28, 2017, for a national cluster randomized clinical trial conducted by the University of Rochester Cancer Center National Cancer Institute Community Oncology Research Program evaluating the effect of a GA intervention on patient satisfaction with communication about aging-related concerns. There were 17 practice clusters in the intervention group and 14 in the usual care group. All 541 participants underwent a GA including standardized functional and physical performance measures and had 1 clinical encounter audio-recorded, transcribed, and blindly coded to categorize conversations by GA domain. Participants were aged 70 years or older, with a stage III or IV solid tumor or lymphoma with palliative treatment intent, and impairment in 1 or more GA domain. Statistical analysis was performed from August 18, 2020, to January 10, 2022.

**Interventions:**

Oncologist practices randomized to the intervention received a GA summary and validated recommendations for each patient prior to the audio-recorded clinical encounter.

**Main Outcomes and Measures:**

The primary analysis of this clinical trial assessed the effect of the intervention on patient satisfaction with oncologist communication about aging-related concerns. This secondary analysis assessed the post hoc hypothesis that the intervention would be associated with an increase in the proportion of patients having conversations with their oncologists and receiving oncologist recommendations specific to functional and physical performance concerns.

**Results:**

A total of 541 patients (276 men [51%]; mean [SD] age, 77.5 [5.2] years [range, 70-96 years]) were analyzed at baseline. Excluding 13 patients without audio recordings, 86% of patients (95% CI, 78%-91%) in the intervention group vs 59% of patients (95% CI, 47%-69%; *P* < .001) receiving usual care had conversations about functional or physical performance. Conversations were more frequently initiated by oncologists in the intervention group (84%; 95% CI, 77%-90%) than oncologists in the usual care group (58%; 95% CI, 45%-70%; *P* < .001). Oncologists in the intervention group were more likely to address patients’ concerns (43%; 95% CI, 33%-53%) than oncologists in the usual care group (17%; 95% CI, 10%-26%; *P* < .001).

**Conclusions and Relevance:**

In this secondary analysis of a cluster randomized clinical trial, providing oncologists with a GA summary was associated with an increase in the number of oncologist-patient conversations about functional and physical performance–related concerns with recommendations to address these concerns. These findings support the use of the GA summary and recommendations as important tools in caring for older adults with advanced cancer and functional or physical impairments.

**Trial Registration:**

ClinicalTrials.gov Identifier: NCT02107443

## Key Points

**Question:**

Is there an association between oncologists being provided with a summary of a geriatric assessment and tailored recommendations and patients having conversations and receiving recommendations regarding their functional status and physical performance?

**Findings:**

This secondary analysis of data from a cluster randomized clinical trial of 541 patients found that a greater proportion of patients discussed physical performance or functional status concerns with their oncologists when those oncologists received the geriatric assessment summary and recommendations compared with patients who received usual care.

**Meaning:**

This study suggests that incorporating geriatric assessment results and recommendations into routine community oncology practice may promote oncologist-patient discussions of physical performance and functional status.

## Teaser

This secondary analysis of a cluster randomized clinical trial examines the association between providing oncologists with a geriatric assessment summary with recommendations and having oncologist-patient conversations about functional and physical performance.

## Introduction

Older adults account for an increasing proportion of patients with cancer in the US, and 70% of all cancers are expected to be diagnosed among those aged 65 years or older by 2030.^1^ In response to this demographic shift, clinicians must improve their understanding of the range of challenges faced by older patients with cancer. Impairments in functional status and physical performance are 2 such challenges common across all stages of cancer care.^1,2,3,4,5,6,7^ In addition to their association with cancer treatment outcomes, functional status and physical performance are highly prioritized by patients and caregivers as key factors in making treatment-related decisions.^8,9,10^

Functional status—the ability to complete activities of daily living (ADLs) such as bathing, dressing, toileting, transfers, continence, and feeding^11^ and instrumental activities of daily living (IADLs) such as cooking, cleaning, and shopping^12^—is typically assessed via patient or caregiver report. Impairments in functional status are associated with increased cancer-related morbidity,^13^ chemotherapy toxicity, and decreased survival,^14^ but this measure fails to detect early stages of disability when adaptation and accommodation preserve independence despite a decrease in physical capacity.^15,16^ Physical performance reflects physical capacity, often measured using standardized physical tasks (such as gait speed or timed repeated sit-to-stand).^17^ Physical performance is associated with future declines in functional status^18,19^ and with treatment tolerability,^20^ treatment complications, and overall survival.^21^ Self-report measures, such as the Older Americans Resources and Services–Physical Health Scale^22^ and fall history, have also been included in this domain because they reflect impairments in physical performance, such as balance, strength, and endurance.^23,24,25,26^ A combination of functional status and physical performance measures has been shown to be associated with health outcomes, including decreased health-related quality of life,^27^ better than either alone.^28^ When cancer is incurable, consideration of both functional status and physical performance is critical to align treatment decisions with patient-centered goals because many older adults with limited life expectancy prioritize function and independence over survival.^8^

Despite the importance of functional and physical impairments in cancer care, routine oncology evaluation often fails to adequately assess functional status and physical performance concerns.^19^ Commonly used physician-reported scales, such as the Karnofsky Performance Status (KPS) and the Eastern Cooperative Oncology Group Performance Status, were developed and validated in younger cohorts and do not fully account for physical performance.^29,30^ Studies show that older adults with good performance status may be limited in ADLs, IADLs, or objective physical performance tests.^31,32^ Older adults with cancer have complex medical presentations, treatment considerations, and goals of care,^33,34^ leaving standardized functional status and physical performance assessments at risk for being deprioritized in busy clinic settings with limited resources.^35^ Finally, even when oncologists recognize the importance of assessing and managing functional and physical impairments, most do not feel confident in doing so.^36^

A geriatric assessment (GA) can identify and guide the management of aging-related impairments among older adults with cancer^36,37,38^ and includes valid and reliable measures of functional status and physical performance that are more sensitive to impairments in these domains than a routine assessment.^39^ A GA assists in clinical decision-making^36,37^ and is recommended by the American Society of Clinical Oncology for patients aged 65 years or older with cancer.^37,40^

Although the primary analysis of the present secondary analysis of a cluster randomized trial showed that oncologists’ knowledge of a GA summary led to improvements in overall oncologist-patient communication,^41^ data are lacking that assess how oncologists discuss impairments specific to functional status and physical performance with older patients with advanced cancer and how the use of a GA may affect this discussion. We hypothesized that a GA intervention would be associated with an increased frequency of conversations and recommendations to address concerns. This improved communication could be associated with an increase in the quality of management for age-related functional status and physical performance concerns. We used data from a nationwide randomized clinical trial conducted in community oncology practices to conduct a secondary analysis evaluating whether a GA intervention is associated with improved communication regarding functional status and physical performance impairments among older adults with advanced cancer.

## Methods

### Data Source

This is a secondary analysis of data from the Improving Communication in Older Cancer Patients and Their Caregivers (COACH) trial (NCT02107443). Data for this nationwide, cluster randomized clinical trial conducted by the University of Rochester Cancer Center National Cancer Institute Community Oncology Research Program were collected from October 29, 2014, to April 28, 2017. Demographic characteristics, including self-reported race and ethnicity, and the results of both primary (patient satisfaction with oncologist communication about aging-related concerns) and secondary (caregiver satisfaction with communication about aging-related concerns, patient quality of life, and number of aging-related concerns discussed) outcomes were previously published.^41^ The primary outcome for this secondary analysis was the proportion of patients who had a conversation with their oncologist about physical performance or functional status aging related concerns. A total of 541 patients aged 70 years or older with an advanced solid malignant neoplasm or lymphoma undergoing cancer treatment with palliative intent were enrolled after providing written informed consent. Each participating practice site (eTable 1 in Supplement 1) obtained approval from their respective institutional review boards, and community oncology practices were randomized to either usual care (n = 14) or GA intervention (n = 17) groups. All patients underwent a GA at baseline, and oncologists in the intervention group received a full GA summary and a list of GA-associated management recommendations to address impairments in functional status or physical performance (Box). In contrast, oncologists in the usual care group did not receive the GA summary and were notified only of depression or severe cognitive impairment. Karnofsky Performance Status^42^ was reported by oncologists in addition to GA measures. For both study groups, 1 oncology visit within 4 weeks of a GA was audio-recorded, transcribed, and analyzed by blinded coders who underwent 40 hours of in-person training with study investigators and reached more than 70% interrater reliability on training transcripts before coding study transcripts. Conversations were categorized by functional status or physical performance domains (eTable 2 in Supplement 1) by at least 2 independent coders, with an interrater reliability of 82% for the number of aging-related concerns. See the trial protocol in Supplement 2 and the eAppendix in Supplement 3 for full details. The 2 study groups (248 patients in the usual care group and 293 patients in the intervention group) were comparable in demographic characteristics.^43,44^ The Consolidated Standards of Reporting Trials (CONSORT) reporting guideline^45^ was adhered to in the initial publication of these trial results and in this secondary analysis.

Box. **Oncologist Recommendations Used to Address Functional Status and Physical Performance Concerns: Categories and Responses**

**Referrals**

Physical therapy
Occupational therapy
Aide services
Home nursing services
Personal emergency response system
Low-vision specialist
Hearing specialist
Requested gait or assistive device evaluation, strength and balance training

**Physical examination**

Check orthostatic blood pressure
Decrease blood pressure medications

**Medication review**

Minimize psychoactive and duplicative medications

**Treatment modification**

Single agent rather than combination therapy
Modify regimen
Modify treatment dosage
Conduct toxic effects check
Choose nonneurotoxic regimen (if available)

**Information**

Fall counseling handout
Energy conservation handout
Exercise prescription handout

### Functional Status and Physical Performance

Functional status measures included IADLs (score range, 0-7, where ≥1 IADL reported as impaired)^46^ and ADLs (score range, 0-6, where ≥1 ADL reported as impaired).^11^ Physical performance measures included the Timed Up and Go test (>13.5 seconds reported as impaired),^47^ the Short Physical Performance Battery (score range, 3-12; ≤9 points reported as impaired),^48^ the Older Americans Resources and Services–Physical Health scale (≥1 responses for “my health limits me a lot” reported as impaired),^49^ and history of falls (≥1 fall within the previous 6 months reported as impaired).^50^

### Conversations

Conversations pertained to functional status if patients and oncologists had any discussion of the need for assistance in any ADL or IADL. Physical performance conversations included the discussion of mobility concerns, such as standing tolerance, exercise, walking, getting in and out of a chair, balance, strength, falls, stair climbing, and sensory deficits (eTable 2 in Supplement 1). Concerns were considered “unspecified” when they were clearly associated with a category but not a specific concern. Because oncologists may not distinguish between physical performance and functional status concerns as operationalized in their communication with patients, similar categories of concerns in functional status and physical performance domains were combined into 8 clinical themes for exploratory analysis. For example, difficulty getting in and out of a bed or a chair (physical performance) and getting in and out of a chair (functional status) were collapsed into “transfers.”

The initiator of the conversation (patient, caregiver, or oncologist) and the oncologist response were noted. Oncologists were coded as dismissing concerns if they ignored, shut down, or minimized the concern; as acknowledging the concern if they noted the concern but did not implement any care processes; and as addressing the concern if they implemented appropriate recommendations to respond to the concern (eTable 2 in Supplement 1). Addressed concerns were further coded to reflect the type of recommendation discussed, which were categorized a priori as referrals, physical examination, treatment modification, or information and education (Box).

### Statistical Analysis

Statistical analysis was performed from August 18, 2020, to January 10, 2022. In this post hoc analysis, frequencies and proportions of baseline characteristics were reported by study group. To reduce multiple comparisons and maximize external validity, conversations about physical performance or functional status were primarily analyzed as a combined group. Proportions of overall conversations, conversation initiation, and oncologist response to functional status and physical performance concerns were calculated using linear mixed models adjusted for practice site as a random effect, with differences assessed by the LSMEANS procedure. All *P* values were from 2-sided tests, with *P* < .05 considered statistically significant. Data analyses were completed using SAS software, version 9.4 (SAS Institute Inc).

## Results

### Study Sample Characteristics

**Figure 1.**
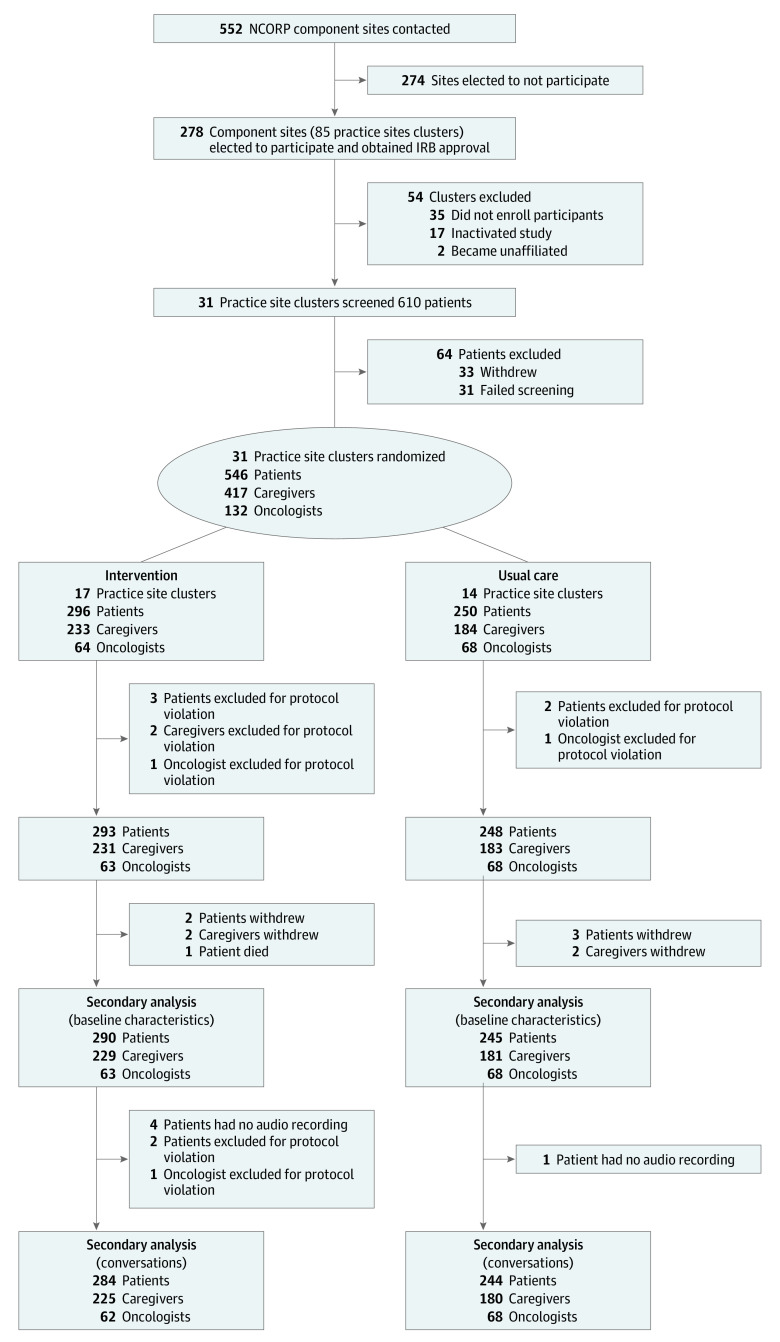
Study Flow Diagram IRB indicates institutional review board; and NCORP, National Cancer Institute Community Oncology Research Program.

All 541 patients (276 men [51%]) enrolled were included in this secondary analysis. Patients in the overall sample had a mean (SD) age of 77.5 (5.2) years (range, 70-96 years), were predominantly White (482 [89%]), well educated (279 of 540 [52%] with some college), and married (348 [64%]), and about half had an income of $50 000 or less per year (265 of 538 [49%]). Although only 113 of 539 patients (21%) had a KPS score less than 80 (representing potentially impaired physical function),^51^ levels of functional or physical impairment as measured by the GA were high; 319 patients (59%) were impaired in functional status, 507 (94%) were impaired in physical performance, and 314 (58%) were impaired in both. The intervention and usual care groups were comparable in the levels of impairment on KPS and GA measures except for the Timed Up and Go test and Short Physical Performance Battery, where the usual care group had a higher proportion of impairment (Timed Up and Go test: intervention, 97 of 293 [33%] vs usual care, 111 of 248 [45%]; and Short Physical Performance Battery: intervention, 218 of 293 [74%] vs usual care, 210 of 248 [85%]) (Table 1). A KPS score less than 80 was associated with impairments in physical performance, with 110 of 505 patients (22%) impaired in physical performance also having a KPS score less than 80, compared with 3 of 34 patients (9%) who were unimpaired (*P* = .03). However, there was not a statistically significant association between KPS score and functional status impairment, with 23% (73 of 318) of those impaired in functional status also having a KPS score less than 80, compared with 18% (40 of 221) of those without functional impairments (*P* = .17). Because of death, technical difficulty, protocol violation, or voluntary withdrawal, the audio recordings of 528 patients were included for analysis of conversations (2% missingness; see Figure 1 for study flow diagram).

**Table 1.** Percentage of Patients Who Met the Impairment Cutoff for Physical Performance and Functional Status as Assessed Using the GA

GA domain, measure^a^	Patients, No. (%)		
	All (N = 541)	Intervention group (n = 293)	Usual care group (n = 248)
Physical performance			
TUG	208 (38)	97 (33)	111 (45)
SPPB	428 (79)	218 (74)	210 (85)
OARS-PH	406 (75)	222 (76)	184 (74)
Falls	140 (26)	82 (28)	58 (23)
Any 1 of the above	507 (94)	268 (92)	239 (96)
Any 2 of the above	149 (28)	75 (26)	74 (30)
Functional status			
IADL	303 (56)	167 (57)	136 (55)
ADL	149 (28)	75 (26)	74 (30)
Any 1 of the above	319 (59)	174 (59)	145 (59)
Both of the above	133 (25)	68 (23)	65 (26)
Any 1 of the above	512 (95)	272 (93)	240 (97)
Any 2 of the above	407 (75)	213 (73)	194 (78)
Both physical performance and functional status	314 (58)	170 (58)	144 (58)

Abbreviations: ADL, activity of daily living; IADL, instrumental activity of daily living; GA, geriatric assessment; OARS-PH, Older Americans Resources and Services–Physical Health scale; SPPB, Short Physical Performance Battery; TUG, Timed Up and Go test.

^a^ Cutoff values for each measure: TUG, more than 13.5 seconds; SPPB, 9 points or less; OARS-PH, response of “a lot” for any of the 10 activities; falls (yes to ≥1 fall within 6 months); IADL, 1 or more; and ADL, 1 or more.

### Conversations

Compared with the usual care group, the intervention group had a greater number of conversations regarding both functional status (164 vs 87) and physical performance (532 vs 183). Excluding 13 patients without audio recordings, 86% of patients (95% CI, 78%-91%) in the intervention vs 59% of patients (95% CI, 47%-69%; *P* < .001) receiving usual care had conversations about functional or physical performance. After adjustment for practice site, 89% of patients (95% CI, 82%-94%) in the intervention group vs 60% of patients (95% CI, 46%-73%) receiving usual care had conversations about functional status or physical performance concerns (*P* < .001). When limiting the sample to those with a KPS score less than 80, a greater proportion of patients in the intervention group (88%; 95% CI, 77%-94%) had conversations regarding physical performance and functional status compared with those in the usual care group (63%; 95% CI, 48%-75%; *P* = .004).

Unadjusted frequencies of conversations by specific concern are shown in Table 2. Falls were the most discussed concern overall (20%), followed by difficulty with walking (12%). Oncologists initiated a greater proportion of all physical performance and functional status conversations in the intervention group (84%; 95% CI, 77%-89%) compared with the usual care group (58%; 95% CI, 45%-70%; *P* < .001) (eTable 3 in Supplement 1). Patients and caregivers initiated differing proportions of conversations (42% [95% CI, 30%-55%] in the intervention group and 16% [95% CI, 11%-23%] in the usual care group; *P* < .001) but a similar unadjusted number of conversations (intervention, 118 vs usual care, 117) across study groups, which suggests that the intervention was not associated with reduced patient- and caregiver-initiated concerns despite an increase in the number initiated by oncologists. This pattern was similar when patients were impaired only in functional status, only in physical performance, or in both.

**Table 2.** Unadjusted Frequencies and Proportions of Physical Performance and Functional Status Concerns by Clinical Theme

Clinical theme	Concerns, No. (%)			Specific concerns
	All (N = 966)	Intervention group (n = 696)	Usual care group (n = 270)	
ADLs	115 (12)	82 (12)	33 (12)	Bathing, getting dressed, eating, using the toilet, ADLs
Balance	103 (11)	83 (12)	20 (7)	Balance or unsteadiness, unsteadiness
Exercise	56 (6)	34 (5)	22 (8)	Exercise
Falls	191 (20)	168 (24)	23 (9)	Falls
IADLs	53 (6)	36 (5)	17 (6)	Using the telephone, going shopping (for clothes or groceries), taking medication, managing finances, driving
Transfers	21 (2)	12 (2)	9 (3)	Getting up or sitting down from a chair, getting out of bed or chairs
					Walking	119 (12)	76 (11)	43 (16)	Walk any distance, walking
Weakness	76 (8)	51 (7)	25 (9)	Strength (weakness)
No clinical theme	4 (0.4)	2 (0.3)	2 (1)	Ability to stand for long periods
	86 (9)	68 (10)	18 (7)	Vision
	142 (15)	84 (12)	58 (22)	Unspecified or other
Total	966 (100)	696 (100)	270 (100)	NA

Abbreviations: ADLs, activities of daily living; IADLs, instrumental activities of daily living; NA, not applicable.

**Figure 2.**
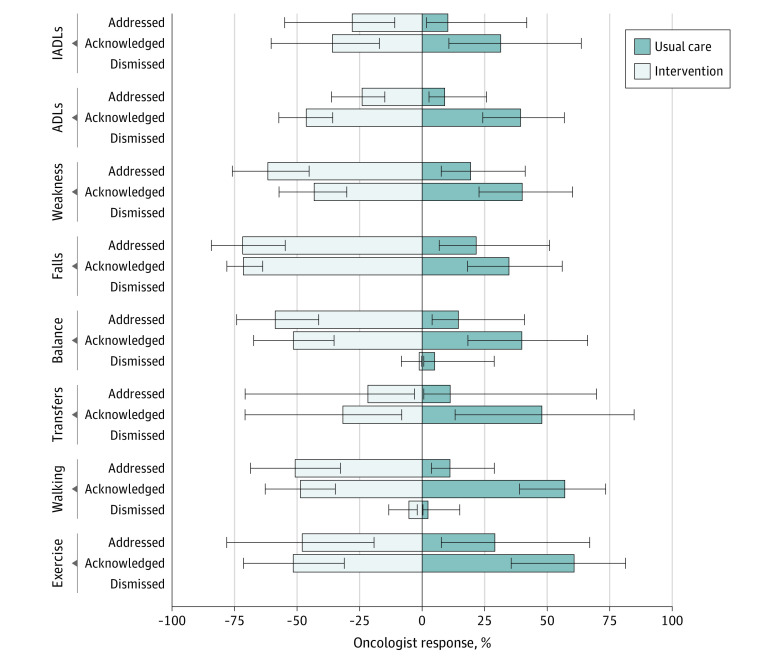
Physical Performance and Functional Status Concerns and Oncologist Response Adjusted proportions were generated using linear mixed models with practice site as a random effect. Bars are absent (ie, instrumental activities of daily living [IADLs] dismissed) where the outcome occurred too infrequently and statistics could not be estimated. ADLs indicate activities of daily living. Error bars indicate 95% CIs.

Oncologists dismissed (1% [95% CI, 1%-3%] in the intervention group and 3% [95% CI, 1%-6%] in the usual care group; *P* = .17) and acknowledged (53% [95% CI, 46%-59%] in the intervention group and 46% [95% CI, 38%-54%] in the usual care group; *P* = .19) physical performance and/or functional status concerns at similar rates in both groups (eTable 4 in Supplement 1). However, significantly more concerns were addressed in the intervention group (43%; 95% CI, 33%-53%) than in the usual care group (17%; 95% CI, 10%-26%; *P* < .001). This pattern was the same for concerns when analyzed within the individual functional status and physical performance domains, although it reached statistical significance only in the physical performance domain. When functional status and physical performance concerns were combined into 8 clinical themes (Table 2), oncologists in the intervention group were significantly more likely to acknowledge balance concerns and address concerns related to balance, falls, and weakness (Figure 2).

### Management

As an exploratory analysis, narrative examples of each type of recommendation alongside proportions of concerns addressed with that recommendation by study group are shown in Table 3. Referrals (Box) were the most common oncologist recommendation for both functional status and physical performance, occurring for 24% of concerns (95% CI, 18%-30%) in the intervention group vs 5% of concerns (95% CI, 3%-9%) in the usual care group (*P* < .001) (eTable 5 in Supplement 1). Provision of information and education was used to address 22% of concerns (95% CI, 14%-34%) by intervention oncologists and 4% of concerns (95% CI, 2%-9%) by usual care oncologists (*P* < .001). There were no significant differences between intervention oncologists and usual care oncologists in recommendations made for physical examination (0.6% [95% CI, 0.2%-2%] vs 1% [95% CI, 0.4%-3%]) or treatment modification (1% [95% CI, 0.5%-2%] vs 0.4% [95% CI, 0.05%-3%]).

**Table 3.** Joint Display Including Narrative Examples of Interventions Referenced to Address Functional Status and Physical Performance Concerns and Quantitative Results of Proportions^a^

Exemplar functional status or physical performance concern	Exemplar oncologist response	Response category	Adjusted proportion (95% CI)^b^		*P* value
			Intervention	Usual care	
Oncologist: “…how do you feel in terms of walking in your neighborhood?”	Oncologist: “…if necessary we can make an appointment with physical therapy to help you be more mobile and to feel better.”	Referrals	24 (18-30)	5 (3-9)	<.001
Oncologist: “…we were worried about you falling.”	Oncologist: “So you are about 130/86 lying down. Let’s get you to a chair and sit down. Scoot back just a little bit. Are you dizzy now?”	Physical examination	0.6 (0.2-2)	1 (0.4-3)	.39
Oncologist: “…worried about you may be at, you know, a risk of falling. You know, physical weakness.”	Oncologist: “…because of your age and, you know, other medical conditions you are at risk of, you know, increased toxicity from the chemotherapy…. So if you feel like the treatment is making the quality of life worse we need to back off or cut down.”	Treatment modification	1 (0.5-2)	0.4 (0.05-3)	.35
Oncologist: “Tell me about balance and the feeling that you are going to fall.”	Oncologist: “Well, I want to give you some written information and these are handouts about the benefits of exercise…”	Information and education	22 (14-34)	4 (2-9)	<.001
Oncologist: “…three areas of some concern based on the geriatric assessment. Probably the most important one, in your case, is a fall risk.”	Oncologist: “…also, was polypharmacy meaning if you have—if you’re on more than 5 medications. And you are but I looked at them all and I think they’re all appropriate.”	Medication review	Statistics not possible^c^		

^a^ Percentages reflect the percent of concerns addressed using each recommendation and do not sum to 100.

^b^ Adjusted proportions generated using linear mixed models with practice site as a random effect.

^c^ Outcome occurred 0 times in usual care group, and therefore statistics were not possible.

## Discussion

Our findings confirm the high prevalence of functional status and physical performance impairments among older adults with cancer.^4,5,7,52^ Even though most patients in both study groups presented with impairments, only 59% of patients in the usual care group had a conversation with their oncologist regarding their impaired functional status or physical performance compared with 86% of intervention group patients whose oncologists received a GA summary with tailored recommendations. This association was preserved even in the subgroup of patients with an oncologist-rated KPS score less than 80, which suggests that the addition of a GA may promote conversations even for patients already assessed as functionally impaired by their treating physicians. We found that most patients classified as impaired by GA measures of physical performance or functional status were classified as unimpaired by their KPS scores, consistent with studies showing that the KPS score is less sensitive to impairments than objective measures of physical performance.^32,39^

This intervention has previously been shown to be associated with an increase in conversations about comorbidity.^53^ In a subset of participants, the intervention and usual care groups discussed polypharmacy with similar frequency^54^; however, oncologists initiated conversations about polypharmacy more frequently in the intervention group, echoing our finding in functional status and physical performance domains. In this secondary analysis, a similar number of patients and caregivers in the intervention group voiced their concerns in each group, which may reflect the importance of these domains to patients. Higher frequencies of conversations about falls and ADLs in the intervention group suggest that oncologists experienced a benefit associated with being prompted by the GA summary to discuss relevant topics for older adults who might otherwise receive less attention.

The GA intervention in this trial also prompted oncologists to address aging-related functional status and physical performance concerns during the clinic visit. Intervention oncologists were more likely to recommend referrals and provide information-based interventions suggested by the GA summary, and GA findings were referenced in conversations with patients and caregivers, demonstrating the utility of GA as a tool in developing tailored interventions for concerns in these areas. Implementation of GA-based recommendations for functional status and physical performance impairments, such as treatment modification and referral to rehabilitation services, is critical because these interventions have been shown to be associated with improving quality of life and reducing risk for functional decline and death.^55,56^ Patient care informed by GA also reduces the risk for falls,^57^ unplanned hospitalizations, nursing home admissions, and overall disability.^38,58,59,60^ Home care services addressing GA impairments have previously been shown to reduce nursing home admissions and functional decline in older adults.^61,62^ Communication surrounding recommendations is critical because GA-guided management recommendations deemed important by physicians or agreed on by the patient and physician are more likely to be implemented.^63,64^ In the primary analysis of this trial, improvements in patient satisfaction with communication about aging-related concerns in the intervention group were found to persist over a 6-month follow-up,^41^ which may translate to a higher rate of implementation of recommendations and improved patient outcomes.

To our knowledge, this study is the first to evaluate the association of a GA intervention with oncologist-patient communication surrounding functional status and physical performance in a national sample of older adults with cancer. Participation of National Cancer Institute Community Oncology Research Program sites supplied clinical practice settings, and cluster randomization reduced the potential for participant selection bias. A thorough content analysis allowed for the characterization of conversations by initiator, topic, and oncologist response. Although this intervention has been shown to increase the overall number of conversations regarding aging-related concerns,^41^ this secondary analysis provides further insight into conversations related to functional status and physical performance, which may have broad and deep implications not only for the cancer treatment trajectory but also for the patient’s overall quality of life. Our findings show that a 1-time GA intervention can enhance oncologist-patient communication regarding these critical aging-related concerns, better aligning oncology care with patient priorities.

### Limitations

Our analysis has several limitations. The audio recording represents a single oncology visit, and topics including functional status and physical performance may have been discussed in subsequent patient encounters or with health care staff other than the oncologist. Although referrals and information were used more commonly than other recommendations, oncologists may have enacted treatment modifications, physical examinations, and medication management without openly discussing the association between functional status or physical performance and these interventions. However, the use of cluster randomization and audio recordings ensured that it was reasonable to assume that the differences between study groups were associated with the intervention. Finally, our analysis does not reflect interventions successfully performed to address impairments but rather those that were discussed by the oncologist directly with the patient. Further studies are needed to characterize changes in clinical outcomes associated with this GA intervention.

## Conclusions

This study found that the provision of a GA summary with tailored recommendations to the oncologist was associated with an increase in oncologist-initiated conversations pertaining to functional status and physical performance while promoting discussion of valid and reliable interventions to address concerns in these areas. Given the aging US population,^65^ the increasing prevalence of cancer, and the burden of functional status and physical performance impairments faced by older adults with cancer, these findings support the use of a GA and the provision of a GA summary to oncologists to direct oncologist-patient discussions of care regarding function and physical performance as recommended in American Society of Clinical Oncology clinical guidelines.

## Notes

Supplement 1. **eTable 1.** National Cancer Institute Community Oncology Research Program (NCORP) Community Affiliate Partners **eTable 2.** Physical Performance and Functional Status Concern Subcodes and Definitions **eTable 3.** Raw Frequencies and Adjusted Proportions of Who Initiated Functional Status and Physical Performance Conversations **eTable 4.** Raw and Adjusted Proportions of Oncologist Response to Functional Status and Physical Performance Concerns **eTable 5.** Raw and Adjusted Proportions of Recommendations Discussed to Address Functional Status and Physical Performance Concerns

Supplement 2.
Trial Protocol

Supplement 3.
eAppendix.

Supplement 4.
Data Sharing Statement
