# Supplementary material for: Association of Oncologist-Patient Communication With Functional Status and Physical Performance in Older Adults: A Secondary Analysis of a Cluster Randomized Clinical Trial
Source: JAMA Netw Open. 2022 Mar 18;5(3):e223039. doi: 10.1001/jamanetworkopen.2022.3039 (PMC8933739; doi:10.1001/jamanetworkopen.2022.3039)
Supplement: Supplement 1. — eTable 1. National Cancer Institute Community Oncology Research Program (NCORP) Community Affiliate Partners eTable 2. Physical Performance and Functional Status Concern Subcodes and Definitions eTable 3. Raw Frequencies and Adjusted Proportions of Who Initiated Functional Status and Physical Performance Conversations eTable 4. Raw and Adjusted Proportions of Oncologist Response to Functional Status and Physical Performance Concerns eTable 5. Raw and Adjusted Proportions of Recommendations Discussed to Address Functional Status and Physical Performance Concerns [file jamanetwopen-e223039-s001.pdf]

Supplementary Online Content

Jensen-Battaglia M, Lei L, Xu H, et al. Association of oncologist-patient communication with functional status and physical performance in older adults: a secondary analysis of a cluster randomized clinical trial. *JAMA Netw Open.* 2022;5(3):e223039. doi:10.1001/jamanetworkopen.2022.3039

eTable 1. National Cancer Institute Community Oncology Research Program (NCORP) Community Affiliate Partners
eTable 2. Physical Performance and Functional Status Concern Subcodes and Definitions
eTable 3. Raw Frequencies and Adjusted Proportions of Who Initiated Functional Status and Physical Performance Conversations
eTable 4. Raw and Adjusted Proportions of Oncologist Response to Functional Status and Physical Performance Concerns
eTable 5. Raw and Adjusted Proportions of Recommendations Discussed to Address Functional Status and Physical Performance Concerns

This supplementary material has been provided by the authors to give readers additional information about their work.

© 2022 Jensen-Battaglia M et al. *JAMA Network Open.*

eTable 1. National Cancer Institute Community Oncology Research Program (NCORP) Community Affiliate Partners

NCORP Community Affiliate
Aurora NCORP (Illinois, Wisconsin)
Columbus NCORP (Columbus, Ohio)
Delaware/Christiana Care (Newark, Delaware)
Geisinger Cancer Institute Oncology Research Program (Danville, Pennsylvania)
NCORP of the Carolinas (Greenville, South Carolina)
Hawaii Minority Underserved NCORP (Honolulu, Hawaii)
Heartland Cancer Research NCORP (Decatur, Illinois)
Metro-Minnesota NCORP (Saint Louis Park, Minnesota)
Michigan Cancer Research Consortium NCORP (Ann Arbor, Michigan)
Nevada Cancer Research Foundation NCORP (Las Vegas, Nevada)
Northwell Health NCORP (Lake Success, New York)
Pacific Cancer Research Consortium NCORP (Seattle, Washington)
Southeast Clinical Oncology Consortium NCORP (Winston- Salem, North Carolina)
Wisconsin NCORP (Marshfield, Wisconsin)
Wichita NCORP (Wichita, Kansas)

© 2022 Jensen-Battaglia M et al. *JAMA Network Open.*

eTable 2. Physical Performance and Functional Status Concern Subcodes and Definitions

**Type of functional status concern**	
Subcode	*Definition: any discussion of inability to do ____ independently*
Functional Status	Bathing, dressing, eating, getting out of bed/chair, walking, using toilet, using telephone, shopping, taking medication, managing finances, driving, activities of daily living, unspecified.
**Type of physical performance concern**	
Subcode	*Definition: any discussion regarding mobility, including…*
Physical Performance	Ability to stand for long periods, exercise, walk any distance, get up or sit down from a chair, difficulty with balance/unsteadiness, hearing, vision, falls, strength, stairs, unspecified.
**Quality of response to concern**	
Subcode	*Definition: the oncologist…*
Dismissed	Actively shut down, ignored, moved away from, or minimized the concern expressed
Acknowledged	The oncologists acknowledged the physical performance or functional status concern but did not implement any care processes.
Addressed	The oncologist implemented appropriate care processes to address the physical performance or functional status concern

© 2022 Jensen-Battaglia M et al. *JAMA Network Open.*

eTable 3. Raw Frequencies and Adjusted Proportions of Who Initiated Functional Status and Physical Performance Conversations

GA Domain	Who initiated conversation	Intervention		Usual care		P-value
		Adjusted proportion % (95% CI)		Adjusted proportion % (95% CI)		
**Physical Performance**	Oncologist	83%	(76%, 88%)	51%	(39%, 63%)	<.001
	Patient/caregiver	17%	(12%, 24%)	49%	(37%, 60%)	<.001
**Functional Status**	Oncologist	84%	(75%, 91%)	68%	(53%, 79%)	0.03
	Patient/caregiver	16%	(10%, 25%)	32%	(21%, 47%)	0.03
**Physical Performance or Functional Status**	Oncologist	84%	(77%, 89%)	58%	(45%, 70%)	<.001
	Patient/caregiver	42%	(30%, 55%)	16%	(11%, 23%)	<.001

Notes: Proportions generated using linear mixed models with practice site as a random effect

© 2022 Jensen-Battaglia M et al. *JAMA Network Open.*

eTable 4. Raw and Adjusted Proportions of Oncologist Response to Functional Status and Physical Performance Concerns

		Intervention		Usual care		P-value
GA Domain	**Oncologist Response**	**Adjusted proportion % (95% CI)**		**Adjusted proportion % (95% CI)**		
**Physical Performance**	Dismissed	1%	(0.60%, 3%)	4%	(2%, 8%)	0.106
	Acknowledged	55%	(49%, 61%)	48%	(39%, 57%)	0.213
	Appropriately addressed	50%	(39%, 62%)	18%%	(10%, 29%)	<.001
**Functional Status**	Dismissed	0.59%	(0.06%, 5%)	12%	(0.14%, 10%)	0.642
	Acknowledged	46%	(37%, 55%)	38%	(27%, 50%)	0.313
	Appropriately Addressed	23%	(14%, 34%)	13%	(7%, 25%)	0.189
**Physical Performance or Functional Status**	Dismissed	1%	(0.53%, 3%)	3%	(1%, 6%)	0.170
	Acknowledged	53%	(46%, 59%)	46%	(38%, 54%)	0.191
	Appropriately Addressed	43%	(33%, 53%)	17%	(10%, 26%)	<.001

Notes: Proportions adjusted for random effects of physician/cluster adjusted using linear mixed models with practice site as a random effect. Due to cluster adjustment and potential for oncologists to initiate a conversation which did not warrant further response, percentages do not sum to 100% within study arm.

© 2022 Jensen-Battaglia M et al. *JAMA Network Open.*

eTable 5. Raw and Adjusted Proportions of Recommendations Discussed to Address Functional Status and Physical Performance Concerns

	Intervention				Usual care				
Recommendation	**Raw N (%)**		**Adjusted proportion % (95% CI)**		**Raw N (%)**		**Adjusted proportion % (95% CI)**		**P-value***
**Referrals**	240	(35%)	24%	(18%, 30%)	14	(5%)	5%	(3%, 9%)	<.001
**Physical Exam**	4	(0.6%)	0.57%	(0.22%, 2%)	3	(1%)	1%	(0.36%, 3%)	0.387
**Treatment Modification**	7	(1%)	1%	(0.48%, 2%)	1	(0.4%)	0.37%	(0.05%, 3%)	0.348
**Information**	353	(51%)	22%	(14%, 34%)	10	(4%)	4%	(2%, 9%)	<.001
**Medication Review**	17	(2%)	**		0	(0.0%)	**		**

<u>Notes</u>: Proportions adjusted for random effects of physician/cluster adjusted using linear mixed models with practice site as a random effect.
**P-value reported for adjusted proportions*
***Outcome occurred 0 times in Usual care arm and therefore statistics were not possible*

© 2022 Jensen-Battaglia M et al. *JAMA Network Open.*
