# Supplementary material for: Association of Oncologist-Patient Communication With Functional Status and Physical Performance in Older Adults: A Secondary Analysis of a Cluster Randomized Clinical Trial
Source: JAMA Netw Open. 2022 Mar 18;5(3):e223039. doi: 10.1001/jamanetworkopen.2022.3039 (PMC8933739; doi:10.1001/jamanetworkopen.2022.3039)
Supplement: Supplement 2. — Trial Protocol [file jamanetwopen-e223039-s002.pdf]

Improving Communication for Cancer Treatment: Addressing Concerns of Older Cancer Patients and Caregivers

Protocol #: URCC 13070
URCC / University of Rochester NCORP Research Base
NCT02107443

Study Chairs: Supriya Mohile, MD, MS
James P. Wilmot Cancer Institute
URCC NCORP Research Base
601 Elmwood Avenue, Box 704
Rochester, NY 14642
(585) 275-5513

Co-Principal Investigators William Dale, MD, PhD
Arti Hurria, MD

Research Base Co-Chairs Gary Morrow, PhD, MS
Karen Mustian, PhD, MPH

Co-investigators Beverly Canin, Patient Advocate
Gary Morrow, PhD, MS
Karen Mustian, PhD, MPH
Marie Flannery, PhD, RN
Ronald Epstein, MD
David Dougherty, MD
Ekaterina (Katia) Noyes, PhD
Mohamed Tejani, MD
Robert Gramling, MD
Paul Duberstein, PhD
Arti Hurria, MD
William Dale, MD, PhD
Rita Gorawara-Bhat,PhD
Judith Hopkins, MD

Statistics Co-Chair Charles Heckler, PhD

Concept submitted to NCI 8/14/13
Concept resubmitted to NCI 9/27/13
Concept submitted to NCI 12/18/13
Concept approved 1/17/14
Protocol submitted to NCI 2/7/14
Protocol approved w/ comments 3/5/14
Amendment submitted to NCI 3/26/14
Amendment approved w/ comments 4/1/14
Amendment submitted to NCI 4/2/14
NCI approved: 4/8/14
Amendment submitted to NCI: 11/1/14
Amendment submitted to NCI: 4/25/17

PROTOCOL CONTACT INFORMATION

URCC NCORP Research Base:
URCC NCORP Research Base
Saunders Research Building
265 Crittenden Blvd
Box CU 420658
Rochester, NY 14642
phone: 585-275-5513
fax: 585-461-5601
website: http://extranet.urmc.rochester.edu/NCORP/

Principal Investigators:
Supriya Mohile, MD, MS
URCC NCORP Research Base
William Dale, MD, PhD
University of Chicago
Arti Hurria, MD
City of Hope Cancer Center

Co-Investigators:

Beverly Canin, Patient Advocate
Gary Morrow, PhD, MS
Karen Mustian, PhD, MPH
Marie Flannery, PhD, RN
URCC NCORP Research Base
Ronald Epstein, MD
David Dougherty, MD
Ekaterina (Katia) Noyes, PhD
Mohamed Tejani, MD
Robert Gramling, MD
Paul Duberstein, MD
University of Rochester

Rita Gorawara-Bhat, PhD
University of Chicago
Judith Hopkins, MD
SCCC-Upstate NCORP Community Site

Biostatistician:
Charles Heckler, PhD
URCC NCORP Research Base

Regulatory Contact:
Cathy Lesniewski
585-275-2282
cathleen_lesniewski@urmc.rochester.edu

Protocol Contact:
Libby Nagalski
585.275.1364
elizabeth_nagalski@urmc.rochester.edu

Program Managers:
Nikesha Gilmore, PhD
585-275-1275
Nikesha_gilmore@urmc.rochester.edu
Megan Wells, MPH
585-275-1277
Megan_wells@urmc.rochester.edu

Senior Program Manager
Sandy Plumb
585-275-1378
sandy_plumb@urmc.rochester.edu

Table of Contents

STUDY SCHEMA AND SUMMARY 4

1. BACKGROUND INFORMATION 5

2. STUDY OBJECTIVES 12

3. STUDY DESIGN 13

4. PARTICIPANT ELIGIBILITY 13

5. IDENTIFICATION, RECRUITMENT, AND CONSENT PROCEDURES 14

6. REGISTRATION 16

7. OUTCOMES 18

8. MEASURES TO BE COLLECTED 21

9. NCORP SITE RANDOMIZATION 23

10. INTERVENTION OVERVIEW FOR PHYSICIANS 24

11. OUTLINE OF STUDY-SPECIFIC PROCEDURES 25

11.1. Procedures Prior to Screening Visit 26
11.2. Screening: VISIT 0 26
11.3. Baseline: VISIT 1 27
11.4. Follow-up Visits 29
11.5. Telephone Team Call (1 to 7 days from Baseline) 29

12. REIMBURSEMENT 30

13. ADVERSE EVENT REPORTING 30

14. CRITERIA FOR WITHDRAWAL 31

15. STATISTICAL PLAN 32

16. DATA MANAGEMENT AND QUALITY ASSURANCE 35

17. DATA COLLECTION AND MEASURES 37

18. SUBJECT CONSENT AND PEER JUDGMENT 37

19. RECORD AND DATA RETENTION 37

20. REFERENCES 38

Appendix X-1: Measures

Appendix X-2: Study Related Forms

Appendix X-3: Patient Assessments

Appendix X-4: Clinical Research Associate Materials

Appendix X-5: Oncology Physician Assessments

Appendix X-6: Caregiver Assessments

Appendix X-7: Telephone Team Materials

Appendix X-8: Coding Procedures

STUDY SCHEMA AND SUMMARY

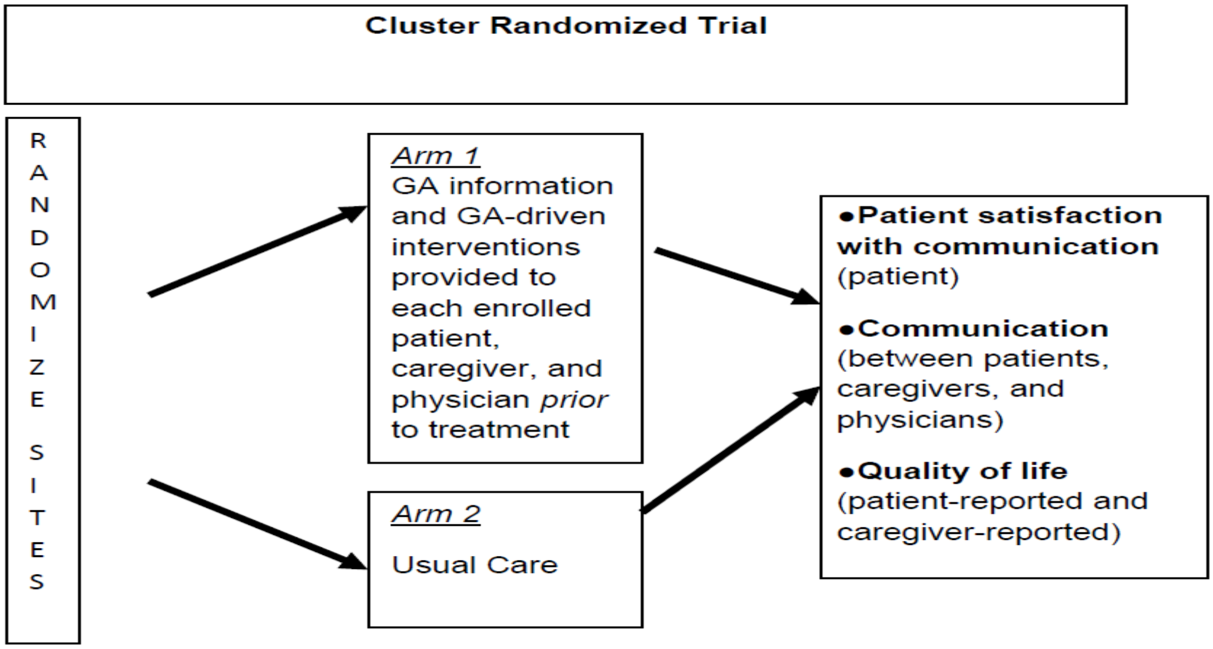

This study will evaluate whether a standardized geriatric assessment (GA)[i] administered through a novel web-based approach can facilitate communication of age-related problems that could influence outcomes important to the older cancer patient and his/her caregivers.[ii] Adults, age $\geq$ 70 with an advanced solid tumor malignancy in the University of Rochester Cancer Center NCI Community Oncology Program (URCC NCORP) network will be eligible. Oncology physicians who practice at sites within the URCC NCORP network are eligible to participate in the study and will be enrolled. Their eligible patients will then undergo the informed consent process; those patients who agree to participate in this study will undergo a clinical assessment consisting of sociodemographic characteristics and GA. Eligible patients should choose one caregiver to participate.

NCORP practice sites with IRB approval of the protocol will be randomized to receipt of GA plus GA-driven recommendations (Arm 1) or usual care (Arm 2). A NCORP practice site will be defined as any practice location within an overarching NCORP designation where oncology physicians and study staff work independently (e.g., do not cross over into another practice site). In Arm 1, oncology physicians, patients, and caregivers will be provided with GA summary plus targeted recommendations (i.e., GA-driven recommendations). In Arm 2, patients will complete GA, but no GA summary or GA-driven recommendations will be provided to the oncology teams except for information regarding clinically significant cognitive impairment and/or depression. A brief follow-up GA will be collected at 4-6 weeks, 3 months, and 6 months after baseline registration.

In both arms, the first office visit with the oncology physician after GA will be audio-recorded (to measure number of concerns brought up by patients and caregivers and whether they were addressed by their oncology physicians), and measures of quality of life and satisfaction will be collected at 4-6 weeks, 3 months, and 6 months after baseline registration. The primary outcome, patient satisfaction regarding communication about age-related issues as measured by a modified Health Care Climate Questionnaire (HCCQ), will be obtained via a phone call

---

[i] Geriatric assessment (GA) is a compilation of survey-based and assessment tools to assess geriatric domains such as comorbidity, functional status, nutrition, physical performance, cognition, and social support, which measures aging related issues that can affect the quality of life of an older patient with cancer. GA can better predict tolerance to cancer treatment and adds important age-related information that is not routinely captured by oncologists.

[ii] For purposes of this study, a caregiver is defined as a valued and trusted person in a patient's life who is supportive in health care matters by providing valuable social support and/or direct assistive care. The caregiver accompanies the patient to medical appointments, is able to listen and give thoughtful advice and may be a family member, partner, friend, or professional caregiver.

administered by trained personnel, the Telephone Team, who are blinded to group assignment, within 1 to 7 days of the baseline audio-recorded clinic consultation, hereby referred to as the Telephone Team Call. If a telephone call is not feasible, the HCCQ will be mailed with a return envelope provided. Caregivers will complete measures of satisfaction, and caregiver burden (both health and economic) at the same time points. However, caregivers will not receive the Telephone Team Call.

A total of 528 patients and 528 caregivers (maximum) will be enrolled in the study.

The acronym for this study is COACH, which stands for Communication On Aging and Cancer Health.

1. BACKGROUND INFORMATION

1.1. Intervention to be Studied

The URCC NCORP Research Base will conduct a cluster randomized study evaluating whether providing a GA summary with targeted recommendations (i.e., GA-driven recommendations) to older patients with advanced cancer, their caregivers, and their oncology physicians can improve patient satisfaction with communication about age-related issues and concerns. Secondary aims will determine if the intervention increases discussions about age-related issues during a clinic consultation, improves patient-reported quality of life, and improves caregiver burden and caregiver satisfaction with communication.[1,2]

1.2. Background and Significance

Older adults with cancer and their caregivers are presented with complex information regarding the risks and benefits of treatment for advanced cancer, but age-related concerns and outcomes are not usually discussed.[3] Outcomes important to the older adult with cancer include not only tumor shrinkage and progression-free survival (which are traditionally measured in clinical trials), but also the effect of treatment on health-related quality of life (HRQoL) and geriatric domains.[3] Our preliminary data with older patients with advanced cancer and their caregivers demonstrates that the vast majority want information on how cancer treatment can affect geriatric domains (e.g., independence, mood, cognition). Our preliminary data has also shown that incorporating GA, a validated patient-centered approach to the assessment of underlying health status, into the clinical decision making process for older patients with cancer is feasible and helps to identify conditions (normally overlooked in routine oncology care) that are rated as very important to older patients and caregivers.[4-7] Common assessment instruments in oncology such as performance status do not address critical domains that affect patient-centered outcomes, morbidity, and mortality in the older patient.[8] Significant gaps in knowledge regarding cancer treatment in older and/or vulnerable adults led to the formation of the Cancer and Aging Research Group (CARG),[9] a coalition of geriatric oncology researchers and older patient advocates, working together towards improving clinical care for older adults with cancer. In 2010, CARG received a U13 grant (U13 AG038151), in collaboration with the NCI and NIA, to conduct and disseminate findings from 3 conferences over 5 years on "Geriatric Oncology Research to Improve Clinical Care."[3,8,10] The first U13 conference identified the need to incorporate GA into clinical care to facilitate communication regarding the risks and benefits of cancer treatment for older patients with cancer plus other age-related health status conditions.[8]

1.3. Condition to be Studied

A growing population of older patients is at high risk for adverse outcomes from cancer treatment. Cancer is a disease of aging; approximately 60% of all cancers and 70% of cancer mortality occur in persons aged 65 years and over.[11] The number of cancer patients over the age of 65 is projected to significantly increase over the next 20 years (see Figure 1).[12] Aging is a highly individualized process, characterized by an increased prevalence of health status conditions that can affect decision making for cancer treatment, treatment tolerance, and ultimately outcomes.[3,13,14] The PI has shown that older adults

Figure 1: Increase in older patients with cancer

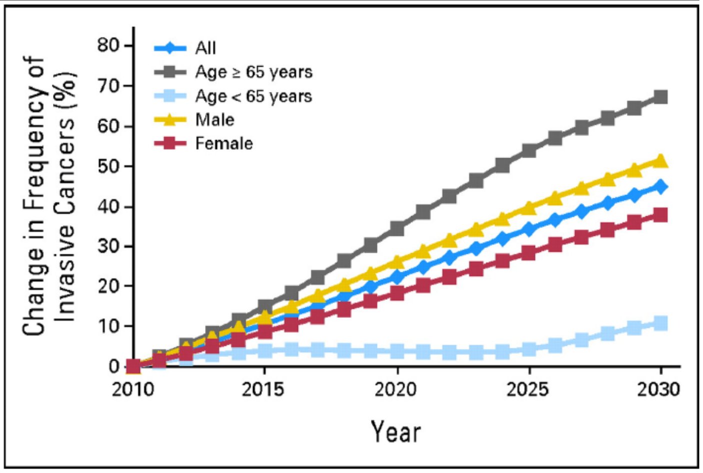

Pal S K , Hurria A JCO 2010;28:4086-4093

with cancer have a high prevalence of comorbidity, disability, and geriatric syndromes.[15,16] The majority of older patients with cancer are treated based on extrapolations of evidence derived from clinical trials providing data on the safety and efficacy of treatment in younger adults or in older patients who are fit without other health status conditions.[17]

1.4. Geriatric Assessment (GA)

GA is a validated mechanism to obtain patient-reported information about issues important to the older adult including function (i.e., ability to remain independent in self-care and community), psychological status, cognitive abilities, social support, and the impact of medical problems on quality of life. In our team's research, geriatric domains captured on GA are the most important outcomes for older patients with cancer, more so than traditional oncology outcomes (such as tumor response, progression-free survival). The results from our pilot work with patients and caregivers (see Preliminary Data, Table 1) are consistent with another study that demonstrated that older patients may forgo life-sustaining measures if they will have a significant impact on function and cognition.[18]

1.5. Geriatric Assessment Components and Relevance to Older Patients with Cancer

GA provides valuable information that could identify and help address the concerns of older patients with cancer and their caregivers. A description of each GA domain and its relevance to the older patient with cancer is provided below.

1.5.1. Functional Status and Physical Performance: The need for functional assistance (measured by ability to complete activities of daily living) is predictive of chemotherapy toxicity and survival.[19-22] Physical performance measures objectively evaluate mobility and fall risk.[23,24] Falls are common in cancer patients and predictive of adverse outcomes.[1,16,25]

1.5.2. Comorbidity and Polypharmacy: Among patients with cancer, comorbidity is associated with poorer overall survival.[26-30] Comorbidity impacts cancer treatment tolerance.[31-34] Furthermore, these comorbid conditions may predispose patients to the risks of polypharmacy and drug interactions.[35]

1.5.3. Nutrition: Poor nutritional status is associated with an increased need for functional assistance and poorer overall survival in the geriatric population.[36] Unintentional weight loss during the 6 months prior to chemotherapy is associated with lower chemotherapy response rates and lower overall survival.[37]

1.5.4. **Cognition:** A cognitive assessment is needed to determine if the patient has the decisional capacity to consent and adhere to supportive care medication instructions and understand the indications to seek attention. In the presence of cognitive impairment, the involvement of the patient's family or caregiver is required to maintain safety.[38-41]

1.5.5. **Psychological State and Social Support**: In a study of older adults with cancer, significant distress was identified in 41% of older adults, and poorer physical function correlated with higher distress.[41] In both the geriatric and oncology literature, social isolation has been linked to an increased risk of mortality.[42-44]

The evidence suggests that although underlying health status issues and deficits in geriatric domains correlate directly with toxicity of therapy and patient-centered outcomes, these considerations are not addressed in routine oncology clinical care.[3,8] Although the commonly used Karnofsky Performance Status (KPS) and Eastern Cooperative Oncology Group (ECOG) performance status (PS) measures do correlate with treatment toxicity, these tools were validated in younger groups of patients and do not reliably predict outcomes in older adults with cancer.[45-47] GA, a compilation of validated tools to assess geriatric domains such as comorbidity, functional status, nutrition, physical performance, cognition, and social support, can help define the "stage of aging."[48] GA can better predict tolerance to treatment[2,49,50] and adds important age-related information that are not captured by traditional PS assessment tools utilized in oncology.[51] A CARG study (Hurria and Mohile, et al.) found that several GA variables predicted severe chemotherapy toxicities in older patients.[1] GA has also shown to predict overall survival in older cancer patients.[52] Studies have found that oncology physicians will modify treatment decisions based on GA results when information is provided to them.[5,53] Our research team has found the GA proposed in this study is feasible in oncology clinics and trials.[1,7,54] Unfortunately, clinical trial data that dictate evidence-based care for patients with cancer, the majority of whom are older with additional health status considerations, have not

generally included GA. Successful completion of this research has the potential to incorporate communication of GA results into the clinical care of older adults with advanced cancer.

1.6. Gap in Knowledge

There is a critical gap in knowledge regarding how to improve communication about age-related concerns between older adults with cancer, their caregivers, and oncology physicians.[54-56] The use of health-related quality of life (HRQoL) assessments in clinical practice has been shown to monitor disease and treatment, improve the delivery of care, and detect physical or psychosocial problems that otherwise might be overlooked.[57,58] For example, Detmar et al. showed that providing physicians and patients with summaries of patient-reported HRQoL information increased discussions and improved management of HRQoL issues in patients with cancer undergoing chemotherapy. Similarly, important patient-reported information obtained from GA could help oncology physicians address age-related concerns of patients and their caregivers thereby improving satisfaction with communication and HRQoL. Despite the fact that the majority of cancer patients are aged 70 and over, most oncology physicians have received little training in the care of older patients.[9] As a result, common problems facing an aging population of cancer patients may go unrecognized and produce serious consequences.[10,55] Identification of age-related concerns may also facilitate discussions about prognosis, which is important because many patients do not understand that cancer treatment is not curative in the setting of advanced cancer and can negatively affect quality of life.[59] Although GA predicts risk from cancer treatment and survival in older patients with cancer, there is no evidence-based approach regarding the use of GA to improve communication during the decision making process for cancer treatment. The hypothesis of this research proposal is that providing older patients with cancer, their caregivers, and oncology physicians with a summary of GA information and recommendations can improve patient satisfaction with communication about age-related health concerns, improve observed communication of age-related concerns captured with audio-recordings of clinic encounters of older patients, caregivers, and oncology physicians, and improve patient-reported quality of life.

1.7. Geriatric Assessment-Driven Recommendations and Relevance to Older Patients with Cancer and Caregivers

We hypothesize that providing information on GA and recommendations to oncology physicians can improve patient-reported outcomes such as HRQoL, which is similar to an approach showing that an early palliative care intervention improved outcomes of patients with advanced lung cancer.[60] Interventions guided by GA have positive effects on health outcomes including prevention of disability and reduction in the risk of falls, unplanned hospitalizations, and nursing home admissions.[3,61-63] Several studies have shown that the implementation of GA-driven recommendations into the clinical care of older patients with cancer is feasible.[4-7] The ELCAPA study illustrated that providing GA information to oncology teams can influence treatment decisions, although outcomes from these interventions were not measured in this study.[5] Another pilot study showed that GA affected the oncology treatment plan.[53] In a study by McCorkle et al.,[64] geriatric nurse practitioners conducted GA with cancer patients, which led to a survival advantage (67% in the intervention group compared with 40% in the control group) and improved HRQoL. In a study by Goodwin et al., breast cancer patients in the GA-driven recommendations group were significantly more likely to return to normal functioning than controls.[65] Different approaches for chemotherapy selection and dosing for older and/or frail patients are supported by the literature and are incorporated into the framework as GA-driven recommendations. For example, the FOCUS-2 trial found that chemotherapy for advanced colorectal cancer was safe and efficacious in the older and/or frail patient if started at a 20% dose reduction with escalation as tolerated.[66] The GA and recommendations utilized in this proposal have been developed through preliminary work, extensive review of the evidence, and clinical expertise of the geriatric oncology physicians on the research team.

1.8. Preliminary Data

The investigative team is poised to build upon a considerable body of prior work. The research team has conducted studies that have demonstrated the high prevalence of health status issues that could influence cancer outcomes in older patients.[15,16] They have developed a GA tool for older persons with cancer and the feasibility of this tool has been studied in hundreds of cancer patients in multicenter clinical trials.[1,7,54] They have collaborated on a prospective multicenter study to quantify the risks of chemotherapy among older adults with cancer.[1] Dr. Mohile has pilot-tested GA and GA-driven interventions with 200 patients from her geriatric oncology clinic.[71] Drs. Mohile, Epstein, and Dale have experience in the study of communication in oncology. Other investigators

lend significant interdisciplinary expertise and stakeholders (CARG, an advisory board of older patients with cancer, advocates and caregivers, and community oncology physicians) have provided significant input at all stages of preliminary work.

Of importance, Dr. Ron Epstein and Dr. Mohile collaborate on a NCI-funded study (R01 NIH/1R01CA140419-01A1), which evaluates whether a communication coaching intervention that targets oncology physicians, patients and caregivers improves discussions regarding prognosis and treatment choices in advanced cancer. The primary outcome for this study (physician communication behaviors) was obtained from clinic consultations that are audio-recorded. The patients (n=81 older patients) also completed a measure of patient satisfaction (Health Care Climate Questionnaire), which provided preliminary data for sample size calculations for the current proposal as the primary outcome.

1.8.1. Prevalence of Health Status Issues in Older Patients with Cancer. Using a nationally representative population-based database, Mohile and collaborators published two investigations that demonstrated that disability, comorbidity, and geriatric syndromes are more common in cancer patients than in those without cancer and that cancer was independently associated with having these conditions.[15,16] In addition, Drs. Hurria and Mohile have collected GA data from over 500 older cancer patients receiving chemotherapy at 7 institutions.[1] *The assessment revealed a number of findings that would not have been detected from a visit's routine history and physical exam.* For example, 41% of patients needed assistance with instrumental activities of daily living despite a mean physician-reported KPS of 85 out of 100; 16% had recent falls, and 6% had severe cognitive impairment on the cognitive screening test.

1.8.2. Feasibility of a Geriatric Assessment for Older Adults with Cancer.[7,54] The geriatric and oncology literature was reviewed to choose validated GA measures. Selection criteria included reliability, validity, brevity, the ability to self-administer, and the ability to prognosticate risk for morbidity or mortality in an older patient.[7] The final selection of measures was approved by the Cancer and Leukemia Group B (CALGB) Cancer in the Elderly and Quality of Life Committees. The initial feasibility study of this tool was conducted in a multicenter study by Dr. Hurria and Dr. Mohile. Forty patients (mean age 74, range 65 to 87) with cancer participated in the study. The GA was feasible, as demonstrated by a mean time to completion of 27 minutes; 90% of patients were satisfied with the questionnaire length, and 78% were able to complete it on their own.[7] Subsequently, CALGB 360401 evaluated the feasibility of incorporating the GA into oncology cooperative group trials for older adults (n=93).[54] The GA results met the protocol-specified feasibility criteria for use in the cooperative group.

1.8.3. Can the Geriatric Assessment Predict Chemotherapy Toxicity?[1] The primary objective of Drs. Hurria and Mohile's previous study was to determine if GA measures predicted grade 3-5 toxicity (severe) using the NCI Common Terminology Criteria for Adverse Events (CTCAE, V3.0). Among the 500 enrollees, the mean age was 73 years (range 65-91); 61% had metastatic disease and 71% received 1st line chemotherapy. Grade 3-5 toxicity occurred in 53% (50% grade 3, 12% grade 4, 2% grade 5). Risk factors for severe toxicity included: 1) age ≥ 73, 2) cancer type (GI or GU), 3) standard dose, 4) poly-chemotherapy and the following GA measures, 5) falls in the last 6 months, 6) assistance with instrumental activities of daily living, and 7) decreased social activity.

1.8.4. Feasibility of Intervention Proposed in the Study, Contribution of Stakeholders, and Impact on Clinical Care.[71] Dr. Mohile directs a referral-based consultative Specialized Oncology Care in the Elderly (SOCARE) clinic which has collected pilot data on patient preferences, outcomes, and GA-driven interventions in over 200 older patients with cancer (Table 1).[71] All measures and the GA intervention in this proposal are utilized within the SOCARE clinic. Patients were referred to the clinic by their primary oncology team. As routine in the clinic, each patient completes GA by paper and pencil, and summaries of results are provided to the patient and his/her caregivers. Ratings of each GA domain and acceptability/understanding of the GA summaries are assessed. GA-driven recommendations, developed by the SOCARE team, are provided to the referring oncology physician, the patient, and his/her caregivers. *Patient characteristics:* Mean age was 82.1 (70-95) and 75% had advanced disease. GA revealed 68% with functional impairment; 70% had ≥3 significant comorbidities; 39% had poor nutrition;[72] 26% screened positive for depression;[73] 59% reported

inadequate social support; 20% had an abnormal cognition screen (but had decision making capacity);[74] 34% had recently fallen, and 60% had poor physical performance.[23,75] Acceptability of summaries: We have developed and revised the GA summaries based on extensive qualitative and written feedback from the patients and their caregivers. The summaries include a description of the test used to assess the domain in lay language, an interpretation of the score for each domain, and a list of interventions to consider if the score signifies impairment. Acceptability of GA-driven recommendations: On average, 80% of the GA-driven recommendations were implemented by the referring oncology physician with an average of 6 interventions per patient (range 3-15).

1.8.5. The research team has experience with the assessment of patient-physician communication (using audio-recordings, surveys, and chart reviews) in the setting of advanced cancer. Dr. Epstein, an expert in patient-centered communication and co-investigator on this study, has conducted multi-method research to study patient-physician interactions using analyses of patient and physician surveys and medical record audits.[76] His research team has helped to establish that patient-centered communication is associated with improved information exchange, reduced symptom burden, lower health-care costs, and greater patient involvement in decision making.[77-79] The measures to assess decision making in Dr. Epstein's NCI-funded RO1 (Dr. Mohile is a co-investigator) have been adapted for patients with advanced cancer.[67,68]
Using two trained coders, we examined the audio-recorded interactions between the older patients (n=32) with advanced cancer and their oncology physicians (at 8 different sites) to assess number of discussions related to geriatric domains brought up by patient and/or caregiver that were addressed by the oncology physician and the number of "missed opportunities" (i.e., concerns brought up by patients and caregivers that were not adequately addressed by the oncology physician). We found that at baseline, there were 26 discussions and 26 missed opportunities among 32 audio-recorded visits. Fifty percent of visits had 0 age-related discussions; the median was 1 (range 1-3). The majority of missed opportunities were related to functional ability (e.g., not taking medicines correctly), physical functioning (e.g., inability to climb stairs, falls), social support (e.g., difficulty getting to appointments), and medications (e.g., polypharmacy, drug interactions). This study was not elderly specific and did not incorporate GA (which can identify deficits as described above), but the results provide an estimate of the baseline number of discussions about age-related issues that occur in usual care within oncology. We were able to utilize this data to help gather usual care information to inform our analytic plan.

Dr. Dale, Chief of Geriatrics and Palliative Care at the University of Chicago, has expertise in medical decision making, quality of life, and frailty and has studied the role of emotions in decisions about screening, diagnosis, and treatment of cancer in older persons. He and Dr. Mohile have collaborated on a study that evaluated patient-physician decisions with regard to the treatment of advanced prostate cancer.[80] His team, including Rita Gorawara-Bhat, a senior staff scientist with expertise in qualitative methods (in-depth interviewing, cognitive interviewing, focus groups, Delphi techniques, etc.) and survey methods (questionnaire design), has significant expertise in the administration and collection of patient-reported outcomes via telephone. The University of Chicago team, which is funded through a PCORI subcontract, will be blinded to group assignment and will be able to administer and collect the primary aim as the Telephone Team.

1.9. Conceptual Model Highlighting Relevance of Topic to Older Patients and Caregivers.
In 2007, the NCI published a monograph, co-authored by Drs. Epstein and Street, *Patient-Centered Communication in Cancer Care,* which reviewed 2200 relevant communication studies.[67] **The monograph includes a conceptual framework that has been adapted for this study (Figure 2).**

1.9.1. Patient-centered care. Patient-centered care considers the patient's unique experience of illness ***as equal in importance*** to the physician's disease/diagnostic perspective; it directs clinicians to see the world ***both*** through the patient's eyes as well as through a clinical lens.[68,69] There is general agreement that patient-centered care: 1) explores and validates patients' individual experiences of suffering and their needs, expectations and values; 2) offers patients opportunities to provide input into and participate in their care; and 3) enhances partnership, shared understanding, and trust in the patient-physician relationship.

1.9.2. Communication is a process of mutual influence and interaction. The proposed study is based on the Street/Epstein "Ecological Model" of patient-centered communication.[70] It is an *interactional model*. In health-care settings, effective communication is characterized by:

1.9.2a. Informed, activated, participatory, and communicative patients and caregivers. Activated patients and caregivers *express* their concerns and *participate* actively in decisions. Providing GA information to patients and their caregivers could facilitate "activation." We will measure participation in the encounter through direct observation and measure the quality of the patient-clinician relationship using surveys.

1.9.2b. Informed, receptive, patient-centered, and communicative clinicians. Clinician patient-centered behaviors include *organizing the visit* to *elicit* patients' concerns, *raising issues* often ignored in clinical consultations, and *responding* to patients' concerns with information and empathy. We can observe the degree to which oncology physicians explore and validate patients' and caregivers' concerns about age-related conditions. Patients and caregivers will report on the degree to which they felt included in discussions regarding their care.

1.9.2c. A health-care system that provides accessible, well-organized, and responsive health services that are tailored to the patient's needs. For example, greater access to services that address age-related issues may improve outcomes. We will measure this dimension via patient/caregiver surveys.

Figure 2: Conceptual Model

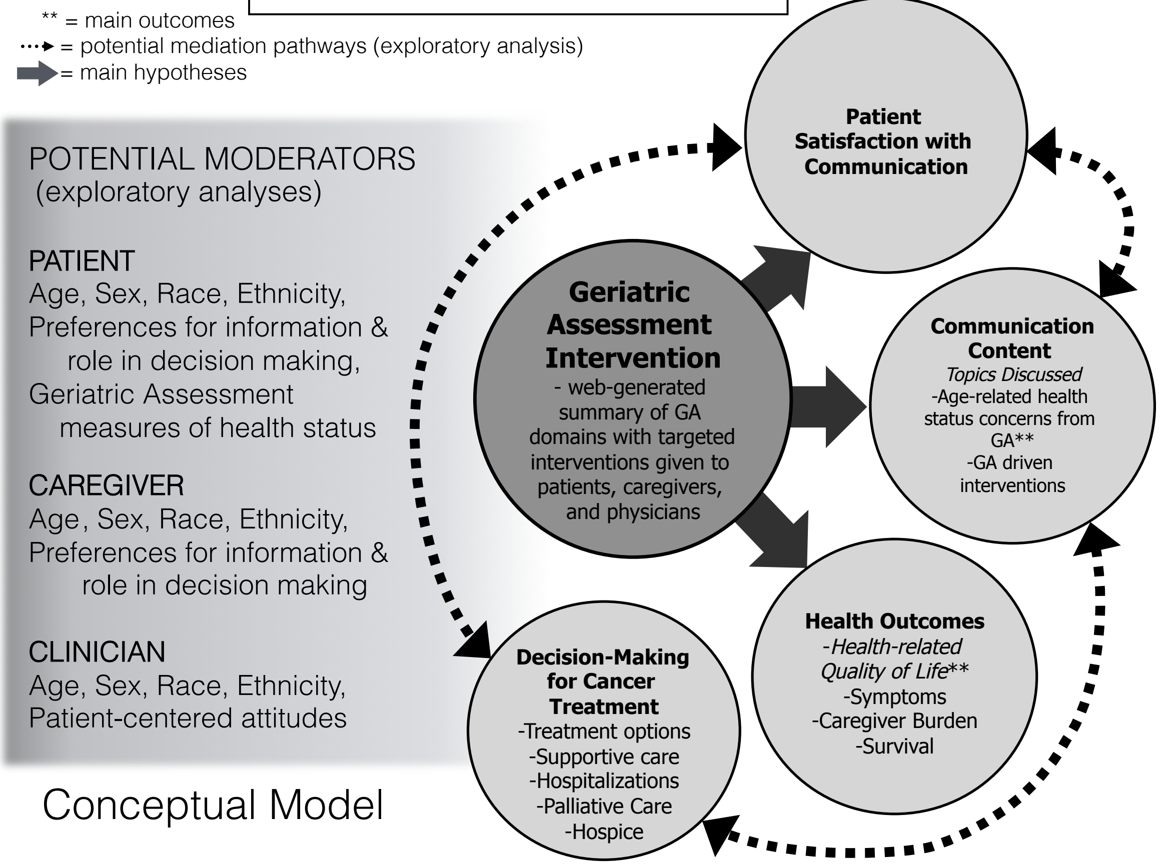

1.10. Study Subjects

The study involves adult human subjects.

NCORP practice sites will be randomized within a 2-arm cluster randomized design utilizing NCORP practice sites as the unit of randomization. A NCORP practice site will be defined as any practice location within an overarching NCORP designation where oncology physicians and study staff work independently (i.e., do not cross over into another practice site).

Study subjects will include:

• ***Oncology physicians*** at NCORP practice sites

• ***Patients*** will complete surveys and will also agree to have a clinical consultation with an oncology physician audio-recorded (baseline visit). Patients will have advanced cancer and various levels of functional status. Patients will be asked to identify a caregiver who may choose to participate in the study. As much as possible, caregivers should be recruited to be part of the study when the patient is recruited. In the rare circumstance that there is no willing caregiver to participate, the patient will be allowed to participate without a caregiver.

• ***Caregivers*** must enroll in the study before or on the baseline visit because they will accompany the patients during the clinic consultation that will be audio-recorded (baseline) and complete surveys.

2. STUDY OBJECTIVES

This is a cluster randomized study within the URCC NCORP network evaluating whether GA summary plus GA-driven recommendations can improve patient satisfaction with communication regarding age-related issues between patients, oncology physicians, and caregivers. The study has received support from the Patient Centered Outcomes Research Institute (PCORI) under their "Communication and Dissemination" portfolio. In addition, the National Cancer Institute (NCI) review was required which led to changes in the aims and statistical plan. The NCI review led to a new primary aim (satisfaction with communication regarding age-related issues) and the original PCORI primary aim (discussions about age-related issues during clinic consultation) became Secondary Aim 1.

2.1. Primary Aim (primary aim as specified by the NCI): To determine if providing geriatric assessment (GA) summary plus GA-driven recommendations to patients, their caregivers and oncology physicians **improves patient satisfaction with communication with the oncology physician** regarding age-related concerns.

Primary hypothesis: Patient satisfaction with communication with the oncology physician about age-related issues will be significantly higher in the intervention group compared to the control group.

The NCI primary outcome, patient satisfaction regarding communication about age-related issues as measured by a modified Health Care Climate Questionnaire (HCCQ-age), will be obtained via the Telephone Team Call, a phone call administered by trained personnel blinded to group assignment within 1 to 7 days of the baseline audio-recorded clinic consultation. The HCCQ-age will be mailed (with a return envelope) if a telephone call is not feasible. If the responses to the survey are not able to be obtained before the 4-6 week assessment, the HCCQ-age at 4-6 weeks will be utilized.

2.2. Secondary Aims

2.2.1. Secondary Aim 1 (primary aim as specified by PCORI): To determine if providing GA summary plus GA-driven recommendations to patients, their caregivers, and oncology physicians increases **discussions** about age-related issues during clinic consultation.

Hypothesis: A higher proportion of age-related issues will be discussed and addressed in the intervention group.

The outcome measure for this aim is the number of age-related discussions related to the different aspects of geriatric assessment that occur in the consultation clinic visit between the patient, oncology physician, and caregiver. The clinic visit will be audio-recorded. This outcome was accepted as the primary aim by PCORI.

2.2.2. Secondary Aim 2: To determine whether initially providing patients, their caregivers, and oncology physicians with GA summary plus GA-driven recommendations prior to their treatment influences **quality of life** of older patients receiving treatment and their caregivers.

2.2.3. Secondary Aim 3: To determine whether providing patients, their caregivers, and oncology physicians with GA summary plus GA-driven recommendations influences **caregiver satisfaction with communication** about age-related issues.

2.3. Exploratory Aims:
Exploratory aims will evaluate (1) whether the number of discussions about age-related issues during the clinic visit correlates with patient satisfaction, (2) whether the intervention increases the proportion of age-related concerns that are acknowledged and addressed, and (3) if communication about age-related issues influences how patients, caregivers, and oncology physicians make decisions for cancer treatment. An additional exploratory aim will examine the impact of the intervention on survival.

3. STUDY DESIGN

See Study Schema and Design for summary.

3.1. Choice of comparators

Because GA is not performed by community oncology physicians and this study ultimately will allow patients/caregivers/oncology physicians to choose their cancer treatments, a usual care comparator arm is appropriate and will allow for the accurate and appropriate assessment of how the intervention can improve communication about age-related issues and outcomes compared to current clinical practice. This study design is similar to previous studies that evaluated the impact of providing summarized HRQoL information to patients and oncology physicians on communication and outcomes. Usual care was the comparator arm in these cluster randomized studies.[57,81]

3.2. Choice of study design

The study is designed as a cluster randomized trial because a care of service model is applied to each patient by the oncology team. If a cluster randomized design were not undertaken, there would be contamination in that oncology physicians could choose the care of service model if they were exposed to patients randomized to both arms. Given rapid changes that can occur in oncology practice with new supportive care and treatment agents, it is important to compare outcomes in the same time frame as would be possible in a cluster randomized study design compared to a "pre" versus "post" intervention study design.

4. PARTICIPANT ELIGIBILITY

4.1. Entry Criteria for Oncology Physicians

Oncology physicians must work at a NCORP practice site with no plans to leave that NCORP practice or retire at the time of enrollment into the study.

4.2. Entry Criteria for Patients

4.2.1. Inclusion Criteria for Patients

4.2.1a. Male or female 70 years of age or older

4.2.1b. Diagnosis of an advanced solid tumor malignancy (advanced cancer) or lymphoma. In most situations, this would be a stage IV cancer. A patient with a diagnosis of stage III cancer or lymphoma is eligible if cure is not possible or anticipated. Clinical staging without pathological confirmation of advanced disease is allowed.

Must be considering or currently receiving any kind of cancer treatment (any line), including but not limited to hormonal treatment, chemotherapy, monoclonal antibody therapy, or targeted therapy. Patients who are considering therapy are eligible even if they ultimately choose not to be on therapy. Patients with a history of any previous cancer treatment, including radiation and/or surgery are eligible. A patient may also be enrolled on a treatment trial and participate in this study, if all other inclusion and exclusion criteria are met.

4.2.1c. Have at least one geriatric assessment domain meet the cut-off score for impairment other than polypharmacy per Table 2.

4.2.1d. Have visits planned with the oncology physician for at least 3 months and be willing to come in for study visits.

4.2.1e. Able to provide informed consent or, if the oncology physician determines the patient to not have decision-making capacity, a patient-designated health care proxy (per institutional policies) must sign consent by the baseline visit.

4.2.1f. Subject has adequate understanding of the English language because not all GA measures have been validated in other languages.

4.2.2. Exclusion Criteria for Patients

4.2.2a. Have surgery *planned* within 3 months of consent. Patients who have previously received surgery are eligible.

4.2.2b. Have already made a decision to not undergo any cancer treatment (e.g., being followed in best supportive care or hospice).

4.3. Entry Criteria for Caregivers

A caregiver can be anyone, age 21 or over, who is able to understand spoken English and understand the study process and provide informed consent. One caregiver for each patient will be eligible and must be chosen by the patient. For the purposes of this study, a caregiver is defined as a valued and trusted person in a patient's life who is supportive in health care matters by providing valuable social support and/or direct assistive care. The caregiver accompanies the patient to medical appointments, is able to listen and give thoughtful advice and may be a family member, partner, friend, or professional caregiver.

4.3.1. Inclusion Criteria for Caregivers

4.3.1a. Selected by the patient when asked if there is a "*family member, partner, friend or caregiver [age 21 or older] with whom you discuss or who can be helpful in health-related matters;"* patients who cannot identify such a person ("caregiver") *can* be eligible for the study. A caregiver need not be someone who lives with the patient or provides direct hands-on care. A caregiver can be any person who provides support (in any way) to the patient.

4.3.1b. If a health care proxy signs consent for or with a patient, and wants to participate in the caregiver portion of the study, this same person will always be the caregiver selected. If a health care proxy does not want to enroll as a caregiver in the study or, if enrolled, chooses to stop their own participation in the caregiver portion of the study, but is able to assist the patient in completing the study, the patient can still participate. In other words, the health care proxy can choose NOT to participate in the caregiver portion of the study. This does not preclude the patient from participating in the patient portion of the study with the health care proxy's assistance.

4.3.2. Exclusion Criteria for Caregivers

4.3.2a. Caregivers unable to understand the consent form due to cognitive, health, or sensory impairment will be excluded.

5. IDENTIFICATION, RECRUITMENT, AND CONSENT PROCEDURES

Patients and their caregivers will be recruited from the outpatient community oncology practices affiliated with the URCC NCORP Research Base network. The results of this study will be generalizable to the majority of older adults with cancer and their caregivers because it will include older cancer patients and their caregivers from diverse backgrounds and at varying health statuses.

5.1. Study Participants (Patients and Caregivers)

Study participants will be identified by their treating oncology physicians, who enroll in the study, the nurses that work with the enrolled oncology physicians, and the clinical research associates (CRAs). The CRAs will work closely with the enrolled oncology physicians and nurses to identify patients aged 70 and over with advanced cancer. The oncology physician then confirms if the patient is an eligible study candidate for all requirements other than GA impairment by completing the eligibility checklist with the CRA and signing it. The oncology physician or CRA will introduce the study to the eligible patients and/or their designated health care proxies/caregivers, and will provide them with an IRB approved study brochure and consent to review. Adequate time will be provided to the patient and/or the designated health care proxy to read the consent. By this time, the patient should have selected a possible caregiver. The CRA, the oncology physician, and the nurses are available to answer any questions the patient and/or caregiver/healthcare proxy may have about any aspect of the study prior to consenting and throughout the entire study period. Patients and/or their designated health care proxies and caregivers may choose to sign the appropriate (patient or caregiver) informed consent documents immediately on the day the study information is presented to them or they may choose to take the consent forms home and discuss them with others; then if they decide to participate in the study, they can provide signed consent forms the next time they meet with the CRA or oncology physician.

5.1.1. Informed consent will be obtained from the patient, unless they lack capacity to provide consent. If a patient lacks capacity, a health care proxy will be required to sign consent per institutional or local policies on consent for incapacitated/decisionally impaired subjects. If the patient does not have an appointed health care proxy on or before the baseline visit, he/she will not be enrolled onto the study. All consent documents will be signed by the patient and/or designated health care proxy and maintained in the patient record with copies provided to the patient and/or designated health care proxy.

5.1.2. The screening measures will then be performed. Those with a diagnosis of dementia, as noted in their medical record or diagnosed by a physician, or who meet the cut-off score for impairment on the cognitive screen (score of 11 or more on Blessed Orientation-Memory-Concentration Scale) can be included if a designated health care proxy selected by the subject signs the consent. The goal of the intervention is to improve outcomes of older cancer patients with all underlying health conditions including cognitive issues. Therefore we will include these patients and will conduct the assessments with assistance of the proxy. The health care proxy should agree to participate in the study as the caregiver. If a health care proxy chooses to stop their own participation in the study, but is able to assist the patient in completing the study, the patient can still participate.

5.1.3. Ethical standards for human subjects will be strictly followed in accordance with local policies and/or institutional review board requirements on the enrollment of adult decisionally incapacitated research subjects and permission of authorized representatives.

5.1.4. Current, state, federal, and institutional regulations concerning informed consent will be followed. Participation in this study is voluntary. Participants are free not to take part or to withdraw at any time, for whatever reason, without risking loss of present or future care they would otherwise expect to receive. In the event that a patient does withdraw from the study, the information they have already provided will be kept in a confidential manner. Data will be used unless permission is revoked in writing and sent to their oncology physician or the URCC study team. Site oncology physicians will be directed to forward any such correspondence to the URCC Research Base.

5.2. Oncology Physician Recruitment

Because oncology physicians are being recruited and enrolled from sites across the country by the URCC Research Base , oncology physicians will read and agree to participate either via Research Electronic Data Capture (REDCap) or on paper if REDCap is not a feasible option. REDCap is a software toolset (see Section 16.3 for more information) for electronic collection and management of research and clinical trial data developed by Vanderbilt University. Oncology physicians will be enrolled prior to enrolling (screening registration) their first eligible patient. Staff from the URCC Research Base (including the PI) will be available to answer any questions the oncology physicians may have over the phone. Procedures for the oncology physicians are minimal risk and involve: completing surveys that will be de-identified, and identifying patients for the study.

Oncology physicians will provide baseline demographic information, fill out a baseline survey that evaluates preferences for decision-making, and help identify their own patients who may be eligible for the study. The baseline survey can be completed online via REDCap or on paper if REDCap is not feasible. REDCap will securely store the oncology physician's email address for surveys. In addition to the email address, the only personal identifying information the oncology physicians will provide will be their name, age, ethnicity, and the name of the clinic where they work. The oncology physician will be assigned an ID number, which will be used to link their surveys together with those of patients enrolled in the study.

The oncology physician also will agree to have one visit per each patient audio-recorded (baseline). All parties present for recorded office visits, including: enrolled patients, any accompanying caregivers, family or friends, the oncology physician, and any other physicians or health care providers not participating in the study will be fully aware that the conversation is being audio-recorded and will provide verbal assent immediately before any recording begins, in addition to the prior written consent of enrolled subjects. These audio-recordings will be labeled and stored using the patient's initials and date.

The participation of oncology physicians in the research study meets criteria for "waiver of documentation of consent" because the research involves no more than minimal risk to the oncology physicians and there are no procedures for the oncology physicians that would normally require written consent outside of the research context.

6. REGISTRATION

6.1. Prior to entering participants (i.e., oncology physicians, patients, caregivers) on this protocol, the following must be on file at the URCC NCORP Research Base:

6.1.1. Documentation of IRB approval in the form of an HHS Protection of Human Subjects Assurance Identification/IRB Certification/Declaration of Exemption (formerly Form 310), CTSU approval form or signed letter from the IRB.
6.1.2. A copy of the institution's IRB-approved informed consent document.
6.1.3. Written justification for any substantive modifications made to the informed consent concerning information on risks or alternative procedures.

These documents are submitted to:
Cathleen_Lesniewski@urmc.rochester.edu
OR
Ms. Cathy Lesniewski
URCC NCORP Research Base
Saunders Research Building
265 Crittenden Blvd
CU 420658
Rochester, NY 14642

6.2. Registration Requirements

6.2.1. Timing of Registration: See section 11, Outline of Study-Specific Procedures.

6.2.2. Screening Registration

6.2.2a. Go to the URCC NCORP Research Base website at URCC-NCORP.org and enter the information outlined below.

- Additionally if you experience difficulties you may call 585-275-5513 at the University of Rochester Cancer Center to verbally give the URCC registrar the information below.

- The following information will be requested:
  - NCORP Affiliate Name
  - URCC Protocol
  - Most recent IRB approval date (either initial or annual)
  - NCORP Practice Site
  - Name and telephone number of person registering study participant
  - Confirmation that consent form has been signed and by whom
  - Confirmation of patient screening ID if participating in another URCC study
  - Patient's Physician Name (confirms that oncology physician has been enrolled)
  - Patient's identification
    - First and last NAMES

6.2.3. Baseline Registration

6.2.3a. Go to the URCC NCORP Research Base website at URCC-NCORP.org and enter the information outlined below.

- if you experience difficulties you may call 585-275-5513 at the University of Rochester Cancer Center to verbally give the URCC registrar the information below.
- The following information will be requested:
  - NCORP Name
  - URCC Protocol
  - Most recent IRB approval date (either initial or annual)
  - NCORP Practice Site
  - Name and telephone number of person registering study participant
  - Confirmation that all eligibility requirements have been met
  - Confirmation that consent form has been signed and by whom
  - Patient's screening ID
  - Patient's Physician Name (confirms that oncology physician has been enrolled)
  - Patient's identification
    - First and last NAMES
    - Birth date (MM/DD/YYYY)
    - Gender
    - Race/Ethnicity
    - Five-digit zip code
    - Payment code
    - Confirmation of patient assessment scores
    - Date of Baseline Visit
    - Patient's preferred and alternate phone numbers and best time to call (for the Telephone Team Call 1 to 7 days after the baseline visit) and mailing address to contact the patient if telephone call is not feasible.
    - Whether the patient has a caregiver enrolling in the study

  - Caregivers providing consent will require the following for registration:
    - First and last NAMES
    - Birth date (MM/DD/YYYY)
    - Gender
    - Race/Ethnicity
    - Five-digit zip code
    - Payment code
    - Caregiver's preferred and alternate phone numbers and best time to call (for the Telephone Team Call 1 to 7 days after the baseline visit
    - Relationship to patient
    - Confirmation of caregiver consent

- **An email confirmation of registration will be forwarded by the URCC Research Base.**

6.3. A total of 528 patients and maximum of 528 caregivers is planned. Each patient will be able to choose one caregiver to participate.

6.4. This protocol is open only to affiliates of the URCC NCORP Research Base who provide written documentation of IRB approval. There will be no accrual at the URCC NCORP Research Base itself.

7. OUTCOMES

The outcomes of this study were informed by preliminary data that show that older patients and caregivers rate communication about age-related issues as very important.

7.1. NCI-specified Primary Outcome

The Health Care Climate Questionnaire (HCCQ) [82-85] measures patient-centered autonomy-supportive physician behaviors and satisfaction with communication such as whether the patient and caregiver feel that the physician understands his/her perspective, provides choices and options, and encourages participation in decisions. The measure has been studied and validated in older patients. Similar to other studies which adapt satisfaction scales to capture specific clinical criteria (e.g., satisfaction with the physician regarding communication about chemotherapy),[86] the HCCQ has been modified for this study (HCCQ-age) to specifically address patient satisfaction with oncology physician behaviors and communication regarding age-related issues and concerns in order to specifically address satisfaction with the intervention (geriatric assessment summary and recommendations) rather than satisfaction with other aspects of cancer care (e.g., communication about cancer treatment). As is done with satisfaction with care surveys in other research and in clinical settings, the HCCQ (both modified and original) will be administered within 1 to 7 days after the baseline audio-recorded clinic visit.[87-90] These measures will be obtained via a phone call administered by trained personnel (or mailed if a telephone call within 2 weeks of the baseline visit is not feasible). If this is not completed, the 4-6 week visit HCCQ data will be used in its place. Our University of Chicago collaborators (Dale and Gorawara-Bhatt) have experience with the conduct of such assessments over the phone and this minimizes perceived or real influence from the oncology physician or Research Base. The University of Chicago collaborators and research staff, who are subcontracted through PCORI, will be blinded to group assignment as the Telephone Team. Press-Ganey measures of satisfaction of overall care will be collected at week 4 and in follow up visits. The HCCQ will also be completed as part of the patient and caregiver packets in follow up time points for comparison.

Dr. Epstein's research team has extensive experience[76,85] with the use of the HCCQ and has captured this measure in 81 patients similar to those who will be recruited for the proposed study (older patients with advanced cancer). Patients were recruited to a NCI-funded study that is evaluating a coaching intervention to improve physician communication behaviors.[91] Because the study utilizes a cluster randomized design, an intracluster correlation coefficient (ICC) was estimated from existing data to assist with sample size calculations for the current proposal. HCCQ has 5 questions measured on a Likert scale of 1-5, which are added to create a score between 5-25. Ceiling effects are common with HCCQ and with patient satisfaction scales in general, although the modified version likely has less of a ceiling effect due to its focus on a specific clinical scenario. Despite the ceiling effects, policy makers have utilized patient satisfaction as a key measure for reimbursement in clinical practice, with a focus on obtaining "perfect" scores.

For example, the Hospital Value-Based Purchasing (VBP) Program[92] is a Centers for Medicare & Medicaid Services (CMS) initiative established by the Affordable Care Act of 2010 to reward acute-care hospitals with incentive payments for the quality of care they provide to people with Medicare. Incentive payments are funded by a withhold of 1-2% of each hospital's base operating DRG payments for each patient discharge over a year, placing hospitals "at risk" for this amount based upon performance on quality measures. It is estimated that roughly half of participating hospitals will receive a net decrease in payments, while the remainder will receive a net increase in payments resulting from superior performance. The HCAHPS (Hospital Consumer Assessment of Healthcare Providers and Systems) survey, which is the first national, standardized, publicly reported survey of patients' perspectives of hospital care, represents the performance measurement for the VBP Patient Experience of

Care Domain. The HCAHPS survey is quite similar to the HCCQ in the questions that capture satisfaction with perceived understanding of patient's values and goals by the physician and communication about health care choices. In terms of reimbursement, CMS uses performance on nine measures from HCAHPS and each hospital's "top-box" raw score—the percentage of a hospital's patients who chose the most positive, or "top-box"—is utilized to calculate their Achievement Points and Improvement Points. In this manner, the HCAHPS score is used as a binary variable, with hospital performance and, therefore, payment depending upon the survey respondent's choice of either the highest rating or any other rating.

As a result, an intervention that can lead to the slightest improvement in patient satisfaction scores has important policy implications. This is evident by studies that demonstrate the effectiveness of models of care interventions with small differences in patient satisfaction scores.[87,93-95]

7.2. Secondary Outcomes

Secondary aim 1 will evaluate whether the GA intervention increases discussions regarding age-related issues between the patients, caregivers and oncologists. This measure of direct communication was accepted by PCORI as the primary aim for the study. For this aim, the first medical consultation after GA administration will be audio-recorded. This data will be sent to the URCC Research Base and there it will be transcribed and content analyzed with the aid of a checklist to determine whether the topics included in the GA were discussed and to record the total length of consultation. Audio-recordings will be transcribed for quantitative analysis of the communication processes, including number of questions asked and topics discussed.[57,76,96-98] Methodology has been established in prior work by Dr. Epstein's group. For each transcript, coding will be performed directly by 2 trained raters who *will be blinded* to group assignment. A summary of coding procedures including assessment of inter-rater reliability and resolution procedures for disagreement is included in Appendix X-8.

The coding schema includes definitions for each code and the specific steps the coders performed during the coding process. The coding procedures involve an initial reading of the transcript to identify specific geriatric concerns and the initiator of the concerns, followed by a second reading, in which response quality and interventions implemented due to concerns are identified.

Five coders were involved in the coding process, with 20 percent of the transcripts coded by all five coders to establish and maintain inter-rater reliability; the remaining transcripts were all dually coded. For each transcript, whether dually coded or coded by the entire coding team, a consensus was agreed upon and a final coding table created for that transcript. These final consensus tables are what will be utilized in the analysis of the data.

Due to the coding schema involving a conditional coding structure, inter rater reliability involved percent agreement in 3 coding areas: number of geriatric concerns, the category of geriatric domain discussed, and the physician's response quality. *A GA communication composite score will be created by adding all GA-related topics that were discussed for each visit.* For number of geriatric concerns, the percent agreement was calculated using the difference in numbers between each coder and the consensus. An average of all coder agreement was calculated for the category of geriatric domain and physician response quality. If inter-rater agreement percentages fell below 70%, the coding team would meet for additional training. The principal investigator remained involved in the coding process and provided guidance or adjudication when necessary.

Another secondary outcome measure will be patient-reported HRQoL as measured by the FACT and caregiver HRQoL (burden) as measured by the Caregiver Reactions Assessment (CRA). We hypothesize that the mean HRQoL for patients and caregivers at sites randomized to the intervention arm will be higher than for those in the usual care arm at 4-6 weeks following the intervention and that this increase will be both statistically significant and clinically meaningful.[99,100] We will include GA impairment (at baseline and follow up) to evaluate if these influence patient-reported and caregiver-reported HRQoL differently in the intervention versus the control group. Other secondary outcomes will include whether the intervention impacts how the oncology physician acknowledges and addresses age-related concerns, caregiver burden (as measured by the Caregiver Reaction Survey) and caregiver satisfaction with communication (as measured by the modified HCCQ) at 4-6 weeks. We will follow participants for survival for 12 months after enrollment. We will obtain the date, location of death, and cause of death. If a site becomes aware that a study participant is deceased, they should complete the form which is available on the URCC NCORP website. Otherwise sites will be contacted approximately 1 year after each participant was enrolled to assess survival and asked to complete this form.

7.3. Data Sources

7.3.1. Audio-recordings. As part of baseline procedures, a clinic visit for both arms will be audio-recorded for the analysis of content. For our secondary and exploratory aims, outcome measures are derived from audio-recordings of oncology physician-patient visits (often with a caregiver present). All enrolled patients (Arm 1 and Arm 2 groups) will have one office visit with their participating oncology physician audio-recorded (baseline visit). All parties present for recorded office visits, including: enrolled patients, any accompanying caregivers, family or friends, the oncology physician, and any other physicians or health care providers not participating in the study will be fully aware that the conversation is being audio-recorded and will provide verbal assent immediately before any recording begins, in addition to the prior written consent of enrolled subjects (oncology physicians, patients, and caregivers). Patients, caregivers and oncology physicians may receive copies of these recordings at their request.

7.3.2. Patient Surveys. Patients will complete surveys prior to the start of treatment at screening and baseline, and the Telephone Team Call within 1 to 7 days from baseline, and at 4-6 weeks, 3 months, and 6 months. We are sensitive to respondent burden and have minimized the number of items to be completed in a single sitting. All surveys have been utilized in our pilot work with older patients with cancer and other age-related health conditions. In a recent study, 98% of patients with non-small cell lung cancer with a median survival of 9 months completed a baseline battery similar in length, and 70% and 64% of those who were still alive were able to complete assessments at 3 and 6 months, respectively.[101,102] As is often true for patients with advanced disease, missing data were *not* random; sicker patients tended not to complete surveys. We have included approaches to missing data in the statistical section of the protocol.

7.3.3. Oncology Physician Surveys. Oncology physicians will complete a baseline survey prior to or when their first patient consents to the study and a brief follow-up survey at the end of the study. After the audio-recorded baseline clinic visit, oncology physicians will be asked about potentially important covariates or moderators, including disease and treatment characteristics.

7.3.4. Caregiver Surveys. Caregivers will complete surveys at the same time points as patients. However, caregivers will not receive the Telephone Team Call. We will also ask caregivers to assess satisfaction with communication and care, satisfaction with decisions, and caregiver burden (both personal and economic).[103]

7.3.5. Chart Abstraction and Claims. If there is missing information or conflicting medical information from the surveys, we will obtain medical records in order to verify information about disease location, pathology, stage, metastases, and survival status from charts. We will request information from the CRA on recommendations made and implemented. In order to assess health care utilization (e.g., adverse events such as hospitalizations) for future work on examining cost-effectiveness of the intervention, permission to obtain Medicare claims in the future will be asked on the consent form. Claims will not be obtained for any individual patient until the patient has completed study procedures. All consent and research procedures for

obtaining Medicare claims will be followed: http://www.cms.gov/Research-Statistics-Data-and-Systems/Computer-Data-and-Systems/Privacy/Researchers.html

Permission to obtain claims is voluntary. Patients will be able to decline this procedure at the time of consent. Declining consent for obtaining claims from Medicare for future research to examine cost-effectiveness, quality of care, and health care utilization does not preclude patients from participating in this study.

8. MEASURES TO BE COLLECTED

An overview of measures is provided here. A detailed description of each measure including a summary table with collection time points is provided in **Appendix X-1: Measures.**

**Patient and Caregiver Measures:**
Demographics
Medical Characteristics and Treatment
Com-meds/Polypharmacy/Baseline Labs
Geriatric Assessment
- Assessments by CRA
- GA Patient Packet
Communication & Treatment Preferences
Satisfaction and QoL
Caregiver Health and Economic Burden

We have piloted all measures. In total, geriatric assessment measures that are filled out by the patient require approximately 20 minutes of time. The additional measures require an additional 30 minutes of time. We have incorporated flexibility with timing in order to reduce patient burden. The follow-up surveys require about 30 minutes of time in total. The caregiver surveys at each time point require approximately 30 minutes of time.

Patients may complete the first geriatric assessment in clinic at time of consent or at in clinic before next visit with the oncology physician. They may choose to complete measures at home in between visits. The geriatric oncology clinic at the University of Rochester routinely captures these measures as part of clinical care.

The assessments performed by the CRA and associated surveys take about 30 minutes of time in total (including physical performance and cognitive tests). Any person at the practice site can be trained by Research Base staff to do the GA. The GA does not need to be performed by the oncology physician.

The oncology physician assessments will be done either on paper or through REDCap, whichever the oncology physician prefers. The baseline and end of study assessments take no longer than 10 minutes and after the initial patient visit, the decision-making form (to assess factors that influenced decisions) takes only a few minutes to complete.

8.1. Components of Geriatric Assessment (Patient)

Assessment tools comprising the comprehensive GA are listed in **Table 2.** The various assessment tools were selected based upon extensive data in the geriatric literature demonstrating predictive value as well as feasibility data in multiple studies of elderly patients with cancer. Other than the cognitive and physical performance measures, the assessments are self-administered. Patients who cannot complete the assessment on their own will receive assistance from study personnel or from a caregiver. The GA is performed before baseline registration. Follow-up GA measures are collected at 4-6 weeks, 3 months, and 6 months.

Table 2: Components of the Comprehensive Geriatric Assessment

DOMAIN	TOOL	SCORE SIGNIFYING IMPAIRMENT
**Physical function**	➢ Activities of Daily Living<br>➢ Instrumental ADLs<br>➢ Fall History<br>➢ OARS Physical Health	➢ Any ADL deficit<br>➢ Any IADL deficit<br>➢ Any history of falls<br>➢ A lot of difficulty with any task
**Objective physical performance**	➢ Short Physical Performance Battery<br>➢ Timed "Up and Go"***	➢ ≤ 9 points<br>➢ >13.5 seconds
**Comorbidity**	➢ OARS Comorbidity***	➢ Patient answered "yes" to ≥3 chronic illnesses<br>➢ One illness interferes "a great deal" with QoL
**Nutrition**	➢ Body Mass Index<br>➢ Mini Nutritional Status***<br>➢ Weight loss***	➢ <21 kg/m^2<br>➢ ≤ 11 points<br>➢ >10% from baseline weight
**Social support**	➢ OARS Medical Social Support***	➢ Patient answers one of the social support questions indicating less than adequate support for care
**Polypharmacy**	➢ Polypharmacy<br>➢ Lab***	➢ ≥5 regularly scheduled prescription medications OR<br>➢ Any high-risk medication OR<br>➢ Creatinine clearance <60 ml/min
**Psychological**	➢ GAD-7 ***<br>➢ Geriatric Depression Scale	➢ ≥ 10 points<br>➢ ≥5 points
**Cognition**	➢ Blessed OMC***<br>➢ Mini-Cog	➢ >10<br>➢ 0 words recalled OR 1-2 words recalled + abnormal clock drawing test

*****Captured before baseline registration**

Abbreviations: ADL (Activities of Daily Living); Blessed OMC (Blessed-Orientation Memory Concentration Test); GAD (Generalized Anxiety Disorder 7-Item Scale); GDS (Geriatric Depression Scale); IADL (Instrumental Activities of Daily Living); QoL (Quality of Life).

8.2. Other Clinical Measures (Patient and Caregiver)

8.2.1. Sociodemographics* ***(patient and caregiver):*** Age, race and ethnicity, gender, highest level of education achieved, employment status, marital status, and presence of a living companion will be captured. We will also assess financial concerns and understanding of disease.

8.2.2. Tumor and Treatment Characteristics *(patient):* The tumor stage, previous surgery or radiation, previous cancer treatment, current cancer treatment plan (if any) will be captured by the CRA.

8.2.3. QOL, Symptoms, and Distress *(patient and caregiver):* Quality of life measures will include assessments of distress and symptoms. Quality of life and symptoms will be measured using validated assessments. Caregivers will complete validated measures to assess impact of caregiving on their quality of life. Caregiver economic burden will be assessed including time required to give care.

8.3. Processes of Communication and Experience of Care Assessments (Patient/Caregivers and Oncology Physician)

8.3.1. Patient/Caregiver Assessments: We will collect measures to assess patient and caregiver satisfaction with communication, experience of care, and decision-making preferences. The measures chosen for this

study are validated tools designed to measure patients' and caregivers' confidence in their ability to communicate their concerns, obtain and understand information, ask questions, clarify uncertainties, and make sure that their oncology physician understands them. We will measure understanding of prognosis, acceptance of terminal illness, and perceived uncertainty in choosing options. At follow up visits, patient and caregiver satisfaction with the decision and experience of care will be assessed with validated tools.

8.3.2. Oncology Physician Assessments: We will collect information on sociodemographics and decision-making preferences. Oncology physicians will be presented with a clinical scenario of elderly cancer patients with a variety of geriatric-related impairments (i.e., physical frailty, cognitive impairment). A series of questions will follow each vignette inquiring about the likelihood of the oncology physician to offer chemotherapy and cancer treatments in the scenario. After the study is over, physicians will be asked to complete a brief follow-up survey. After the audio-recorded clinical encounter, oncology physicians will complete a short follow-up survey (<10 questions) requesting information on the plan for the patient and factors that influenced how the decision was made.

8.3.3. Audio-recordings of oncology physician-patient visit: A CRA will audio-record the patient-oncology physician consultation (baseline study visit). For our Secondary and Exploratory aims, we will assess the number of age-related concerns brought up by patients and caregivers. ***Coders*** *will undergo extensive training and supervision by developers of the scales, will not be involved in any other aspects of the study, and will be blind to study hypotheses and assignments for intervention vs. control.* Please refer to Appendix X-8 for full coding procedures.

9. NCORP PRACTICE SITE RANDOMIZATION

A practice site is defined as any practice location within an overarching NCORP designation where oncology physicians and study staff work independently, i.e., do not cross over into another site. Practice sites will be randomized to one of the two study arms by means of a computer–generated randomization table. The randomization process will be determined using R software provided by Dr. Charles Heckler, the lead biostatistician of the URCC NCORP Research Base.

Sites are randomized on a continual basis due to a variety of factors (i.e., a new NCORP Community Affiliate has been added to the URCC NCORP Research Base network, a new oncology practice site has been added to an existing NCORP Community Affiliate, or an oncology practice site that is currently affiliated with an NCORP Community Affiliate has expressed interest in participating in the COACH study). Past accrual to URCC studies (or NCORP Cancer Control studies if URCC accrual information is not available for new Affiliates and sites) is used to stratify each practice site as a **large** accruing (20 or more accruals/year) site or a **small** accruing (less than 20 accruals/year) practice site in order to assure balance in the randomization. The general assumption will be that any new site will be considered "small", unless it is determined based on past accruals that they are large. For new oncology practice sites that meet the definition for being independent (i.e., physicians and staff that do not cross over into another site), the next unassigned randomization allocation, from the randomization table will be used to assign their study arm. The randomization table takes into account size of the practice site. If a new oncology practice site is added at a later time during the study with physicians/staff that also see patients (cross over) at both or multiple practice sites, the new practice site will be assigned to the same study arm as the already randomized practice site where the physician/staff also sees patients.

Once sites have IRB approval on file with the Research Base and at least one member of research staff is trained on all study procedures, they are notified of their randomization allocation by an email from the study PI, Dr. Mohile. Practice sites names and randomization assignments are saved in the database, this is used to link to which arm each study patient belongs to during patient registration.

The original sample size calculation (Section 15) was based on the randomization of the 16 NCORP oncology practice sites. During study startup, as the structure of the new NCORP affiliates unfolded, more practice sites than was anticipated were interested in participating. The original protocol included the ability for more sites to participate, since the increase in the number of the clusters also increases the statistical power of the study.

10. INTERVENTION OVERVIEW FOR PHYSICIANS

Practice sites will be notified if they are in the intervention arm by URCC Research Base staff. Prior to the intervention, oncology physicians will receive training on how to best utilize GA information in clinical practice for older adults with cancer. The training session provides a brief overview of the intervention information and can be completed through a web conference, telephone call, or as a paper "sign-off" documenting review of the materials. The training materials were developed with resources from Drs. Mohile's and Hurria's geriatric oncology lectures.

The CRAs and/or research nurses at sites randomized to the intervention arm will be trained to utilize the *mycarg.org website* to derive a summary of GA scores and a list of targeted GA-driven recommendations.

This information will be printed by the site CRAs for the oncology physician who will "sign off" that he/she has received and reviewed the information. Study or clinical staff can assist the physician in checking which recommendations were considered and facilitating implementation of GA-driven recommendations. Two copies of the summary and checklists should be made: one should be provided to the patient/caregiver and one should be retained in the study chart. Patients and caregivers should be provided with a copy of the GA summary and GA-driven recommendation forms prior to the audio-recorded visit. The original forms should be submitted to the URCC Research Base.

11. OUTLINE OF STUDY-SPECIFIC PROCEDURES

**Usual Care Site (Arm 2)**	**Intervention Site (Arm 1)**

Oncology Physician Enrollment – Baseline survey on REDCAP

Subject Identification Process

Screening: Visit 0

- Consent patient & caregiver; complete screening registration.
- If time permits, administer GA measures (cognitive, physical performance, and nutrition) to patient.
- Subjects (patient and caregiver) complete screening packet during visit or take the screening packet & baseline packet home with them to complete prior to the baseline visit.
- CRA completes CRA screening study forms.
- Confirm subjects will bring completed packets to next visit or schedule additional time if needed to complete at visit.

Score GA & Complete Baseline Registration

- When patient and CRA screening forms complete, score each GA measure as per training procedures (at screening or beginning of baseline visit prior to study visit with oncology physician).
- Complete baseline registration for patient & caregiver if patient has 1 or more domains that meet cut-off score for impairment (other than polypharmacy).

Baseline: Visit 1 – Usual Care

- Inform oncology physician if depression (GDS) or cognition (BOMC) assessments score ≥ 11.

Baseline: Visit 1 – Intervention

- CRA enters GA Score on MYCARG.ORG
- Print and provide GA summary and recommendations forms to oncology physician, patient and caregiver.
- Information on cognitive impairment or depression is included in summary.

- Study visit with oncology physician occurs - **audio record** this visit
- Oncology physician completes forms about patient
- Submit all materials to URCC within 7 days.

Telephone Team Call to Patient within 1 to 7 days of Baseline Visit

Health Care Climate Questionnaires will be obtained via a phone call administered by trained personnel blinded to group assignment within 1 to 7 days of the baseline audio-recorded clinic consultation.

All Follow-up Visits: Visit 2*, Visit 3, Visit 4

(Visits are 4 to 6 Weeks, 3 months, and 6 months from Baseline)

Before Visit: Confirm patient & caregiver will bring completed packets to study visit.

During Visit: Administer cognitive and physical performance measures and complete CRA study forms.
Inform oncology physician if depression (GDS) assessment score ≥ 11.
**Intervention Arm ONLY: CRA complete GA-driven recommendation follow-up forms at visit 2*

After Visit: Submit all forms to URCC Research Base within 7 days.

11.1. Procedures Prior to Screening Visit

11.1.1. Oncology Physician Enrollment and Participation

11.1.1a. If an oncology physician is interested in the study and meets the inclusion/exclusion criteria, he/she will agree to participate on paper or through REDCap (sent by the URCC Research Base).

11.1.1b. The oncology physician will complete a baseline survey on demographics and treatment preferences either on paper or through REDCap.

11.1.1c Ideally oncology physicians are enrolled as soon as possible. However, an oncology physician can be enrolled after his/her patient is identified for the study or prior to screening registration for the first identified patient.

11.1.2. Subject Identification Process

11.1.2a. Patients: Once a site is IRB approved, the site study staff can start screening for patients according to the inclusion/exclusion criteria for patients (Section 4.2). Only patients from enrolled physicians can be registered.

11.1.2b. Screening Log: A screening log will be kept at each participating site, where *both* patients and caregivers approached for the study will be entered by site study staff.

11.2. Screening: VISIT 0

11.2.1. Patient & Caregiver Recruitment & Consent Process

11.2.1a. Patients

- The site study staff will notify the patient's oncology physician when a patient is identified as a possible candidate for the study. Site study staff should screen for patients who may fit eligibility criteria for all requirements other than GA impairment. It is anticipated that some of the patients who are eligible will be new patients for the oncology physician.
- The oncology physician and/or CRA should mention the study to the patient and give out recruitment materials.
- If the patient is interested, the oncology physician and/or site study staff will explain the study and once all aspects of the study have been discussed to the patient's satisfaction, the voluntary written informed consent procedures will be completed with the patient if they choose to enroll in the study.
- Patients should choose a caregiver to participate. As much as possible, caregivers should be recruited to be part of the study when the patient is recruited. In the rare circumstance that there is no willing caregiver to participate, the patient will be allowed to participate without a caregiver. URCC Research Base staff should be notified prior to patient registration if there is no designated caregiver.
- The site study staff can schedule a separate visit with the patient and/or caregiver to go over the consent and initiate study procedures if more time is needed.

11.2.1b. Caregivers

- If a caregiver, chosen by the patient, is interested in the study and meets the inclusion/exclusion criteria, the site study staff will confirm eligibility (as per section 4.3) and complete the informed consent procedures with the caregiver. Caregivers must enroll in the study before or on the baseline visit.

11.2.2. Patient & Caregiver Assessment Process

11.2.2a. Once a patient has consented, screening and baseline assessments/procedures can occur. Site

study staff can schedule a separate visit with the patient and caregiver to complete study procedures if more time is needed.

11.2.2b. The procedures for screening will consist of the steps below:

- Confirm that the patient's oncology physician has been enrolled onto the study and if not, follow procedures for enrolling oncology physicians.
- Patients meeting eligibility criteria are initially registered with a screening registration after signing consent.
- Administer Cognitive, Physical, and Nutritional Assessments to the patient.
  - These assessments can be administered right after consent, at a separate visit scheduled by site study staff, or just prior to the next oncology physician visit. Allow 45 minutes to perform assessments if done just prior to an oncology physician visit.
  - Anyone at the practice site can administer cognitive and physical assessments to the patient as long as they have participated in the required GA training administered by the URCC Research Base. CRAs, clinical nurses, and technicians are all eligible to participate in training and can complete GA study procedures.
- Patient will complete all screening surveys.
  - Ideally the patient will complete either at the time of consent or the screening and baseline packets can be taken home and completed before the next visit with the oncology physician. If the patient needs assistance from site study staff to complete surveys, allow adequate time. Approximately 60 minutes is recommended.
- Abstract required medical information from chart.

11.2.2c. The GA must be completed prior to the study visit with the oncology physician. The study visit with the oncology physician will be audio-recorded with the oncology physician and patient. The caregiver should be encouraged to attend this study visit, but the study visit can occur without the caregiver present if the caregiver is not available.

11.2.2d. The procedures for the caregiver assessments will consist of the steps below:
Caregivers will complete all baseline surveys**.** Caregivers will complete either at the time of consent or the packet can be taken home and should be completed before the next visit with the oncology physician.

11.3. Baseline: VISIT 1

11.3.1. Patient & Caregiver Assessment Process

11.3.1a. BEFORE BASELINE VISIT:
Site study staff will telephone the participant to:

- Confirm scheduled visit with the oncology physician.
- Confirm that the patient and caregiver completed the screening surveys before the oncology physician visit and remind them to bring the surveys in with them.
  - Schedule the patient to come in to meet with site study staff at least 45 minutes prior to the oncology physician visit in order to complete study procedures. Allow for more time if patient and caregiver communicate(s) that surveys have not been completed. Only patients who complete GA procedures can be registered for the study.

11.3.1b. AT THE BASELINE VISIT:

- If the patient's screening surveys have not been completed prior to the patient coming in, complete them now.
- If the caregiver's surveys have not been completed prior to the caregiver coming in, complete them now**.**
- If not complete staff should administer cognitive, physical performance, and nutrition assessment.
- If not complete, staff should complete CRA study forms.

- If not complete, complete baseline surveys. The baseline surveys do not need to be completed prior to registration, but should be completed on the day of the baseline visit. For example, patients can complete baseline surveys while receiving cancer treatment that same day.
- Score each GA measure as taught in the training procedures. If assistance is needed for scoring, contact the URCC NCORP Research Base.
- Patients who have at least one abnormal GA score other than polypharmacy can move forward with the study.
- After the GA and the above steps are complete, the baseline registration procedures can be completed for the patient and caregiver (refer to section 6, Registration).
  - Registration must occur prior to baseline study visit with the oncology physician.
- Prior to the study visit with the oncology physician, if depression (GDS) or cognition (BOMC) assessments score ≥ 11, inform patient's oncology physician as follows:
  - Usual Care arm -- inform oncology physician with template as per training.
  - Intervention arm -- information on cognitive impairment or depression is included in summary (see section 11.3, *Intervention Procedures*).

11.3.2. Intervention Procedures (Only <u>for sites randomized to the intervention arm</u>)

11.3.2. a. AT THE BASELINE VISIT:

- After the patients have been registered for the study, enter the GA scores into the mycarg.org website or if no internet is available, contact the URCC NCORP Research Base to assist.
- After entering the GA scores, print the built pdf packet specific to that patient.
- Present GA summary form and recommendation forms to oncology physician to review just prior to baseline patient-oncology physician visit. Study staff can assist the oncology physician in the completion of the forms. Study and clinical staff can assist in implementing GA-driven recommendations after physician approves them.
  - Information on cognitive impairment or depression is included in the summary.
  - The oncology physician MUST review and sign the summary form and recommendations forms.
  - GA recommendations that are planned by the oncology physician for the patient should be check-marked by the end of the visit.
- Three copies of the GA summary and GA recommendations forms should be made: one should be provided to the patient, one should be provided to the caregiver (if requested), and one should be retained in the study chart.
- The original forms should be sent into the URCC Research Base.

11.3.3. Oncology Physician Assessment for Patients: After the audio-recorded baseline visit, oncology physicians will complete a brief survey (on paper) to capture factors that influenced the decision-making process for the patient's plan.

11.3.4. Submitting Materials to the URCC Research Base:

11.3.4a. After the audio-recorded baseline visit, site study staff should ensure the forms below are completed and submit them to the URCC Research Base:

- All oncology physician, patient and caregiver assessments from above
- All CRA Study Forms
- Audio-recordings (submitted and stored as per training) with Audio-recording Data Form
- Intervention summary and recommendation forms (if applicable).

11.3.5. Audio-recordings (for both intervention and usual care)

11.3.5a. The first visit with the oncology physician following the completion of GA measures will be audio-recorded for analysis of content and process of communication.

11.3.5b. Site study staff need to ensure that two audio-recorders are on and working during the visit, but do not need to be present in the room during the visit.
11.3.5c. Site study staff will submit audio-recordings as per training and complete the audio-recording data form.

11.4. Follow-up Visits

11.4.1. Visits will occur 4-6 weeks after the baseline audio-recorded visit, 3 months after the baseline audio-recorded visit (10-14 weeks), and 6 months after the baseline audio-recorded visit (20-24 weeks).

11.4.1a. BEFORE EACH FOLLOW-UP VISIT:
Study staff will telephone the participant to:

- Confirm scheduled visit with the oncology team. The visit does not need to be scheduled with the oncology physician.
- Confirm that the patient and caregiver completed the surveys.
  - Surveys must be completed during the appropriate window for each time point. Site study staff can either mail the follow-up surveys to the subjects OR at the end of one visit, they can provide the next round of surveys to the subjects to take home with them for the next follow-up visit.
  - Ideally subjects will complete the survey packet at home and bring to site study staff on the day of a routinely scheduled visit for the oncology physician visit or designee (e.g., nurse practitioner) or cancer treatment.
  - If the patient needs assistance from site study staff to complete surveys on-site, allow adequate time (45-60 minutes).
  - If patients and caregivers cannot complete packet at home:
    - Patient and caregiver can come in prior to office visit to complete surveys.
    - Patient and caregiver can complete surveys during cancer treatment visits (e.g., chemotherapy infusion).
    - If needed, the patient and caregiver can meet with the site study staff at an additional time to complete the follow-up surveys. The subjects do not need to meet with the oncology physician to complete the surveys.

11.4.1b. AT EACH SCHEDULED FOLLOW-UP VISIT:

- *Staff* will administer the following assessments to the patient:
  - Cognitive and Physical Performance.
    - If depression (GDS) assessment score $\geq$ 11, both usual care arm and intervention arm inform patient's oncology physician with template as per training.
- *CRA*: Complete all CRA follow-up study forms. CRAs at practice sites randomized to the **intervention arm** will complete the GA-Recommendation Follow-up forms by comparing them to the GA-Recommendation Baseline forms, clinic note, and referring to the oncology physician (if needed).
- *Patient*: Complete all surveys in the follow-up packet.
- *Caregiver*: Complete all surveys in the follow-up packet.
- Site study staff will submit the following to the URCC Research Base:
  - All oncology physician, patient and caregiver surveys from above.
  - All CRA study forms.

11.4.1c. A subset of the chart notes will be requested if there are discrepancies or missing information in key data.

11.5. Telephone Team Call (1 to 7 days from Baseline)
The Telephone Team will consist of University of Chicago and University of Rochester research staff and will be blinded to group assignment. The Telephone Team will make a phone call to the patient subject within 1 to

7 days, or up to 2 weeks, of the baseline clinic consultation to assess patient satisfaction with communication about age-related issues and concerns. The phone call will be recorded.

11.5.1. BEFORE THE PHONE CALL:

11.5.1a. Site Study staff will provide the patient subject's preferred and alternate phone numbers, best time to call, and mailing address as part of the registration process for the patient. Caregiver phone number will be obtained as a back-up.

11.5.1b. To ensure that the Telephone Team remains blinded to the patient's group assignment, the Telephone Team will obtain the contact information (phone number and mailing address) from the URCC Research Base.

11.5.1c. The study materials will be mailed (with a return envelope provided) to the patient by the Telephone Team if a telephone call is not feasible (e.g., due to sensory or disability impairments).

11.5.2. DURING THE PHONE CALL:

11.5.2a. The Telephone Team will administer the Health Care Climate Questionnaires to the patient. Telephone Team will submit the Health Care Climate Questionnaires to the URCC Research Base and keep a copy labeled with patient identification number on site.

12. REIMBURSEMENT

In order to improve study retention and compliance, we will compensate patients and caregivers for their participation (i.e., time and travel). Patient and caregiver subjects will each receive $15.00 for every time point assessment completed. Both patients and caregivers can receive up to $60.00 each if all four time point assessments are completed.

13. ADVERSE EVENT REPORTING

13.1. Risks from participating in this research are psychological distress from completing the questionnaires and the cognitive testing.

13.2. ***ADVERSE EVENTS RELATED TO STUDY PROCEDURES AND NOT TO ROUTINE ONCOLOGY TREATMENT AND CARE*** will be reported using the URCC Adverse Event form. This form can be found on the URCC NCORP Research Base website.

13.3. Adverse events will be reported in accordance with the following guidelines:

	Grade 1	Grade 2			Grade 3				Grade 4		Grade 5	
	Unexpected and Expected	Unexpected		Expected	Unexpected		Expected		Unexpected	Expected	Unexpected	Expected
		With Hospital-ization	Without Hospital-ization		With Hospital-ization	Without Hospital-ization	With Hospital-ization	Without Hospital-ization				
Unrelated Unlikely	Not Required	Not Required	Not Required	Not Required	Not Required	Not Required	Not Required	Not Required	10 Calendar Days	Not Required	10 Calendar Days	10 Calendar Days
Possible Probable Definite	Not Required	10 Calendar Days	Not Required	Not Required	10 Calendar Days	10 Calendar Days	Not Required	Not Required	24-Hour; 5 Calendar Days	10 Calendar Days	24-Hour; 5 Calendar Days	10 Calendar Days

Hospitalization is defined as initial hospitalization or prolongation of hospitalization for ≥ 24 hours, due to adverse event related to study procedures.

13.4. Submit written adverse event reports in one of the following ways:

(1) PDF by email:	Cathleen_lesniewski@urmc.rochester.edu
(2) By mail:	**Cathleen Lesniewski**<br>URCC NCORP Research Base<br>Saunders Research Building<br>265 Crittenden Blvd<br>CU 420658<br>Rochester, NY 14642
(3) By fax:	Attn: Cathleen Lesniewski 585-461-5601

13.5. An *unexpected* adverse event is defined as any adverse experience, the specificity or severity of which is not consistent with the risk information described in section 13.1.

13.6. A serious event refers to any event in which the outcome results in any of the following: death, a life-threatening adverse experience, inpatient hospitalization or prolongation of existing hospitalization, a persistent or significant disability, incapacity, or a congenital anomaly/birth defect. Important medical events that may not result in death, be life-threatening, or require hospitalization may be considered a serious adverse drug experience when, based upon appropriate medical judgment, they may jeopardize the participant and may require medical or surgical intervention to prevent one of the outcomes listed in this definition.

13.7. ONLY serious adverse events related to the study procedures need to be reported for data and safety monitoring purposes. AEs and SAEs related to routine oncology treatment and care DO NOT need to be reported.

13.8. Adverse events should be reported to the local IRB as per their requirements.

13.9. Data Safety and Monitoring

13.9.1. All adverse events requiring reporting will be submitted to the Research Base as described in section 13.4. Adverse events that are *serious* AND *unexpected* AND *related* will be forwarded to the study chair and the URCC Data Safety and Monitoring Committee (DSMC) chair immediately upon receipt at URCC. Additional information may be requested upon their review.

13.9.2. All adverse events reported to URCC are entered into a protocol-specific spreadsheet. Adverse event rates are monitored utilizing the spreadsheet. If a serious adverse event is being reported frequently, the study chair will conduct a detailed review. The DSMC Committee Chair will be notified and will determine if further action is required.

13.9.3. The URCC Data Safety Monitoring Committee (DSMC) will review study progress and cumulative reports of adverse events at annual meetings. An overall assessment of accrual and adverse events will enable the committee members to assess whether significant benefits or risks are occurring that would warrant study closure.

13.9.4. The URCC will notify the NCORPs immediately of any serious safety concerns identified by the DSMC. DSMC reports will be available for download on the research base website.

14. CRITERIA FOR WITHDRAWAL

Subjects who discontinue or are withdrawn from study will be asked to complete all assessment forms up until the time of withdrawal.

If an oncology physician withdraws from the study, no further patients with this oncology physician will be recruited. Whenever possible, patients who have already been enrolled should complete study procedures and their data will be included in the analyses.

Participation in this study is voluntary. Participants are free not to take part or to withdraw at any time, for whatever reason, without risking loss of present or future care they would otherwise expect to receive. In the event that a patient does withdraw from the study, the information they have already provided will be kept in a confidential manner. Participants may discontinue participation in the study at any time if they decide they do not wish to take part any longer. Participants may be withdrawn from the study by research personnel if it is deemed in their best interest to no longer participate.

15. STATISTICAL PLAN

15.1. Statistical Considerations

This is a cluster-randomized trial with NCORP practice sites being the clusters. Because of the cluster randomized study design, we will apply linear mixed model methodology.[104] The outcome will be the response, and the arm will be the fixed effect. NCORP practice sites will be entered as a random effect independent of residual error. Estimation will be performed using Restricted Maximum Likelihood, and the null hypothesis of zero mean difference between arms will be tested using a F test.[105] The specific NCORP practice site differences will be assessed graphically using Best Linear Unbiased Predictors (BLUP) of the mean response for each NCORP.

All regression analyses will include terms to control for study site and oncologist type. In addition, clinically important socioeconomic variables such as patient gender, age, race/ ethnicity, and cancer variables such as cancer type and treatment status will be used to control for patient-level covariates. In case a key covariate is found to be unbalanced between study arms, it will be included in the model as a potential confounder.

15.2. Sample Size for NCI-Specified Primary Aim

We will utilize the modified HCCQ to address patient satisfaction with communication regarding age-related issues. Based on an analysis of the VOICE study, the standard deviation estimate of HCCQ was 2.1. The Intracluster Correlation (ICC) was 0.14 with a 95% confidence interval from 0.01 to 0.51. Because of the large amount of uncertainty in the ICC, we calculated power curves for ICC={0.01, 0.14, 0.51}, with ICC=0.51 being the most conservative. This design (8 sites per arm and 31 subjects per site) has 80% power at the 0.05 significance level to detect a change in HCCQ of 0.6, 1.3 and 2.3 for ICC={0.01, 0.14, 0.51}, respectively. Since the best estimate of the ICC is 0.14, the expected detectable difference is 1.3. This corresponds to an effect size of 0.62. The range of the HCCQ scores (modified HCCQ for satisfaction with age-related discussions) is 7 (worst possible) to 35 (best possible). The figure to the right shows the power for a range of detectable differences (D) for ICC=0.01, 0.14 and 0.51 (Figure 3). As mentioned in Section 7.1, small changes in satisfaction scores have been interpreted in other studies to be meaningful given a focus on achievement of high satisfaction scores and the link with reimbursement. In addition to using the total score as a continuous variable, we will compare the results for each question as a dichotomous variable (5 vs <5). If the distribution is skewed, we will consider treating the scores as an ordinal variable in analyses.

Figure 3: Sample Size for NCI-specified Primary Aim

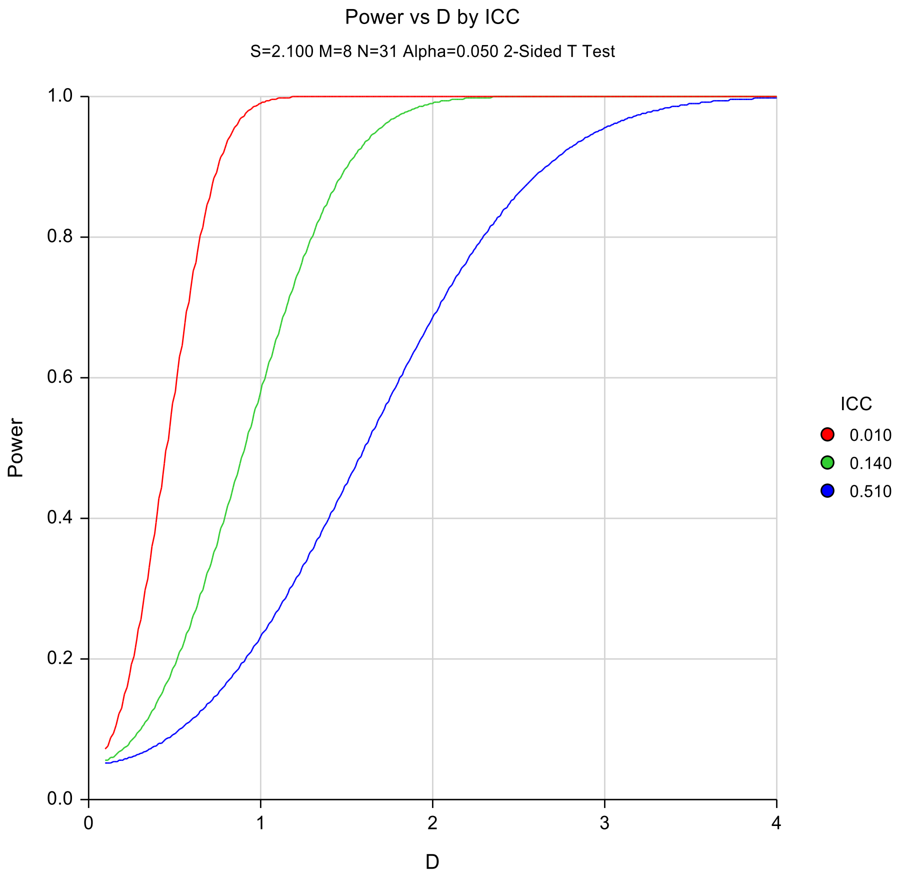

Accounting for a small dropout rate of 5% (based on our observational cohort data[1]), the targeted accrual will be **528 patient subjects total.** The dropout rate reflects patients who sign consent but withdraw prior to the audio-recorded baseline visit and capture of the modified HCCQ (which will occur within 7 days of baseline visit).

During NCORP site recruitment, if more than 16 NCORP sites are interested in participating, we will allow randomization. As of February 1, 2017, we have 68 practice sites participating in the COACH study; the increased practice site clusters provide improved statistical power. The total patient sample size will stay the same, and accrual will cease when that target is met.

Analysis for Primary Aim 1. This is a cluster randomized trial with NCORP practice sites being the clusters. Because of the cluster randomized study design, we will apply linear mixed model methodology.[104] The total modified HCCQ scores (HCCQ-age) will be the response, and the arm will be the fixed effect. NCORP practice sites will be entered as a random effect independent of residual error. Estimation will be performed using Restricted Maximum Likelihood, and the null hypothesis of zero mean difference between arms will be tested using the F test.[105] The specific practice site differences will be assessed graphically using Best Linear Unbiased Predictors (BLUP) of the mean response for each site.

15.3. Secondary Aim 1 (PCORI-specified Primary Aim)

Figure 4: Sample Size for Secondary Aim 1 (PCORI-specified Primary Aim)

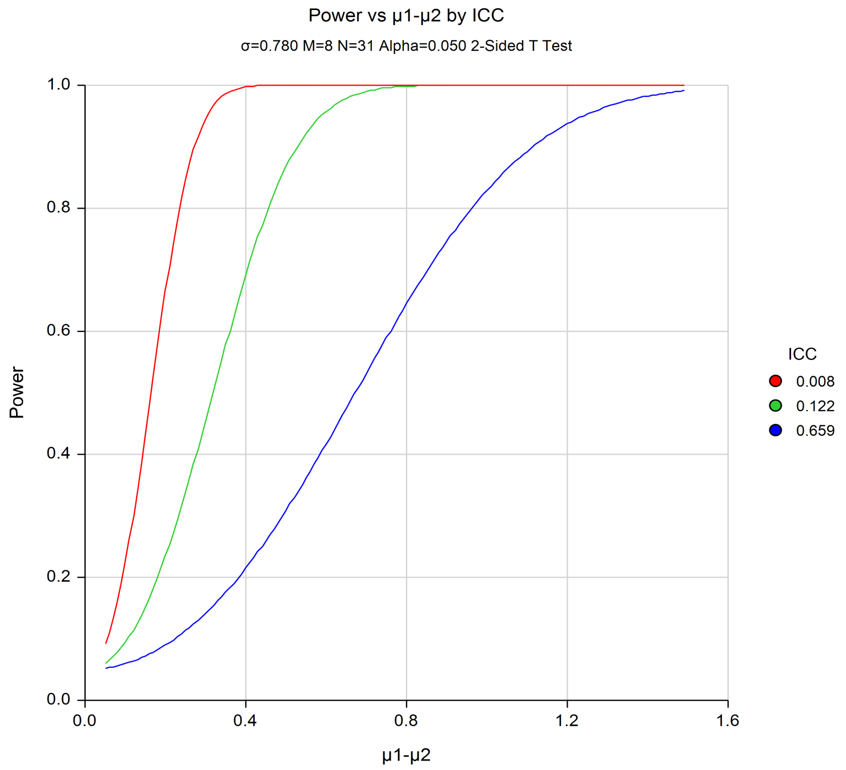

An important secondary aim and the primary aimfor PCORI is the number of discussions related to geriatric domains, as measured by the GA, brought up during the audio-recorded baseline visit. In our preliminary data from a multicenter study, the median number of discussions was 1 in 32 audio-recorded conversations between older patients, their caregivers, and oncology physicians. This preliminary work has allowed us to calculate the intracluster correlation (ICC) amongst 8 different sites for the assessment of the secondary outcome, number of discussions related to geriatric domains. The ICC was 0.122 with a 95% confidence interval from 0.008 to 0.659. Because of the large amount of uncertainty in the ICC, we calculated power curves for ICC={0.008, 0.122, 0.659}, with ICC=0.659 being the most conservative. This design (with 8 NCORP sites per arm and 31 evaluable subjects per NCORP) has 80% power at the 0.05 significance level to detect a change of 0.235, 0.456 and 0.962 in the mean number of discussions for ICC={0.008, 0.122, 0.659 } respectively, assuming a standard deviation of 0.78 (Figure 4). Since the best estimate of the ICC is 0.122, the expected detectable difference is 0.122. This corresponds to an effect size of 0.59.

The analysis for Secondary Aim 1 will be the same as for Primary Aim 1, but will use the number of discussions of age-related concerns as the response.

15.4. Other Secondary Analyses

15.4.1. Secondary Aim 2: HRQoL will be assessed with the FACT and Caregiver HRQoL will be assessed with the Caregiver Reactions Assessment (CRA). The analysis will be the same as that used for Primary Aim 1. We will include geriatric assessment impairment (at baseline and follow up) to evaluate if these influence patient-reported HRQoL differently in the intervention versus the control group. We will also compare whether the uptake of geriatric assessment recommendations influences patient reported HRQoL and caregiver burden. Data from the intervention arm will be fit to a linear mixed model with FACT or CRA as the outcome, number and percent of implemented interventions (number implemented/number recommended) as the fixed effect, and NCORP site as a random effect independent of residual error. Analyses will be adjusted for treatment status.

15.4.2. Secondary Aim 3: We will compare the effect of the intervention on caregiver satisfaction (the modified health care climate questionnaire for the caregiver) using the same linear mixed model methodology.

15.4.3. Exploratory Aims: Exploratory aims will evaluate (1) whether the number of discussions about age-related issues during the clinic visit correlates with patient satisfaction, (2) whether the intervention increases the proportion of age-related concerns that are acknowledged and addressed, and (3) if communication about age-related issues influences how patients, caregivers, and oncology physicians make decisions for cancer treatment. An additional exploratory aim will examine the impact of the intervention on survival.

In order to examine the relationship between observed communication from audio-recordings and patient satisfaction, we will evaluate the correlation between the numbers of discussions regarding age-related concerns from audio-recorded visits with patient satisfaction on HCCQ-age (modified to evaluate for satisfaction for communication about age-related issues). We will determine the association of baseline oncology physician and patient decision-making preferences on the likelihood of having a discussion related to geriatric domains. The analysis for PCORI Primary Aim (secondary aim 1) will be used with the above characteristics added as independent variables. The statistical significance and estimated coefficients will be used to identify and interpret potentially important baseline features. Any conclusions will be considered to be hypothesis generating for further research.

We will also measure the intervention increases the proportion of age-related issues that are acknowledged and addressed (e.g., oncology physician response/(number of patient and caregiver concerns). We will examine if the intervention improves physician confidence with managing age-related conditions as well as physician comfort with communication about age-related issues (using data from physician baseline and follow up forms)

We will capture survival through the participant's medical record and verification with the primary team. We will follow participants for survival for 12 months after enrollment. We will obtain the date, location of death, and cause of death. If a site becomes aware that a study participant is deceased, they should complete the withdrawal form if the survival status form is not yet available on the URCC NCORP website. Otherwise sites will be contacted approximately 1 year after each participant was enrolled to assess survival and asked to complete this form. We will determine the effect of the intervention on 12-month survival using log rank tests and survival plots.

15.5. Missing Data

Every effort will be made to encourage and facilitate participants' completion of questionnaires, but because of dropout, missing data will occur. We will evaluate the patterns of missing data and associations of missingness with other available variables. Under the missing at random (MAR) assumption, the parameter estimates from the mixed model analyses will be unbiased. If the data are suspected to be missing not at random (MNAR), a sensitivity analysis using selection and/or pattern-mixture models will be run to determine the impact on the results.[106] If the estimates are similar to the ones obtained from the simpler analysis of only complete cases, we will report the complete-case analysis results.

16. DATA MANAGEMENT AND QUALITY ASSURANCE

16.1. Training Procedures

A special training session was held at the 2013 annual URCC NCORP Research Base meeting. This training included a detailed review of the study rationale, design, and research administration procedures. Training sessions will be held with the staff from each site via teleconference and at the annual meetings. These sessions and the corresponding procedures manuals review the following: 1) informed consent; 2) completing the assessments using Teleforms; 3) completing the functional and objective measures; 4) data collection via chart extraction; 5) completing the web-based intervention using mycarg.org (for intervention arm); 6) transfer of the data to the URCC NCORP Research Base; 7) formulating the research chart; and 8) a discussion of interviewing techniques so that the research team will standardize their approaches in order to elicit consistent data from subjects. There will be a protocol update every year at the annual Research Base meeting. All assessments, data collection forms, and manuals will be readily available on the NCORP Research Base website.

16.2. Data Management

The same protocols and procedures for data quality and control that we use for all URCC NCORP Research Base protocols (which accrued over 1,000 patients in the previous year) will be used for this study. Once the patient consents to the protocol, he/she will be assigned a unique identifier by the Research Base, which will be used to link all patient data. Oncology physician and patient assessments will be captured using scannable Teleforms. The CRA and/or Research Nurse at each site will ensure that data are complete prior to submission. At the Research Base, data are scanned into an electronic password-secured Access database which is backed up every 24 hours. At the Research Base, staff to ensure that all data are collected in order to minimize missing data by employing multi-step verification procedures and querying originating sites for missing or ambiguous data. Audio-recordings will be submitted to the Research Base within 24 hours and stored securely as source documentations in accordance with HIPAA compliance. Audio-recordings will be transcribed and de-identified by persons who are not direct members of the study staff.

16.3. REDCap

Data are also collected and managed by the URCC Research Base using REDCap electronic data capture tools hosted at the University of Rochester Medical Center (URMC). We will evaluate records, clinical characteristics, and outcomes and we will utilize REDCap to collect and manage this information. Further, we will link this information to the encrypted ACCESS database (which contains the survey information) with a unique identifier.

16.3.1. The University of Rochester Medical Center provides the following information on the **REDCap program:** "Vanderbilt University, in collaboration with a consortium of institutional partners, has developed a software toolset and workflow methodology for electronic collection and management of research and clinical trial data, called REDCap (Research Electronic Data Capture). The REDCap system is a secure, web-based application that is flexible enough to be used for a variety of types of research. It provides an intuitive interface for users to enter data and real time validation rules (with automated data type and range checks) at the time of data entry. REDCap offers easy data manipulation with audit trails and functionality for reporting, monitoring and querying patient records, as well as an automated export mechanism to common statistical packages (SPSS, SAS, Stata, R/S-Plus). Through the REDCap Consortium, Vanderbilt has disseminated REDCap for use around the world. Currently, over 240 academic and non-profit consortium partners on six continents with over 26,000 research end-users use REDCap"

16.3.2. ***According to the University of Rochester Clinical and Translational Science Institute (CTSI), REDCap is supported with the following means***. "The *CTSI Informatics Core*, a unit of the SMD *Academic Information Technology (AIT) Group,* will serve as a central facilitator for data processing and management. REDCap data collection projects rely on a thorough study-specific data dictionary defined in an iterative self-documenting process by all members of the research team, with planning assistance from the *AIT-CTSI Informatics Core*. The iterative development and testing process results in a well-planned data collection strategy for individual studies."

16.3.3. The CTSI states that regarding security, "REDCap servers are housed in a local data center at the University of Rochester and all web-based information transmission is encrypted. REDCap was developed in a manner consistent with HIPAA security requirements and is recommended to University of Rochester researchers by the URMC Research Privacy Officer and Office for Human Subject Protection.

16.4. Data Storage:

All written materials will be kept confidential, locked in the private offices and limited-access file room of the URCC NCORP Research Base and identified by ID numbers. All electronic information will be kept confidential with password-protected, limited access.

The Case Summary should accompany <u>ALL</u> data submissions. All completed forms and audio-recordings must be submitted within 7 days of the study visit and should be sent to:

Libby Nagalski
URCC NCORP Research Base
Saunders Research Building
265 Crittenden Blvd
Box CU 420658
Rochester, NY 14642

17. DATA COLLECTION AND MEASURES

For a detailed description of the measures that will be collected, refer to **Appendix X-1: Measures**.

18. SUBJECT CONSENT AND PEER JUDGMENT

All investigational, FDA, state, federal and institutional regulations concerning informed consent and peer judgment will be fulfilled.

19. RECORD AND DATA RETENTION

Clinical research records are source documents and records, in any form (including, but not limited to, written, electronic, magnetic, and optical records, in addition, scans (x-rays and electrocardiograms) that describe or record the methods, conduct, and/or results of a trial, and the actions taken. Unlike pharmaceutical-sponsored research, under the Terms of the NIH Award, the awardee institution retains ownership of the clinical research records that were conducted with NIH support. Records may be preserved in hardcopy, electronic or other media form since there is no regulatory requirement that clinical research records be retained in a certain type of format. However, investigators should check with their institution for institutional policies and procedures pertaining to record retention. All records relating to research that is conducted must be retained for at least five years after completion of the research. The three-year time period begins when the individual institution's engagement in the human subjects research activity ends. Human subject research activities are considered completed once all research-related interventions and interactions with human subjects have been completed, all data collection and analysis of identifiable private information described in the IRB-approved research plan have been finished and primary analysis of either identifiable private or de-identified information is completed.

20. REFERENCES

1. Hurria A, Togawa K, Mohile SG, et al. Predicting chemotherapy toxicity in older adults with cancer: a prospective multicenter study. *Journal of clinical oncology : official journal of the American Society of Clinical Oncology.* Sep 1 2011;29(25):3457-3465.
2. Rodin MB, Mohile SG. A practical approach to geriatric assessment in oncology. *Journal of clinical oncology : official journal of the American Society of Clinical Oncology.* May 10 2007;25(14):1936-1944.
3. Mohile S, Dale W, Hurria A. Geriatric oncology research to improve clinical care. *Nature reviews. Clinical oncology.* Jul 24 2012;9(10):571-578.
4. Rao AV, Hsieh F, Feussner JR, Cohen HJ. Geriatric evaluation and management units in the care of the frail elderly cancer patient. *J Gerontol A Biol Sci Med Sci.* Jun 2005;60(6):798-803.
5. Caillet P, Canoui-Poitrine F, Vouriot J, et al. Comprehensive geriatric assessment in the decision-making process in elderly patients with cancer: ELCAPA study. *J Clin Oncol.* Sep 20 2011;29(27):3636-3642.
6. Ingram SS, Seo PH, Martell RE, et al. Comprehensive assessment of the elderly cancer patient: the feasibility of self-report methodology. *J Clin Oncol.* Feb 1 2002;20(3):770-775.
7. Hurria A, Gupta S, Zauderer M, et al. Developing a cancer-specific geriatric assessment: a feasibility study. *Cancer.* Nov 1 2005;104(9):1998-2005.
8. Dale W, Mohile SG, Eldadah BA, et al. Biological, clinical, and psychosocial correlates at the interface of cancer and aging research. *J Natl Cancer Inst.* Apr 18 2012;104(8):581-589.
9. Hurria A, Balducci L, Naeim A, et al. Mentoring junior faculty in geriatric oncology: report from the Cancer and Aging Research Group. *J Clin Oncol.* Jul 1 2008;26(19):3125-3127.
10. Hurria A, Mohile SG, Dale W. Research priorities in geriatric oncology: addressing the needs of an aging population. *J Natl Compr Canc Netw.* Feb 2012;10(2):286-288.
11. Jemal A, Siegel R, Xu J, Ward E. Cancer statistics, 2010. *CA Cancer J Clin.* Sep-Oct;60(5):277-300.
12. Smith BD, Smith GL, Hurria A, Hortobagyi GN, Buchholz TA. Future of Cancer Incidence in the United States: Burdens Upon an Aging, Changing Nation. *Journal of Clinical Oncology.* June 10, 2009 2009;27(17):2758-2765.
13. Dale W, Mohile SG, Eldadah BA, et al. Biological, clinical, and psychosocial correlates at the interface of cancer and aging research. *J Natl Cancer Inst.* Apr 18 2012;104(8):581-589.
14. Mohile S, Dale W, Hurria A. Geriatric oncology research to improve clinical care. *Nature reviews. Clinical oncology.* Jul 24 2012.
15. Mohile SG, Xian Y, Dale W, et al. Association of a cancer diagnosis with vulnerability and frailty in older Medicare beneficiaries. *Journal of the National Cancer Institute.* Sep 2 2009;101(17):1206-1215.
16. Mohile SG, Fan L, Reeve E, et al. Association of cancer with geriatric syndromes in older Medicare beneficiaries. *J Clin Oncol.* Apr 10 2011;29(11):1458-1464.
17. Crome P, Flanagan RJ. Pharmacokinetic studies in elderly people. Are they necessary? *Clin Pharmacokinet.* Apr 1994;26(4):243-247.
18. Fried TR, Bradley EH, Towle VR, Allore H. Understanding the treatment preferences of seriously ill patients. *The New England journal of medicine.* Apr 4 2002;346(14):1061-1066.
19. Keating NL, Norredam M, Landrum MB, Huskamp HA, Meara E. Physical and mental health status of older long-term cancer survivors. *J Am Geriatr Soc.* Dec 2005;53(12):2145-2152.
20. Maione P, Perrone F, Gallo C, et al. Pretreatment quality of life and functional status assessment significantly predict survival of elderly patients with advanced non-small-cell lung cancer receiving chemotherapy: a prognostic analysis of the multicenter Italian lung cancer in the elderly study. *Journal of clinical oncology : official journal of the American Society of Clinical Oncology.* Oct 1 2005;23(28):6865-6872.

21. Stafford RS, Cyr PL. The impact of cancer on the physical function of the elderly and their utilization of health care. *Cancer.* Nov 15 1997;80(10):1973-1980.

22. Audisio RA, Gennari R, Sunouchi K, et al. Preoperative assessment of cancer in elderly patients: a pilot study. *Supportive cancer therapy.* Oct 1 2003;1(1):55-60.

23. Guralnik JM, Simonsick EM, Ferrucci L, et al. A short physical performance battery assessing lower extremity function: association with self-reported disability and prediction of mortality and nursing home admission. *Journal of gerontology.* Mar 1994;49(2):M85-94.

24. Bylow K, Hemmerich J, Mohile SG, Stadler WM, Sajid S, Dale W. Obese frailty, physical performance deficits, and falls in older men with biochemical recurrence of prostate cancer on androgen deprivation therapy: a case-control study. *Urology.* Apr 2011;77(4):934-940.

25. Stone CA, Lawlor PG, Savva GM, Bennett K, Kenny RA. Prospective study of falls and risk factors for falls in adults with advanced cancer. *J Clin Oncol.* Jun 10 2012;30(17):2128-2133.

26. Extermann M, Balducci L, Lyman GH. What threshold for adjuvant therapy in older breast cancer patients? *J Clin Oncol.* Apr 2000;18(8):1709-1717.

27. Frasci G, Lorusso V, Panza N, et al. Gemcitabine plus vinorelbine versus vinorelbine alone in elderly patients with advanced non-small-cell lung cancer. *J Clin Oncol.* Jul 2000;18(13):2529-2536.

28. Firat S, Byhardt RW, Gore E. Comorbidity and Karnofksy performance score are independent prognostic factors in stage III non-small-cell lung cancer: an institutional analysis of patients treated on four RTOG studies. Radiation Therapy Oncology Group. *Int J Radiat Oncol Biol Phys.* Oct 1 2002;54(2):357-364.

29. Piccirillo JF, Tierney RM, Costas I, Grove L, Spitznagel EL, Jr. Prognostic importance of comorbidity in a hospital-based cancer registry. *JAMA.* May 26 2004;291(20):2441-2447.

30. Satariano WA, Ragland DR. The effect of comorbidity on 3-year survival of women with primary breast cancer. *Ann Intern Med.* Jan 15 1994;120(2):104-110.

31. Gronberg BH, Sundstrom S, Kaasa S, et al. Influence of comorbidity on survival, toxicity and health-related quality of life in patients with advanced non-small-cell lung cancer receiving platinum-doublet chemotherapy. *Eur J Cancer.* Aug 2010;46(12):2225-2234.

32. Jung SY, Rosenzweig M, Linkov F, Brufsky A, Weissfeld JL, Sereika SM. Comorbidity as a Mediator of Survival Disparity Between Younger and Older Women Diagnosed With Metastatic Breast Cancer. *Hypertension.* Dec 19 2011.

33. Albertsen PC, Moore DF, Shih W, Lin Y, Li H, Lu-Yao GL. Impact of comorbidity on survival among men with localized prostate cancer. *J Clin Oncol.* Apr 1 2011;29(10):1335-1341.

34. Steyerberg EW, Neville BA, Koppert LB, et al. Surgical mortality in patients with esophageal cancer: development and validation of a simple risk score. *J Clin Oncol.* Sep 10 2006;24(26):4277-4284.

35. Maggiore RJ, Gross CP, Hurria A. Polypharmacy in older adults with cancer. *Oncologist.* 2010;15(5):507-522.

36. Landi F, Zuccala G, Gambassi G, et al. Body mass index and mortality among older people living in the community. *J Am Geriatr Soc.* Sep 1999;47(9):1072-1076.

37. Dewys WD, Begg C, Lavin PT, et al. Prognostic effect of weight loss prior to chemotherapy in cancer patients. Eastern Cooperative Oncology Group. *Am J Med.* Oct 1980;69(4):491-497.

38. Wadley VG, Okonkwo O, Crowe M, Ross-Meadows LA. Mild cognitive impairment and everyday function: evidence of reduced speed in performing instrumental activities of daily living. *Am J Geriatr Psychiatry.* May 2008;16(5):416-424.

39. Sauvaget C, Yamada M, Fujiwara S, Sasaki H, Mimori Y. Dementia as a predictor of functional disability: a four-year follow-up study. *Gerontology.* Jul-Aug 2002;48(4):226-233.

40. Dodge HH, Kadowaki T, Hayakawa T, Yamakawa M, Sekikawa A, Ueshima H. Cognitive impairment as a strong predictor of incident disability in specific ADL-IADL tasks among community-dwelling elders: the Azuchi Study. *The Gerontologist.* Apr 2005;45(2):222-230.

41. Hurria A, Li D, Hansen K, et al. Distress in older patients with cancer. *J Clin Oncol.* Sep 10 2009;27(26):4346-4351.

42. Seeman TE, Berkman LF, Kohout F, Lacroix A, Glynn R, Blazer D. Intercommunity variations in the association between social ties and mortality in the elderly. A comparative analysis of three communities. *Ann Epidemiol.* Jul 1993;3(4):325-335.

43. Kroenke CH, Kubzansky LD, Schernhammer ES, Holmes MD, Kawachi I. Social networks, social support, and survival after breast cancer diagnosis. *J Clin Oncol.* Mar 1 2006;24(7):1105-1111.

44. Kornblith AB, Herndon JE, 2nd, Zuckerman E, et al. Social support as a buffer to the psychological impact of stressful life events in women with breast cancer. *Cancer.* Jan 15 2001;91(2):443-454.

45. Extermann M, Overcash J, Lyman GH, Parr J, Balducci L. Comorbidity and functional status are independent in older cancer patients. *J Clin Oncol.* Apr 1998;16(4):1582-1587.

46. Rockwood K, Stadnyk K, MacKnight C, McDowell I, Hebert R, Hogan DB. A brief clinical instrument to classify frailty in elderly people. *Lancet.* Jan 16 1999;353(9148):205-206.

47. Reuben DB, Rubenstein LV, Hirsch SH, Hays RD. Value of functional status as a predictor of mortality: results of a prospective study. *Am J Med.* Dec 1992;93(6):663-669.

48. Dale W. "Staging the aging" when considering androgen deprivation therapy for older men with prostate cancer. *Journal of clinical oncology : official journal of the American Society of Clinical Oncology.* Jul 20 2009;27(21):3420-3422.

49. Extermann M, Hurria A. Comprehensive geriatric assessment for older patients with cancer. *Journal of clinical oncology : official journal of the American Society of Clinical Oncology.* May 10 2007;25(14):1824-1831.

50. Hurria A, Lichtman SM, Gardes J, et al. Identifying vulnerable older adults with cancer: integrating geriatric assessment into oncology practice. *Journal of the American Geriatrics Society.* Oct 2007;55(10):1604-1608.

51. Repetto L, Fratino L, Audisio RA, et al. Comprehensive geriatric assessment adds information to Eastern Cooperative Oncology Group performance status in elderly cancer patients: an Italian Group for Geriatric Oncology Study. *J Clin Oncol.* Jan 15 2002;20(2):494-502.

52. Kanesvaran R, Li H, Koo KN, Poon D. Analysis of Prognostic Factors of Comprehensive Geriatric Assessment and Development of a Clinical Scoring System in Elderly Asian Patients With Cancer. *J Clin Oncol.* Aug 22 2011.

53. Horgan AM, Leighl NB, Coate L, et al. Impact and Feasibility of a Comprehensive Geriatric Assessment in the Oncology Setting: A Pilot Study. *Am J Clin Oncol.* Mar 17 2011.

54. Hurria A, Cirrincione CT, Muss HB, et al. Implementing a geriatric assessment in cooperative group clinical cancer trials: CALGB 360401. *Journal of clinical oncology : official journal of the American Society of Clinical Oncology.* Apr 1 2011;29(10):1290-1296.

55. Hurria A, Cohen HJ, Extermann M. Geriatric Oncology Research in the Cooperative Groups: A Report of a SIOG Special Meeting. *Journal of geriatric oncology.* Jun 1 2010;1(1):40-44.

56. Pal SK, Katheria V, Hurria A. Evaluating the older patient with cancer: understanding frailty and the geriatric assessment. *CA: a cancer journal for clinicians.* Mar-Apr 2010;60(2):120-132.

57. Detmar SB, Muller MJ, Schornagel JH, Wever LD, Aaronson NK. Health-related quality-of-life assessments and patient-physician communication: a randomized controlled trial. *JAMA : the journal of the American Medical Association.* Dec 18 2002;288(23):3027-3034.

58. Detmar SB, Muller MJ, Schornagel JH, Wever LD, Aaronson NK. Role of health-related quality of life in palliative chemotherapy treatment decisions. *Journal of clinical oncology : official journal of the American Society of Clinical Oncology.* Feb 15 2002;20(4):1056-1062.
59. Weeks JC, Catalano PJ, Cronin A, et al. Patients' expectations about effects of chemotherapy for advanced cancer. *The New England journal of medicine.* Oct 25 2012;367(17):1616-1625.
60. Temel JS, Greer JA, Muzikansky A, et al. Early palliative care for patients with metastatic non-small-cell lung cancer. *N Engl J Med.* Aug 19 2010;363(8):733-742.
61. Cohen HJ, Feussner JR, Weinberger M, et al. A controlled trial of inpatient and outpatient geriatric evaluation and management. *N Engl J Med.* Mar 21 2002;346(12):905-912.
62. Stuck AE, Siu AL, Wieland GD, Adams J, Rubenstein LZ. Comprehensive geriatric assessment: a meta-analysis of controlled trials. *Lancet.* Oct 23 1993;342(8878):1032-1036.
63. Stuck AE, Aronow HU, Steiner A, et al. A trial of annual in-home comprehensive geriatric assessments for elderly people living in the community. *N Engl J Med.* Nov 2 1995;333(18):1184-1189.
64. McCorkle R, Strumpf NE, Nuamah IF, et al. A specialized home care intervention improves survival among older post-surgical cancer patients. *J Am Geriatr Soc.* Dec 2000;48(12):1707-1713.
65. Goodwin JS, Satish S, Anderson ET, Nattinger AB, Freeman JL. Effect of nurse case management on the treatment of older women with breast cancer. *J Am Geriatr Soc.* Sep 2003;51(9):1252-1259.
66. Seymour MT, Thompson LC, Wasan HS, et al. Chemotherapy options in elderly and frail patients with metastatic colorectal cancer (MRC FOCUS2): an open-label, randomised factorial trial. *Lancet.* May 21 2011;377(9779):1749-1759.
67. Arora NK, Street RL, Jr., Epstein RM, Butow PN. Facilitating patient-centered cancer communication: a road map. *Patient education and counseling.* Dec 2009;77(3):319-321.
68. Shields CG, Coker CJ, Poulsen SS, et al. Patient-centered communication and prognosis discussions with cancer patients. *Patient education and counseling.* Dec 2009;77(3):437-442.
69. Street RL, Jr., Makoul G, Arora NK, Epstein RM. How does communication heal? Pathways linking clinician-patient communication to health outcomes. *Patient education and counseling.* Mar 2009;74(3):295-301.
70. Street RL, Jr., Elwyn G, Epstein RM. Patient preferences and healthcare outcomes: an ecological perspective. *Expert review of pharmacoeconomics & outcomes research.* Apr 2012;12(2):167-180.
71. Andreozzi J, Dale W, Fan L, Mohile SG. Feasibility of Incorporting Geriatric Assessment into Oncology Practice in Real-Time. *American Geriatrics Society National Meeting.* 2011(D104).
72. Guigoz Y. The Mini Nutritional Assessment (MNA) review of the literature--What does it tell us? *The journal of nutrition, health & aging.* Nov-Dec 2006;10(6):466-485; discussion 485-467.
73. Yesavage JA, Brink TL, Rose TL, et al. Development and validation of a geriatric depression screening scale: a preliminary report. *Journal of psychiatric research.* 1982;17(1):37-49.
74. Katzman R, Brown T, Fuld P, Peck A, Schechter R, Schimmel H. Validation of a short Orientation-Memory-Concentration Test of cognitive impairment. *The American journal of psychiatry.* Jun 1983;140(6):734-739.
75. Guralnik JM, Ferrucci L, Simonsick EM, Salive ME, Wallace RB. Lower-extremity function in persons over the age of 70 years as a predictor of subsequent disability. *The New England journal of medicine.* Mar 2 1995;332(9):556-561.
76. Epstein RM, Franks P, Fiscella K, et al. Measuring patient-centered communication in patient-physician consultations: theoretical and practical issues. *Social science & medicine.* Oct 2005;61(7):1516-1528.

77. Epstein RM, Alper BS, Quill TE. Communicating evidence for participatory decision making. *JAMA.* May 19 2004;291(19):2359-2366.

78. Epstein RM, Street RL, Jr. Shared mind: communication, decision making, and autonomy in serious illness. *Annals of family medicine.* Sep-Oct 2011;9(5):454-461.

79. Young HN, Bell RA, Epstein RM, Feldman MD, Kravitz RL. Physicians' shared decision-making behaviors in depression care. *Arch Intern Med.* Jul 14 2008;168(13):1404-1408.

80. Dale W, Hemmerich J, Bylow K, Mohile S, Mullaney M, Stadler WM. Patient anxiety about prostate cancer independently predicts early initiation of androgen deprivation therapy for biochemical cancer recurrence in older men: a prospective cohort study. *Journal of clinical oncology : official journal of the American Society of Clinical Oncology.* Apr 1 2009;27(10):1557-1563.

81. Velikova G, Booth L, Smith AB, et al. Measuring quality of life in routine oncology practice improves communication and patient well-being: a randomized controlled trial. *J Clin Oncol.* Feb 15 2004;22(4):714-724.

82. Williams GC, Deci EL. The importance of supporting autonomy in medical education. *Annals of internal medicine.* Aug 15 1998;129(4):303-308.

83. Williams GC, McGregor HA, Sharp D, et al. Testing a self-determination theory intervention for motivating tobacco cessation: supporting autonomy and competence in a clinical trial. *Health psychology : official journal of the Division of Health Psychology, American Psychological Association.* Jan 2006;25(1):91-101.

84. Vallerand RJ, O'Connor BP, Blais MR. Life satisfaction of elderly individuals in regular community housing, in low-cost community housing, and high and low self-determination nursing homes. *International journal of aging & human development.* 1989;28(4):277-283.

85. Fiscella K, Franks P, Srinivasan M, Kravitz RL, Epstein R. Ratings of physician communication by real and standardized patients. *Annals of family medicine.* Mar-Apr 2007;5(2):151-158.

86. Zafar SY, Malin JL, Grambow SC, et al. Chemotherapy use and patient treatment preferences in advanced colorectal cancer: a prospective cohort study. *Cancer.* Feb 15 2013;119(4):854-862.

87. Safran DG, Karp M, Coltin K, et al. Measuring patients' experiences with individual primary care physicians. Results of a statewide demonstration project. *Journal of general internal medicine.* Jan 2006;21(1):13-21.

88. Press Ganey: public reporting gives huge boost to patient satisfaction. *Healthcare benchmarks and quality improvement.* Dec 2008;15(12):121-123.

89. Cowan P. Press Ganey scores and patient satisfaction in the emergency department (ED): the patient perspective. *Pain medicine.* Jul 2013;14(7):969.

90. Zusman EE. HCAHPS replaces Press Ganey survey as quality measure for patient hospital experience. *Neurosurgery.* Aug 2012;71(2):N21-24.

91. Hoerger M, Epstein RM, Winters PC, et al. Values and options in cancer care (VOICE): study design and rationale for a patient-centered communication and decision-making intervention for physicians, patients with advanced cancer, and their caregivers. *BMC cancer.* 2013;13:188.

92. http://www.hcahpsonline.org. Centers for Medicare & Medicaid Services, Baltimore, MD.

93. Reid RJ, Coleman K, Johnson EA, et al. The Group Health medical home at year two: cost savings, higher patient satisfaction, and less burnout for providers. *Health affairs.* May 2010;29(5):835-843.

94. Grunfeld E, Fitzpatrick R, Mant D, et al. Comparison of breast cancer patient satisfaction with follow-up in primary care versus specialist care: results from a randomized controlled trial. *Brit J Gen Pract.* Sep 1999;49(446):705-710.

95. Morris BJ, Richards JE, Archer KR, et al. Improving Patient Satisfaction in the Orthopaedic Trauma Population. *Journal of orthopaedic trauma.* Oct 28 2013.

96. Clayton JM, Butow PN, Tattersall MH, et al. Randomized controlled trial of a prompt list to help advanced cancer patients and their caregivers to ask questions about prognosis and end-of-life care. *J Clin Oncol.* Feb 20 2007;25(6):715-723.
97. Butow P, Cockburn J, Girgis A, et al. Increasing oncologists' skills in eliciting and responding to emotional cues: evaluation of a communication skills training program. *Psychooncology.* Mar 2008;17(3):209-218.
98. Detmar SB. Use of HRQOL questionnaires to facilitate patient-physician communication. *Expert review of pharmacoeconomics & outcomes research.* Jun 2003;3(3):215-217.
99. Brucker PS, Yost K, Cashy J, Webster K, Cella D. General population and cancer patient norms for the Functional Assessment of Cancer Therapy-General (FACT-G). *Evaluation & the health professions.* Jun 2005;28(2):192-211.
100. Cella DF, Tulsky DS, Gray G, et al. The Functional Assessment of Cancer Therapy scale: development and validation of the general measure. *J Clin Oncol.* Mar 1993;11(3):570-579.
101. Temel JS, Jackson VA, Billings JA, et al. Phase II study: integrated palliative care in newly diagnosed advanced non-small-cell lung cancer patients. *J Clin Oncol.* Jun 10 2007;25(17):2377-2382.
102. Temel J. Complexities of quality of life analysis in non-small cell lung cancer. *J Support Oncol.* Jan 2007;5(1):30-31.
103. Given CW, Given B, Stommel M, Collins C, King S, Franklin S. The caregiver reaction assessment (CRA) for caregivers to persons with chronic physical and mental impairments. *Res Nurs Health.* Aug 1992;15(4):271-283.
104. Brown H, Prescott R. *Applied Mixed Models in Medicine.* 2nd edition ed. Edinburgh, UK: John Wiley and Sons, Ltd; 2006.
105. Kenward MG, Roger JH. Small sample inference for fixed effects from restricted maximum likelihood. *Biometrics.* Sep 1997;53(3):983-997.
106. Little R, Rubin D. *Stastical analysis with missing data.* 2nd edition ed. Hoboken, NJ: Wiley; 2002.

APPENDIX X-1

MEASURES

MEASURES TO BE COLLECTED

Collection Time-points are Screening/Baseline, Telephone Team Call to patient within 1 to 7 days from baseline to assess patient satisfaction, 4-6 weeks, 10-14 weeks (3 months) and 20-24 weeks (6 months). Measures signified by * are only collected at screening/baseline and not at follow-up visits. Measures signified by *(Follow-up)* are collected only at follow-up visits.**

We have piloted all measures. In total, geriatric assessment measures that are filled out by the patient require approximately 20 minutes of time. The additional measures (quality of life, symptoms, communication, decision-making) captured at baseline require an additional 30 minutes of time. The follow-up questionnaires require about 30 minutes of time in total. Caregiver assessments require about 30 minutes of time.

We have incorporated flexibility with timing in order to reduce patient burden. Patients and caregivers may complete geriatric assessment at clinic at time of consent or before next visit. They may choose to complete measures at home in between visits. We have found that 90% of patients complete measures at home if allowed to do so. The geriatric oncology clinic at the University of Rochester routinely captures these measures as part of clinical care.

The telephone call that will capture the patient satisfaction measures (based on the Health Care Climate Questionnaire, HCCQ) will take <10 minutes and will occur within 1 to 7 days of the baseline audiotaped visit. The assessments performed by the Clinical Research Associate take about 30 to 45 minutes of time in total (including physical performance and cognitive tests). Any person at the practice site can be trained by Research Base staff to do the assessments. The assessments do not need to be performed by the physician.

The physician assessments will be done either on paper or through REDCap, whichever the physician prefers. The baseline assessments take no longer than 10 minutes and after each patient visit, the decision-making form (to assess factors that influenced decisions) is less than one-page long (2 minutes to complete).

1. **Patient Surveys**

   1.1. *Demographics***:* Age, race and ethnicity, gender, highest level of education achieved, employment status, marital status, living situation, and presence of a living companion will be captured. We will also assess financial concerns, understanding of disease, self-rated health, and subjective age.

   1.2. *Geriatric assessment:* Assessment tools comprising the comprehensive geriatric assessment are discussed below. The various assessment tools were selected based upon extensive data in the geriatric literature demonstrating predictive value as well as feasibility data in multiple studies of elderly patients with cancer. Other than the cognitive and physical performance measures, the assessments are self-administered. Patients who cannot complete the assessment on their own can receive assistance from the study personnel or caregiver. The comprehensive assessment is performed first prior to treatment and brief follow-up GA measures are collected at 4-6 weeks, 3 months, and 6 months. Measures collected only at baseline are noted with ***.

   1.2.1. *Activities of daily living (ADL):*[1] ADLs are measures of self-care. ADL independence will be assessed using the Katz Index of Independence in Activities of Daily Living, commonly referred to as the Katz ADL. The Katz ADL is the most appropriate instrument to assess functional status as a measurement of the patient's ability to perform activities of daily living independently. Clinicians typically use the tool to detect problems in performing activities of daily living and to plan care accordingly. The Index ranks adequacy of performance in the six functions

of *bathing, dressing, toileting, transferring, continence, and feeding.* Patients are scored yes/no for independence in each of the six functions. A score of 6 indicates full function, 4 indicates moderate impairment, and 2 or less indicates severe functional impairment.

1.2.2. *Instrumental Activities of Daily Living (IADL):*[2] Self-reported functional status will be assessed using the IADL subscale of the Multidimensional Functional Assessment Questionnaire: Older American Resources and Services (OARS). The IADL subscale consists of seven questions rated on a three-point Likert scale. It measures the degree to which an activity can be performed independently.

1.2.3. *Fall History:* A self-reported history of falls in the past six months will be recorded. A history of a recent fall has been demonstrated to be independently predictive of increased risk for chemotherapy toxicity in older cancer patients.[3]

1.2.4. *OARS Physical Health:*[2] Self-reported questions that assess the degree of difficulty with physical tasks such as walking, climbing stairs, stooping, and reaching. This measure correlates with disability and comorbidity.

1.2.5. *OARS Comorbidity***:*[2] Patients self-report their coexisting medical conditions and also rate the degree to which their illness causes impairment in daily activities. The OARS Physical Health Section has been shown to correlate significantly with health professional ratings of comorbidity as well.

1.2.6. *OARS Medical Social Support and Social Activities:****[2] A 13-question survey asking patients to identify the number of support persons involved in their medical care as well as the degree to which they felt supported in a variety of situations. A follow-up question will be used to assess how much a patient's physical or emotional health interfered with *social activities*.

1.2.7. *Generalized Anxiety Disorder 7 (GAD-7):****[4] The GAD-7 is a self-administered patient questionnaire used as a screening tool and severity measure for generalized anxiety disorder. The GAD-7 score is calculated by assigning scores of 0, 1, 2, and 3, to the response categories of "hardly ever," "several days," "more than half the days," and "nearly every day," respectively, and adding together the scores for the seven questions. Scores of 5, 10, and 15 are taken as the cut off points for mild, moderate, and severe anxiety, respectively. When used as a screening tool, further evaluation is recommended when the score is 10 or greater. Using the threshold score of 10, the GAD-7 has a sensitivity of 89% and a specificity of 82% for generalized anxiety disorder. It is moderately good at screening three other common anxiety disorders – panic disorder (sensitivity 74%, specificity 81%), social anxiety disorder (sensitivity 72%, specificity 80%), and post-traumatic stress disorder (sensitivity 66%, specificity 81%).

1.2.8. *Geriatric Depression Scale (GDS):*[5] Patients will be screened with the Geriatric Depression Scale (GDS). The GDS contains questions that are intended to screen elderly patients for depression, while parsing out complaints related to advanced age.[6]

1.3. *Satisfaction, Quality of Life, and Symptoms:*

1.3.1. *Health Care Climate Questionnaire (HCCQ) (Follow-up)*[7-9] measures patient- centered autonomy-supportive physician behaviors such as whether the patient and caregiver feels that the physician understands his/her perspective, provides choices and options, and encourages participation in decisions. The measure has been studied and validated in older patients. Similar to other studies which adapt satisfaction scales to capture specific clinical criteria (e.g., satisfaction with physician regarding communication about chemotherapy),[10] the HCCQ has been modified for this study to specifically address patient satisfaction with physician behaviors and communication regarding age-related issues and concerns in order to specifically address satisfaction with the

intervention (geriatric assessment summary) rather than satisfaction with other aspects of cancer care (e.g., communication about cancer treatment)(HCCQ-age). As is done with satisfaction with care surveys in other research and in clinical settings, the HCCQ (both modified and original) will be administered within 1 week after the audio-taped clinic visit.[11-14] Our University of Chicago collaborators (Dale and Gorawara-Bhatt) have experience with the conduct of such assessments over the phone and this minimizes perceived or real influence from the physician or team. The University of Chicago collaborators and research staff, who are subcontracted through a PCORI contract with the University of Chicago, will be blinded to group assignment. The HCCQ will also be completed as part of the patient and caregiver packets in follow up time points for comparison.

1.3.2. *Press-Ganey Patient Satisfaction Survey (Follow-up):* The Press Ganey patient satisfaction survey consists of a standardized set of questions that measure patient satisfaction in a given healthcare setting. The survey also represents one of the most widely used methods in the ambulatory setting. Additional questions may be added to the survey in order to assess unique services or performance improvement issues for a specific organization. The Press Ganey survey has been utilized to assess patient-reported outcomes in satisfaction for research studies in a number of areas within healthcare, including emergency and cardiovascular medicine.[15] Measures were selected from the Press Ganey survey for utilization in the current protocol to assess patient satisfaction with their oncology care related to their physician/healthcare provider, personal issues related to their care, experience with chemotherapy, and an overall assessment of their care.

1.3.3. *FACT:* Quality of life will be measured using the Functional Assessment of Chronic Illness Therapy tool. Although there are several validated tools for QoL, the FACT has been validated in the geriatric population.[16,17] It is a subset of a larger group of FACT scales that assess health-related quality of life measures. It has demonstrated high internal validity and high test-retest reliability.[18]

1.3.4. *Karnofsky Self-Reported Performance Status:* This one item question will assess impact of cancer and cancer symptoms on overall health.

1.3.5. *MD Anderson Symptom Inventory (MDASI):* Symptoms will be assessed using the MD-Anderson symptom inventory (MDASI) tool. This is a brief patient-reported measure assessing the impact of cancer-related symptoms. This 19-question survey assesses 13 core symptom items found to have the highest frequency and/or severity in cancer patients. It also includes items relating to symptom interference with daily life. Studies have demonstrated good internal reliability.[19]

1.3.6. *Emotional Distress:* Different domains of emotional distress will be assessed. The Distress Thermometer is widely used by health professionals to assess the level of patient distress (on a 0-10 scale).[20] Dr. Hurria found that 41% of older patients with cancer (n=245) report significant distress and found an association between higher distress ($\geq$ 4) and poorer physical function, increased comorbid medical conditions, and poor eyesight.[21] Perceived burdensomeness was assessed using one item from the Interpersonal Needs Questionnaire- Revised (INQ-R).[22] Okuyama et al.'s study of cancer patients' reluctance to disclose their emotional distress to their physician assesses 4 domains of emotional distress: no perceived need, fear of negative impact, negative attitude, and hesitation using the instrument, Reluctance to Disclose Emotional Distress (RDED).[23]

1.4. **Communication and Decision-Making Preferences**

1.4.1. *Peace, Equanimity, and Acceptance in the Cancer Experience (PEACE)****[24] evaluates the extent to which patients with advanced cancer have a sense of peaceful acceptance of their terminal illness. Evaluated in 160 patients with advanced cancer, the 12-item PEACE

questionnaire has 2 subscales: a 7-item Struggle With Illness subscale (Cronbach's alpha = .81) and a 5-item Peaceful Acceptance subscale (alpha = 0.78). Both subscales were associated with patients' self-reported peacefulness (correlation coefficient [r] = 0.66 for acceptance [P <0.01]; r = -0.37 for struggle [P <0 .01]). This measure has been adapted for use with caregivers through Dr. Epstein's work.

1.4.2. *The Perceived Efficacy in Patient-Physician Interactions (PEPPI) scale****[25] measures patients' and caregivers' confidence in their ability to communicate their concerns, obtain and understand information, ask questions, clarify uncertainties, and make sure that their doctor understands them. In older patients, a 5-item short form of the PEPPI demonstrated Cronbach's alphas of 0.83. PEPPI demonstrated discriminant and convergent validity, correlating positively with active coping (r=0.17, P=0.03), and with patient satisfaction with physician interpersonal manner (r=0.49, P < 0.01) and communication (r=0.51, P < 0.01).

1.4.3. *Control Preferences Scale****[26] assesses whether patients and caregivers would want an active, passive, or shared decision-making process with their doctors. This tool has been validated for use in advanced cancer patients, older patients, and caregivers.[27,28]

1.4.4. *MUIS-Complexity Subscale:*[29] Complexity is one of four validated subscales of the MUIS that addresses cues about treatment and system of care that are multiple and varied.

2. CRA Packet (CRA fills out at visits)

2.1. *Tumor and Treatment Characteristics:* The tumor stage, previous surgery or radiation, chemotherapy type, dosing, and schedule (intended and received), other cancer treatments, and supportive care medications will be captured by the CRA. Survival status at 12 months from study entry will be captured on the withdrawal form or survival status form, once available.

2.1.1. *Cancer Treatment History* will be used to collect the patient's previous treatments for his/her advanced cancer.

2.2. *KPS Physician Rated:* The CRA will obtain the physician's assessment of the impact of cancer and cancer treatment on the patient's overall function.

2.3. Geriatric Assessment

2.3.1. *Timed Up and Go***:*[30] The Timed Up & Go is a performance based test of functional status, measuring how many seconds it takes to stand up from a standard arm-chair, walk 3 meters (10 feet), turn, walk back to the chair, and sit down again. In community dwelling older adults, there was inter-rater and intra-rater reliability (intra-class correlation coefficient 0.99 for both).

2.3.2. *Mini-Cog:* A tool that is validated in the geriatric population to quickly assess cognitive impairment.[31,32] The Mini-Cog takes approximately 3 minutes to administer. It has minimal language content, which reduces cultural and educational bias. It combines a 3-item recall component with a Clock Drawing Test.

2.3.3. *Short Blessed Orientation-Memory-Concentration (BOMC) Test ***:* A six-question evaluation that screens for cognitive impairment. Studies have shown its validity as a screening instrument and the correlation of its results with those of more extensive mental status tests.[33]

2.3.4. *Nutritional Status and Mini Nutrition Assessment (MNA):* Screening for nutritional deficit will be performed with body mass index (BMI) evaluation and self-reported weight loss. Further nutritional evaluation will be performed with *the Mini-Nutritional*

*Assessment*** (MNA)*[34], a well validated screening measure for nutritional deficiency which has shown to be prognostic of survival in older patients with cancer. Weight will be assessed at each time point. Height will be measured at baseline.

2.3.5. *Short Physical Performance Battery:*[35] Physical performance measures objectively evaluate mobility and fall risk. Falls are common in older cancer patients and predictive of adverse outcomes.

2.3.6. *Labs:* CRA will send results of baseline tests collected including hemoglobin, liver function tests, and renal function.

2.3.7. *Polypharmacy* will be ascertained from the medical record after patients have been asked to review their medication list on file for any changes in the *Polypharmacy Log and Polypharmacy High Risk Drug Review*.

3. Caregiver Packet

3.1. *Demographics***:* Age, race and ethnicity, gender, highest level of education achieved, employment status, marital status, and presence of a living companion will be captured. Additionally, we will collect information on underlying health conditions (*Physical Health)*.

3.2. *Caregiver Reaction Assessment (CRA)*[36] is designed to measure the reactions of family members to caring for elderly relatives. The instrument consists of five dimensions (caregiver's esteem, lack of family support, finances, schedule, and health). Items are rated on a 5-point scale (from "strongly agree" to "strongly disagree"). The CRA allows for measurement of positive and negative reactions to caregiving.

3.3. *The 12-Item Short Form Health Survey (SF-12):*[37] This measure was developed for the Medical Outcomes Study (MOS), a multi-year study of patients with chronic conditions. The measure assesses functional health and well-being.

3.4. *Cost of Care*:[38] Hanly et al. conducted a survey that identified indirect and directs costs associated with taking care of a cancer patient.

3.5. *Ryff measure of psychological well-being:*[39] The Ryff 6-construct assessment of well-being has been studied within numerous settings. The environmental mastery subscale will be used to assess how well the caregiver perceives that he/she is managing responsibilities.

3.6. Psychological well-being: The caregiver psychological wellbeing will be assessed with two measures, *PHQ2 and GAD-7****. Caregiver's perspective on patients' psychological well-being will be captured with AD-8.[40]

3.7. Caregivers will also complete 1.2.5., 1.3.1., 1.3.2., 1.3.6., and measures within 1.4.

4. Physician Assessment

4.1. *Physician Baseline Demographics and Treatment Preferences***:* Age, race and ethnicity, gender, and details on medical practice will be captured. We will also capture patient volume, and specify years of training after fellowship. We will assess comfort with shared decision making in the baseline survey. The goal of shared decision- making is to make decisions in a manner consistent with the patient's wishes. The patient drives the process. Determining where on the shared decision-making continuum the patient feels most comfortable requires clear communication and dedicated time from the physician. Several studies have utilized the proposed measure for assessing the relationship of physician decision-making styles on clinical outcomes.[28,41,42]

4.2. *Situational Vignettes***:* Physicians will be presented with one of eight clinical scenarios of an elderly cancer patient with a variety of geriatric-related impairments (i.e. physical frailty, cognitive impairment). A series of questions will follow each vignette inquiring about the likelihood of the physician to offer chemotherapy in the scenario and details regarding the regimen that would be considered (i.e. chemotherapy type, dosing, etc.). Only one vignette will be provided to each physician. The survey will not be repeated with each subsequent patient.

Treatment Decision-Making Form (after each audiotaped visit): Physicians will complete a short (<10 questions) follow-up survey requesting information on the treatment plan for the patient and factors that influenced how the decision was made. This follow-up survey is adapted from work by Dr. Dale and Dr. Mohile evaluating how decisions are made for starting hormonal treatment for prostate cancer. Physicians will be asked to identify factors that influenced their decision in developing a treatment plan for each specific patient (i.e., age, stage of disease, performance status, geriatric measures). Physicians will rank each factor to determine which are most influential in their decision making process. Physicians will also be asked if results of geriatric assessment influenced their decision-making. If physicians have multiple patients enrolled on study, this survey will be completed for each individual patient.

4.3. *Physician Follow-up Survey (follow-up):* Physicians will complete a brief survey on REDCap, which will ask them about confidence in geriatrics and their opinion on the usefulness of the Geriatric Assessment (for intervention arm). Some questions asked at baseline will be repeated at study completion.

4.4. *Understanding of Disease-Physician* [a]*:* Measures what the physician believes about the patient's illness.

5. **Audio-recordings of oncologist-patient visit**

A CRA will audio-record the patient-oncologist consultation. This visit must occur after the geriatric assessment is completed and before treatment initiation. A medical consultation should be scheduled prior to start of cancer treatment (if planned). We will assess the number of age-related concerns brought up by patients and caregivers. We will also assess how the physician addresses these concerns. Our team has experience with all of the study measures. **Transcriptionists** will transcribe all audio-recorded visits and will be blinded to study condition. **Coders** will undergo extensive training and supervision by developers of the scales. Transcriptionists and coders will not be part of the study team or involved in any other aspects of the study, and will be blinded to study hypotheses and site assignments to intervention or control. Further, during analysis, study team members will be blinded to site assignments of intervention or control.

TABLES OF DATA TO BE KEPT

Table 1: Patient Measures

**Measure**	**Aim**	**Screening Visit 00**	**Baseline Visit 01**	**Telephone Team Call[a]**	**4-6 Weeks Visit 02**	**3 Months Visit 03**	**6 Months Visit 04**
Demographics		Pt					
ADL	GA	Pt			Pt	Pt	Pt
IADL	GA	Pt			Pt	Pt	Pt
Fall History	GA	Pt			Pt (f/u)	Pt (f/u)	Pt (f/u)
OARS Physical Health	GA	Pt			Pt	Pt	Pt
OARS Comorbidity	GA	Pt					
OARS Medical Social Support	GA	Pt					
Social Activities	GA	Pt			Pt (1 item)	Pt (1 item)	Pt (1 item)
GAD-7	GA	Pt					
GDS	GA	Pt			Pt	Pt	Pt
Patient-rated KPS	S2	Pt			Pt	Pt	Pt
HCCQ	P1			Pt	Pt	Pt	Pt
HCCQ-age	P1			Pt	Pt	Pt	Pt
Press-Ganey Pt Satisfaction	P1				Pt	Pt	Pt
FACT	S2		Pt		Pt	Pt	Pt
MDASI	S2		Pt		Pt	Pt	Pt
Emotional Distress	S2	Pt			Pt	Pt	Pt
PEACE	E		Pt		Pt		
PEPPI	E		Pt		Pt		
Control Preferences Scale	E		Pt				
MUIS- Complexity Subscale	E		Pt		Pt	Pt	Pt
Understanding of Disease	E		Pt		Pt		
Survey Completion		Pt			Pt	Pt	Pt

Note: Screening and baseline can be combined. [a] A research staff member from the Telephone Team will call the patient within 1 to 7 days after the baseline audiorecorded visit.

Abbreviations: Pt (Patient); Phys (Physician); GA (Geriatric Assessment); P1 (Primary Aim 1); E (Exploratory Aim); S2 (Secondary Aim 2); ADL (Activities of Daily Living); IADL (Instrumental Activities of Daily Living); GAD (Generalized Anxiety Disorder 7-Item Scale); Geriatric Depression Scale (GDS); KPS (Karnofsky Performance Status); PEACE (Peace, Equanimity, and Acceptance in the Cancer Experience); PEPPI (The Perceived Efficacy in Patient-Physician Interactions); MUIS (Mishel Uncertainty in Illness Scale); CTSQ (Cancer Treatment Satisfaction Questionnaire); FACTF (Functional Assessment of Chronic Illness Therapy); MDASI (MD Anderson Symptom Inventory).

Table 2: Caregiver Measures

Measure	Aim	Screening Visit 00	Baseline Visit 01	Telephone Team Call[a]	4-6 Weeks Visit 02	3 Months Visit 03	6 Months Visit 04
Demographics		C					
Caregiver Reaction	S2	C			C	C	C
OARS Comorbidity	S2	C					
SF-12	S2		C		C (f/u)	C (f/u)	C (f/u)
Cost of Care	S2	C			C	C	C
Ryff Environmental Mastery	S2		C		C	C	C
PHQ-2	S2		C		C	C	C
GAD-7 Anxiety	S2		C				
Health Care Climate Questionnaire-Communication (caregiver)	S3				C	C	C
HCCQ-age (patient)	S3				C	C	C
HCCQ-age (general, caregiver)	S3				C	C	C
Press-Ganey Pt Satisfaction	S3				C	C	C
Distress Thermometer	S2	C			C	C	C
PEACE	E		C		C		
PECPI	E		C		C		
Control Preferences Scale	E		C				
MUIS- Complexity Subscale	E		C		C	C	C
Understanding of Disease	E		C		C		
AD8	E	C			C (f/u)	C (f/u)	C (f/u)

Note: Screening and baseline can be combined. [a] A research staff member from the Telephone Team will call the patient within 1 to 7 days after the baseline audiorecorded visit.

Abbreviations: C (Caregiver); CRA (Clinical Research Associate); Pt (Patient); Phys (Physician); GA (geriatric assessment); P1 (Primary Aim 1); I (Intervention); E (Exploratory Aim); S1 (Secondary Aim 1); S2 (Secondary Aim 2); S3 (Secondary Aim 3); PEACE (Peace, Equanimity, and Acceptance in the Cancer Experience); PEPPI (The Perceived Efficacy in Patient-Physician Interactions); MUIS (Mishel Uncertainty in Illness ScaleSF-12 (12-Item Short Form Health Survey); PHQ-2 (2-Item Patient Health Questionnaire); GAD (Generalized Anxiety Disorder 7-Item Scale); HCCQ (Health Care Climate Questionnaire); KPS (Karnofsky Performance Status); Blessed OMC (Blessed-Orientation Memory Concentration Test); SPPB (Short Physical Performance Battery).

Table 3: Clinical Research Associate & Physician Measures

Measures	Aim	Screening Visit 00	Baseline Visit 01	Telephone Team Call[a]	4-6 Weeks Visit 02	3 Months Visit 03	6 Months Visit 04
Audio-taped Pt-Phys Visit	S1		CRA				
Screening Coversheet page 2[b]		CRA					
Tumor & Treatment Characteristics	E	CRA			CRA (f/u)	CRA (f/u)	CRA (f/u)
Cancer Treatment History Form	S1		CRA				
Physician rated KPS	S2	CRA			CRA	CRA	CRA
Lab Screening Form	GA	CRA					
Polypharmacy	GA	CRA					
Polypharmacy High Risk Drug Review	GA	CRA					
BOMC Test	GA	CRA					
Mini-Cog	GA	CRA			CRA	CRA	CRA
Nutritional Status and MNA	GA	CRA					
Timed "Up and Go"	GA	CRA					
Short Physical Performance Battery	GA	CRA			CRA	CRA	CRA
GA Scoring Guide to Detect Impairments	GA	CRA					
Physician Baseline Survey[c]	E	Phys					
Situational Vignettes[c]	E	Phys					
Physician Follow-Up Survey[d]	E						Phys[d]
Treatment Decision Making Form	E		Phys				
Understanding of Disease -Physician	E		Phys				
**Study Related Forms[e]**							
Screening Log							
Patient Eligibility Screening Form							
Caregiver Eligibility Screening Form							
Patient Withdrawal Form							
Physician Withdrawal Form							
Caregiver Withdrawal Form							
Survival Status Form[f]							
URCC NCORP AE Report							

Note: Screening and baseline can be combined. The measures/forms are not listed in the order of administration. [a] A research staff member from the Telephone Team will call the patient within 1 to 7 days after the baseline audiorecorded visit. [b] The Screening Coversheet page 2 collects patient information that will be used to establish survival status. [c] The Physican Baseline Survey will be administered via REDCap or paper form and the situational vignettes are collected as part of the Physician Baseline Survey. **[d]** The physician follow-up survey will be administered at the end of the study period. [e] These forms will be used for study documentation purposes. [f]Collected at one year. **Abbreviations:** C (Caregiver); CRA (Clinical Research Associate); Pt (Patient); Phys (Physician); GA (geriatric assessment); P1 (Primary Aim 1); I (Intervention); E (Exploratory Aim); S1 (Secondary Aim 1); S2 (Secondary Aim 2); S3 (Secondary Aim 3); KPS (Karnofsky Performance Status); Blessed OMC (Blessed-Orientation Memory Concentration Test); SPPB (Short Physical Performance Battery),NCI: National Cancer Institute; URCC NCORP Research Base (University of Rochester Cancer Center NCI Community Oncology Research Program Research Base), AE (Adverse Event).

MEASURES REFERENCES:

1. Katz S, Downs TD, Cash HR, Grotz RC. Progress in development of the index of ADL. The Gerontologist 1970;10:20-30.
2. Hurria A, Gupta S, Zauderer M, et al. Developing a cancer-specific geriatric assessment: a feasibility study. Cancer 2005;104:1998-2005.
3. Hurria A, Togawa K, Mohile SG, et al. Predicting chemotherapy toxicity in older adults with cancer: a prospective multicenter study. Journal of clinical oncology : official journal of the American Society of Clinical Oncology 2011;29:3457-65.
4. Spitzer RL, Kroenke K, Williams JB, Lowe B. A brief measure for assessing generalized anxiety disorder: the GAD-7. Archives of internal medicine 2006;166:1092-7.
5. Parmelee PA, Katz IR. Geriatric depression scale. Journal of the American Geriatrics Society 1990;38:1379.
6. Adams KB, Matto HC, Sanders S. Confirmatory factor analysis of the geriatric depression scale. The Gerontologist 2004;44:818-26.
7. Williams GC, Deci EL. The importance of supporting autonomy in medical education. Annals of internal medicine 1998;129:303-8.
8. Williams GC, McGregor HA, Sharp D, et al. Testing a self-determination theory intervention for motivating tobacco cessation: supporting autonomy and competence in a clinical trial. Health psychology : official journal of the Division of Health Psychology, American Psychological Association 2006;25:91-101.
9. Vallerand RJ, O'Connor BP, Blais MR. Life satisfaction of elderly individuals in regular community housing, in low-cost community housing, and high and low self-determination nursing homes. International journal of aging & human development 1989;28:277-83.
10. Zafar SY, Malin JL, Grambow SC, et al. Chemotherapy use and patient treatment preferences in advanced colorectal cancer: a prospective cohort study. Cancer 2013;119:854-62.
11. Safran DG, Karp M, Coltin K, et al. Measuring patients' experiences with individual primary care physicians. Results of a statewide demonstration project. Journal of general internal medicine 2006;21:13-21.
12. Press Ganey: public reporting gives huge boost to patient satisfaction. Healthcare benchmarks and quality improvement 2008;15:121-3.
13. Cowan P. Press Ganey scores and patient satisfaction in the emergency department (ED): the patient perspective. Pain medicine 2013;14:969.
14. Zusman EE. HCAHPS replaces Press Ganey survey as quality measure for patient hospital experience. Neurosurgery 2012;71:N21-4.
15. Glickman SW, Boulding W, Manary M, et al. Patient satisfaction and its relationship with clinical quality and inpatient mortality in acute myocardial infarction. Circulation Cardiovascular quality and outcomes 2010;3:188-95.
16. Webster K, Cella D, Yost K. The Functional Assessment of Chronic Illness Therapy (FACIT) Measurement System: properties, applications, and interpretation. Health and quality of life outcomes 2003;1:79.
17. Avlund K, Pedersen AN, Schroll M. Functional decline from age 80 to 85: influence of preceding changes in tiredness in daily activities. Psychosomatic medicine 2003;65:771-7.
18. Chandran V, Bhella S, Schentag C, Gladman DD. Functional assessment of chronic illness therapy-fatigue scale is valid in patients with psoriatic arthritis. Annals of the rheumatic diseases 2007;66:936-9.
19. Cleeland CS, Mendoza TR, Wang XS, et al. Assessing symptom distress in cancer patients: the M.D. Anderson Symptom Inventory. Cancer 2000;89:1634-46.
20. Ransom S, Jacobsen PB, Booth-Jones M. Validation of the Distress Thermometer with bone marrow transplant patients. Psycho-oncology 2006;15:604-12.

21. Hurria A, Li D, Hansen K, et al. Distress in older patients with cancer. Journal of clinical oncology : official journal of the American Society of Clinical Oncology 2009;27:4346-51.
22. Van Orden KA, Witte TK, Gordon KH, Bender TW, Joiner Jr TE. Suicidal desire and the capability for suicide: Tests of the interpersonal-psychological theory of suicidal behavior among adults. Journal of consulting and clinical psychology 2008;76:72-83.
23. Okuyama T, Endo C, Seto T, et al. Cancer patients' reluctance to disclose their emotional distress to their physicians: a study of Japanese patients with lung cancer. Psycho-oncology 2008;17:460-5.
24. Mack JW, Nilsson M, Balboni T, et al. Peace, Equanimity, and Acceptance in the Cancer Experience (PEACE): validation of a scale to assess acceptance and struggle with terminal illness. Cancer 2008;112:2509-17.
25. Maly RC, Frank JC, Marshall GN, DiMatteo MR, Reuben DB. Perceived efficacy in patient-physician interactions (PEPPI): validation of an instrument in older persons. Journal of the American Geriatrics Society 1998;46:889-94.
26. Degner LF, Sloan JA, Venkatesh P. The Control Preferences Scale. The Canadian journal of nursing research = Revue canadienne de recherche en sciences infirmieres 1997;29:21-43.
27. Singh JA, Sloan JA, Atherton PJ, et al. Preferred roles in treatment decision making among patients with cancer: a pooled analysis of studies using the Control Preferences Scale. The American journal of managed care 2010;16:688-96.
28. Elkin EB, Kim SH, Casper ES, Kissane DW, Schrag D. Desire for information and involvement in treatment decisions: elderly cancer patients' preferences and their physicians' perceptions. Journal of clinical oncology : official journal of the American Society of Clinical Oncology 2007;25:5275-80.
29. Mishel MH. The measurement of uncertainty in illness. Nursing research 1981;30:258-63.
30. Podsiadlo D, Richardson S. The timed "Up & Go": a test of basic functional mobility for frail elderly persons. Journal of the American Geriatrics Society 1991;39:142-8.
31. Borson S, Scanlan JM, Chen P, Ganguli M. The Mini-Cog as a screen for dementia: validation in a population-based sample. Journal of the American Geriatrics Society 2003;51:1451-4.
32. Costa D, Severo M, Fraga S, Barros H. Mini-Cog and Mini-Mental State Examination: agreement in a cross-sectional study with an elderly sample. Dementia and geriatric cognitive disorders 2012;33:118-24.
33. Katzman R, Brown T, Fuld P, Peck A, Schechter R, Schimmel H. Validation of a short Orientation-Memory-Concentration Test of cognitive impairment. The American journal of psychiatry 1983;140:734-9.
34. Zulian GB, Gold G, Herrmann F, Michel JP. Mini Nutritional Assessment and cancer. Nestle Nutrition workshop series Clinical & performance programme 1999;1:93-8, 9-100.
35. Guralnik JM, Ferrucci L, Simonsick EM, Salive ME, Wallace RB. Lower-extremity function in persons over the age of 70 years as a predictor of subsequent disability. The New England journal of medicine 1995;332:556-61.
36. Given CW, Given B, Stommel M, Collins C, King S, Franklin S. The caregiver reaction assessment (CRA) for caregivers to persons with chronic physical and mental impairments. Research in nursing & health 1992;15:271-83.
37. Jakobsson U. Using the 12-item Short Form health survey (SF-12) to measure quality of life among older people. Aging clinical and experimental research 2007;19:457-64.
38. Hanly P, Ceilleachair AO, Skally M, et al. How much does it cost to care for survivors of colorectal cancer? Caregiver's time, travel and out-of-pocket costs. Supportive care in cancer : official journal of the Multinational Association of Supportive Care in Cancer 2013;21:2583-92.
39. Ryff CD, Keyes CL. The structure of psychological well-being revisited. Journal of personality and social psychology 1995;69:719-27.

40. Galvin JE, Roe CM, Xiong C, Morris JC. Validity and reliability of the AD8 informant interview in dementia. Neurology 2006;67:1942-8.
41. Arora NK, Weaver KE, Clayman ML, Oakley-Girvan I, Potosky AL. Physicians' decision-making style and psychosocial outcomes among cancer survivors. Patient education and counseling 2009;77:404-12.
42. Epstein RM, Street RL, Jr. Shared mind: communication, decision making, and autonomy in serious illness. Annals of family medicine 2011;9:454-61.
