# Supplementary material for: Association of Oncologist-Patient Communication With Functional Status and Physical Performance in Older Adults: A Secondary Analysis of a Cluster Randomized Clinical Trial
Source: JAMA Netw Open. 2022 Mar 18;5(3):e223039. doi: 10.1001/jamanetworkopen.2022.3039 (PMC8933739; doi:10.1001/jamanetworkopen.2022.3039)
Supplement: Supplement 3. — eAppendix. [file jamanetwopen-e223039-s003.pdf]

APPENDIX X-8

CODING PROCEDURES

As part of coding procedures, a manual was developed which included definitions of age related concerns categorized within each GA domain, of who initiated the discussion, and the possible response qualities from the oncologist. Five coders each underwent 40 hours of in-person training with sub-investigators and the PI. In addition, all coders read and studied the coding manual. Coders transitioned from training to independent coding only after full consensus was met on all 25% training transcripts as a group.

The GA domains include physical performance, functional status, cognitive, comorbidity, polypharmacy, nutritional status, psychological status, and social support. Explicit discussions related to cognition (e.g., how is your memory?) were captured as well as implicit discussions (e.g., are you remembering to take your medications). Within each GA domain there are numerous age-related concerns, which are listed in the coding manual as subcodes, with the addition of an unspecified subcode for each concern. Developing the coding scheme for who initiated the concern involved identifying who initially brought up the concern during the clinic visit (e.g oncologist, patient, caregiver, other health care provider, friend, family member). For response quality coding, the coders identified whether an age-related concern was specifically asked about or only mentioned, and whether later acknowledged through a follow-up question, reflection, or validation. Then GA concerns were reviewed to detect whether or not they were appropriately addressed by the oncologist or not addressed (e.g. dismissed, ignored, shut down, minimized) at all.

The coding procedures consist of initially reading the transcript to look for the geriatric domains discussed, the identification of any age related concern, and determining who initiated those concerns during the clinic visit. The second reading of the transcript was to identify the response quality of each age related concern and any discussions of GA recommendations to address those concerns.

All coders were paired together rotating coding partners throughout the coding process to ensure groups did not drift in their coding process over time. Each coder coded independently. Then, each week met with his or her paired coder to complete one coding consensus table for each transcript. In addition, each week all five coders met and came to consensus concerning the transcripts designated for inter-rater reliability, creating a final consented coding table. Twenty percent of the transcripts were coded by all five coders to establish inter-rater reliability, with the remaining transcripts dually coded. The final consensus tables will be used to analyse the data.

Because all final codes were discussed and agreed upon by at least two trained coders, reliability and consistency of the codes throughout all observations is very high.

COACH Coder Interrater Reliability

To establish interrater reliability between all coders, 20% of all transcripts will be coded by all coders. These transcripts will be allocated over the entire duration of time coding is conducted to test for ongoing agreement among all coders. Transcripts are randomly assigned to each coder. Every 5th transcript is coded by all coders and will be used for IRR. If an overall agreement is not ≥ 70%, discrepancies will be identified and targeted training will take place. Also to prevent coding drift, the teams of coders will alternate. Review the table below to explain this strategy.

Transcript #	Coder 1	Coder 2	Coder 3	Coder 4	Coder 5
1	X	X			
2		X	X		
3			X	X	
4				X	X
5	X	X	X	X	X

Due to the conditional coding structure of this study, we will report percent agreements for 3 coding areas. These percent agreements will be calculated on the 20% of transcripts that are coded by all coders, since the remainder of transcripts are dual coded. The gold standard for the calculation of percent agreement will be the consensus coding, which will be reached by all coders.

% Agreement for 3 Coding Areas

(1) First is the percent of agreement on the **number of geriatric concerns mentioned** in the transcript, without regard to who initiated the conversation. This is calculated on the difference score between the coder and consensus and a percent of those coded correctly is divided by those coded incorrectly. The equation is as follows: consensus # - abs(coder # - consensus #) / consensus #) x 100). An average of all individual coders' agreements will be reported.

(2) Second is the percent agreement on **the category of geriatric domain discussed.** This will be calculated by each individual coder agreement with the consensus of whether each geriatric domain (a total of 8) was present or absent. An average of all coder agreements will be computed.

(3) Lastly is the percent agreement on **the physician's concern response quality.** Response quality will be considered for only the same concerns that all coders coded for individually. The agreement will be calculated based on the coder's agreement with the consensus codes on the three response quality categories: appropriately acknowledged, appropriately addressed, and dismissed. An average of all coder agreements will be computed.

Computation

We will compute percent agreement scores for the three defined areas. Information will be available in real time to allow for retraining if necessary and ongoing monitoring. All scores will be maintained in a data file and summary reports will be computed at study end (or when required). The table below provides an example of how all three percent agreements will be calculated. The three percent agreements are highlighted in blue.

Note the computation varies because area one is a count variable, and areas two and three are all dichotomous variables (yes/no). For the number of geriatric concerns mentioned, percent agreement is calculated on the difference score between the coder and consensus and a percent of those coded correctly divided by those coded incorrectly.

Percent Agreement	Variable	Coder A	Coder B	Coder B	Consensus	A and Consensus	B and Consensus	C and Consensus	% Agreement
**# of Concerns**	Number of geriatric concern mentioned	7	5	5	5	0%	100%	100%	67%
**Geriatric Domain Mention**	Functional Status	1	0	1	1	100%	0%	100%	67%
	Nutritional Status	1	1	1	1	100%	100%	100%	100%
	Cognition	0	0	0	0	100%	100%	100%	100%
	Physical Performance	1	1	1	1	100%	100%	100%	100%
	Comorbidity	0	0	0	0	100%	100%	100%	100%
	Polypharmacy	1	0	1	1	100%	0%	100%	67%
	Social Support	0	1	1	1	0%	100%	100%	67%
	Psychological Status	0	1	0	0	100%	0%	100%	67%
									83%
**Response Quality: Acknowledged**	Concern 1	1	1	1	1	100%	100%	100%	100%
	Concern 2	0	0	0	0	100%	100%	100%	100%
	Concern 3	1	1	1	1	100%	100%	100%	100%
	Concern 4	1	1	1	0	0%	0%	0%	0%
	Concern 5	0	0	1	1	0%	0%	100%	33%
**Response Quality: Addressed**	Concern 1	0	0	0	0	100%	100%	100%	100%
	Concern 2	0	0	0	0	100%	100%	100%	100%
	Concern 3	0	1	0	1	0%	100%	0%	33%
	Concern 4	1	1	1	1	100%	100%	100%	100%
	Concern 5	0	0	0	0	100%	100%	100%	100%
**Response Quality: Dismissed**	Concern 1	0	0	0	0	100%	100%	100%	100%
	Concern 2	0	0	0	0	100%	100%	100%	100%
	Concern 3	0	1	0	1	0%	100%	0%	33%
	Concern 4	1	1	1	1	100%	100%	100%	100%
	Concern 5	0	0	0	0	100%	100%	100%	100%
**General**	Mention of Geriatric Assessment	1	0	1	1	100%	0%	100%	67%

**Interventions**	Assess values/goals for treatment outcome	1	1	1	1	100%	100%	100%	100%
	Elicit caregiver perspective/input	0	0	0	0	100%	100%	100%	100%
	Discussed health care proxy	1	1	1	1	100%	100%	100%	100%
	Goals of care preferences	0	0	0	0	100%	100%	100%	100%
	Confirm health care proxy in chart	0	1	0	0	100%	0%	100%	67%
	List emergency contacts in chart	0	0	1	0	100%	100%	0%	67%
	Confirm Advanced Directives in chart	0	0	0	0	100%	100%	100%	100%
	Discuss advanced directive	1	1	1	1	100%	100%	100%	100%
	Change chemo regimen	0	0	1	1	0%	0%	100%	33%
									81%
