# Supplementary material for: Association of Oncologist-Patient Communication With Functional Status and Physical Performance in Older Adults: A Secondary Analysis of a Cluster Randomized Clinical Trial
Source: JAMA Netw Open. 2022 Mar 18;5(3):e223039. doi: 10.1001/jamanetworkopen.2022.3039 (PMC8933739; doi:10.1001/jamanetworkopen.2022.3039)
Supplement: Supplement 4. — Data Sharing Statement [file jamanetwopen-e223039-s004.pdf]

Data Sharing Statement

Jensen-Battaglia. Association of Oncologist-Patient Communication With Functional Status and Physical Performance in Older Adults. *JAMA Netw Open*. Published March 18, 2022. doi:10.1001/jamanetworkopen.2022.3039

Data
Data available: Yes
Data types: Deidentified participant data, Data dictionary
How to access data: supriya_mohile@urmc.rochester.edu
When available: With publication

Supporting Documents
Document types: None

Additional Information
Who can access the data: Researchers whose proposed use of the data has been approved
Types of analyses: For any purpose
Mechanisms of data availability: With investigator support, after approval of a proposal, and with a signed data access agreement
